# An Unprecedented 4,8-Cycloeudesmane, Further New Sesquiterpenoids, a Triterpene, Steroids, and a Lignan from the Resin of *Commiphora myrrha* and Their Anti-Inflammatory Activity In Vitro

**DOI:** 10.3390/molecules29184315

**Published:** 2024-09-11

**Authors:** Anna Unterholzner, Katrin Kuck, Anna Weinzierl, Bartosz Lipowicz, Jörg Heilmann

**Affiliations:** 1 Institute of Pharmaceutical Biology, University of Regensburg, Universitätsstr. 31, D-93053 Regensburg, Germany; anna.unterholzner@chemie.uni-regensburg.de (A.U.);; 2 Repha GmbH Biologische Arzneimittel, Alt-Godshorn 87, D-30855 Langenhagen, Germany

**Keywords:** *Commiphora myrrha*, Burseraceae, sesquiterpene lactone, cycloeudesmane, phytosterol, lignan, anti-inflammatory

## Abstract

Myrrh has a long tradition in the treatment of inflammatory diseases. However, many of its (active) constituents are still unknown. In the present study, secondary metabolites were isolated from an ethanolic extract by various separation methods (liquid–liquid partition, silica and RP18 flash chromatography, CPC, and preparative HPLC), their structures were elucidated with NMR spectroscopy and mass spectrometry, and the selected compounds were tested for their effect on LPS-induced NO production by RAW 264.7 murine macrophages. Among the isolated substances are 17 sesquiterpenes (**1**–**17**) including the first 4,8-cycloeudesmane (**1**), a triterpene (**38**), two phytosterols (**39**, **40**) and one lignan (**43**), which were previously unknown as natural products. Numerous compounds are described for the first time for the genus *Commiphora*. Eight of the eleven compounds tested (**1**, **29**, **31**, **32**, **34**–**37**) showed a statistically significant, concentration-dependent weak to moderate anti-inflammatory effect on NO production in the LPS-stimulated RAW 264.7 macrophages in vitro. For the reference substance, furanoeudesma-1,3-diene, an IC_50_ of 46.0 µM was determined. These sesquiterpenes might therefore be part of the multi-target molecular principles behind the efficacy of myrrh in inflammatory diseases.

## 1. Introduction

The small trees or shrubs of the genus *Commiphora* (Burseraceae) grow in East Africa, the Arabian Peninsula, and India [1]. Their secreted oleo-gum resin, especially from *Commiphora myrrha* (Nees) Engl., is known as myrrh and consists of water-soluble gums, alcohol-soluble resin, and a volatile oil [2]. Numerous natural compounds from the resin have been described; sesquiterpenes and triterpenes were the predominant classes, each with multiple scaffolds [3]. Due to spectroscopic advances in the recent years, the identification of several dimeric sesquiterpenes has been described since 2018 [4,5,6]. Steroids are mainly found in *C. mukul* but have also been detected in *C. myrrha* [3,7]. The first lignans described for *C. myrrha* were of the bisepoxy or furofuran structural type [8].

Myrrh has been used since ancient times not only for cultural and religious purposes but also for the treatment of a variety of, often inflammatory, diseases [9]. Modern studies have demonstrated, in particular, the anti-inflammatory [10,11], anticancer [12], antibacterial [13,14] and analgesic [15,16] effect of the gum resin of *C. myrrha*. In addition, myrrh is established in the treatment of ulcerative colitis (UC) due to its positive effects in animal models [17] and in a clinical study in combination with coffee charcoal and chamomile flower extract [18]. Numerous studies have already shown that terpenoids isolated from myrrh resin suppress inflammation in various experiments [11,19,20,21]. Despite the promising results and the indications that multiple sesquiterpenes are the main bioactive substances of myrrh [22], there are still unidentified active compounds and the molecular mechanisms of action are still insufficiently understood.

In the present study, an ethanolic extract of the drug was fractionated and several secondary metabolites were isolated. Furthermore, the anti-inflammatory effect of the selected substances in an in vitro model using RAW 264.7 macrophages is reported. Similar to human macrophages, this murine cell line produces nitric oxide (NO) as an immunomodulatory and toxic defence molecule during inflammation [23]. In an aqueous solution, NO autoxidizes to nitrite and can be quantified spectroscopically after staining with the Griess reagent. NO is one of the pathologically elevated factors in UC patients [24]. First, the evidence suggests that myrrh suppresses the inflammatory response in the in vitro model used by mediating haem oxygenase-1 expression [25]. In addition, myrrh suppressed the inflammation by influencing the MAPK signalling pathway in another cell model [11].

The aim of this work is therefore, on the one hand, to contribute to the phytochemical characterization of the secondary metabolites in myrrh, which is far from complete since it is a mixture of numerous minor components; and, on the other hand, to get a better understanding of its pharmacological principles of action.

## 2. Results

Myrrh resin was extracted with ethanol and the extract was split into two parts by a liquid–liquid partition between methanol and *n*-heptane. Both fractions were further separated using various chromatographic techniques such as flash chromatography, centrifugal partition chromatography (CPC), and preparative HPLC. The structures of the pure compounds were elucidated with NMR, mass spectrometry using liquid chromatography and high-resolution electrospray ionisation (HRESIMS), or gas chromatography combined with atmospheric pressure chemical ionisation (GC-APCI), and circular dichroism spectroscopy (CD). Thus, 37 sesquiterpenes, 1 triterpene, 4 phytosterols, and 1 lignan were identified, including 21 new natural products (**1**–**17**, **38**–**40**, and **43**) and 12 compounds that have not yet been described for the genus *Commiphora*. Known compounds that have not been described for *Commiphora* so far were elucidated by their NMR data to be serralactone A (**18**) [26], 1β,8β-dihydroxyeudesman-3,7(11)-dien-8α,12-olide (**19**) [27], neolitacumone B (**20**) [28], neolitacumone A (**21**) [27], 3-oxo-5α*H*,8β*H*-eudesma-1,4(15),7(11)-trien-8,12-olide (**22**) [29], (+)-eudebeiolide B (**23**) [30], linderolide I (**24**) [31], 1β,8β-dihydroxyeudesma-4,7(11)-dien-8α,12-olide (**25**) [32], istanbulin B and A (**26** and **27**) [33], chloraniolide A (**28**) [34], and 7-ketostigmasterol (**42**) [35]. For compounds **24** [31] and **28** [34], the additional literature data could be corrected. Stigmasta-5,22*E*-diene-3β,11α-diol (**41**) has been isolated from another species before, namely *C. mukul* [36].

In addition to compounds **1** and **21**, the following compounds known for *C. myrrha* were isolated and tested on their anti-inflammatory effects in the Griess assay: 2*S*-methoxy-4*S*-furanogermacra-1(10)*E*-en-6-one (**29**) [37,38], 2β-methoxyglechomanolide (**30**) [5], 8-*epi*-2β-methoxyglechomanolide (**31**) [5], isohydroxylindestrenolide (**32**) [39], hydroxylindestrenolide (**33**) [40], dehydrolindestrenolide (**34**) [41], 9-oxo-9,10-seco-isolindestrene (**35**) [5], alismol (**36**) [42], and commiterpene E (**37**) [5]. Furthermore, the known myrrh metabolites, 1(10)*E*,4*E*-furanodienone [43], 2-acetoxyfuranodiene [44], curzerenone [43], myrrhone [45] and 1*R*,2*R*-epoxy-4*S*-furanogermacr-10(14)-en-6-one [38,46] were isolated.

### 2.1. Sesquiterpenoids

**Figure 1:**
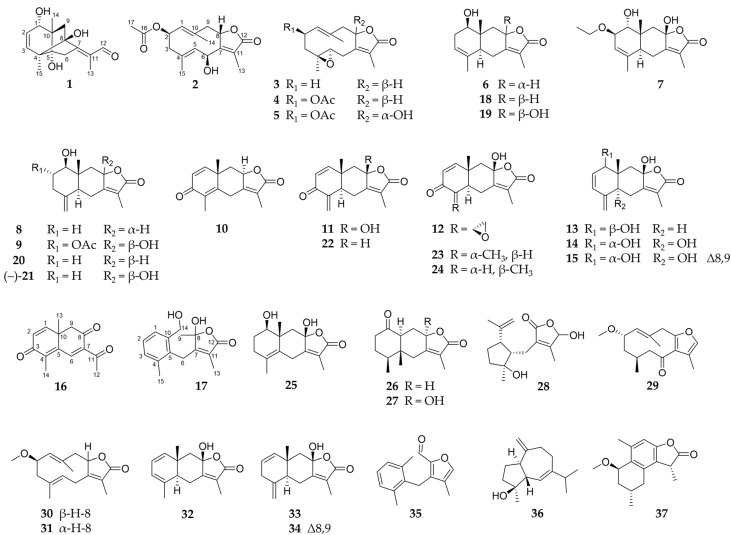
Chemical structures of the isolated sesquiterpenoids **1**–**37**.

The isolated sesquiterpenoids **1**–**37** are shown in Figure 1.

**Figure 2:**
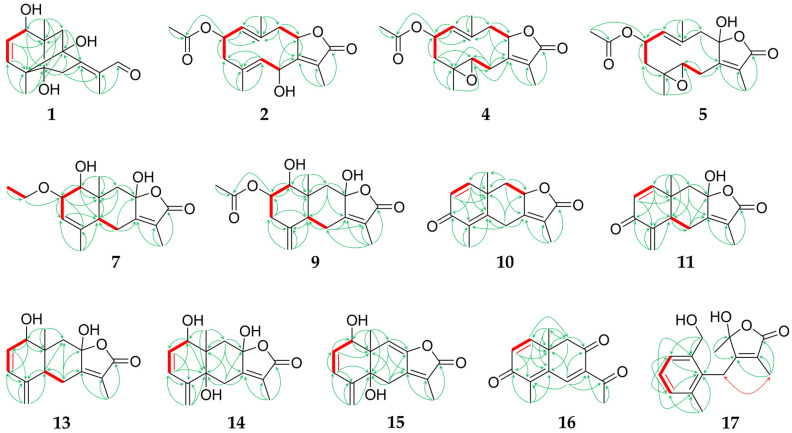
Key HMBC (green arrows) and COSY correlations (red, bold or red arrow) of sesquiterpenoid compounds with new constitution.

**Figure 3:**
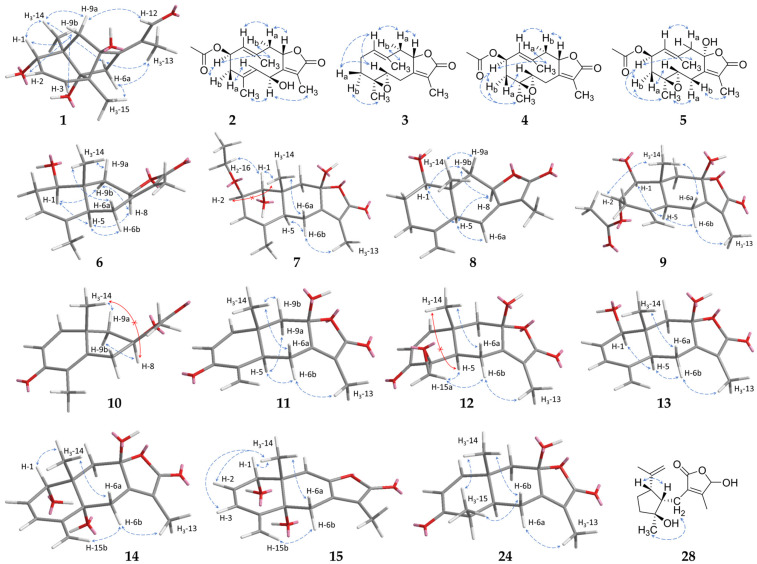
Key NOESY correlations (blue arrows, missing ones: red arrows) of chiral sesquiterpenoid compounds with new constitution or configuration. Three-dimensional structures were calculated using Chem3D where reasonable.

Compound **1**, obtained as a colourless oil, showed a molecular formula of C_15_H_20_O_4_, as determined by HRESIMS at *m*/*z* 265.1435 [M+H]^+^ (calc. 265.1434). The NMR data of **1** (1D: Table 1, 2D: Figure 2 and Figure 3, original spectra in Supplementary Materials Figures S1–S3) displayed 15 carbon resonances assignable to three methyls, two sp^3^ methylenes, one oxygenated sp^3^ methin, two sp^2^ olefinic methins, an aldehyde moiety, and six quaternary carbons (two aliphatic, two hydroxylated, and two olefinic). There were six degrees of unsaturation evident in **1**, of which three were represented by one aldehyde group and two double bonds; therefore, the molecule is tricyclic. Since the COSY experiment only showed correlations between H-1/H-2 and H-2/H-3, the other hydrogenated carbons were neighboured by quaternary carbons, which was confirmed by the splitting pattern of their ^1^H NMR signals. The HMBC correlations (Figure 2) of the exocyclic methyls H_3_-14 and H_3_-15 with the carbons adjacent to them via one and two bonds indicated the first cyclohexene ring. Further analysis of the 1D and 2D NMR data revealed an eudesmane-type skeleton with an additional bridging bond between C-4 and C-8 indicated by the HMBC signals between H_3_-15/C-8 and H-9a/C-4. This structural type, which has not been described before, can be named as 4,8-cycloeudesmane. The HMBC cross-peaks also allowed for the assignment of an exocyclic 1-oxo-isopropyliden group at C-7. The relative stereochemistry of **1** was determined by its NOESY correlations (Figure 3). It was substantially conditioned by the bridged ring system, which was again confirmed by the correlations of H-9b with H-1 to H-3. The exocyclic double bond was *Z*-configured according to the signals between H_2_-6/H_3_-13, on the one hand, and H-9a/H-12, on the other hand. Consequently, the structure of 1β,5,8-trihydroxy-4α,8-cycloeudesma-2,7(11)*Z*-dien-12-al (**1**) was established, as shown in Figure 1.

Compounds **2**–**5** can be summarized as the oxygenated derivatives of glechomanolide [47], with **2**, **4**, and **5** being derivatives of the known myrrh metabolite 2β-acetyloxyglechomanolide [39], and the molecular model calculated in the cited publication enabled the relative stereochemical characterization of the distant H-2 and H-8. According to the calculations and the observed signals of the substances, the characteristic NOESY correlations of 2β-acetyloxyglechomanolide derivatives were identified as follows: H_3_-14 couples with H-2 and H-9α on the one side, and H-9β couples with H-1 and H-8 on the opposite side of the germacrene ring. In contrast, the C-8 epimeric compound as described by Greve et al. [39] showed cross-peaks between H_3_-14 and not only H-2 but also, characteristically, H-8. The replacement of the double bond at C-4 by an epoxidation in compounds **3**–**5** did not essentially change the conformation of the ten-membered germacrene ring, as observed in the corresponding NOESY data as well as described in the literature [48]. Compound **3** was lacking the 2-acetyloxy moiety, but its relative configuration could be established by comparing its NOESY signals with the otherwise identical compound **4**.

Compound **2** was obtained as a colourless oil and its molecular formula of C_17_H_22_O_5_ was determined by HRESIMS at *m*/*z* 307.1545 [M+H]^+^ (calc. 307.1540). Analysis of its 1D and 2D NMR spectra revealed that **2** is a C-6 hydroxylated derivative of 2β-acetyloxyglechomanolide [39] as shown by its chemical shifts (*δ*_H_ 5.53 (d, H-6); *δ*_C_ 65.8). In accordance with the calculated molecular models in the literature and the explanation above, the NOESY signals (Figure 3) indicated the relative stereochemical configurations at C-2 and C-8. They were furthermore confirmed by the CD spectrum of **2** with a prominent negative cotton effect at λ_min_ 214 nm (Δε –13.0; Figure S24) that was consistent with the spectra of 2β-acetyloxy- and 2β-methoxyglechomanolide in the literature [5]. The NOESY correlations of H-6 with H_3_-13 but not H-8 confirmed the relative stereochemical configuration at C-6. Therefore, **2** was identified as 2β-acetyloxy-6β-hydroxyglechomanolide.

Compound **3** was isolated as a yellowish oil in mixture with compounds **4** and **22**, and its molecular formula of C_15_H_20_O_3_ was determined by HRESIMS at *m*/*z* 249.1485 [M+H]^+^ (calc. 249.1491). Investigation of its 1D and 2D NMR data revealed a diastereomeric derivate of 4α,5α-epoxy-1(10),7(11)-dienegermacr-8α,12-olide [49] with the opposite configuration at C-4 as the only difference. The associated *trans*-configuration of the epoxide in **3** was shown by NOESY correlations between H-3a/H-5, on the one hand, and H-3b/H_3_-15, on the other hand (Figure 3). The determination of the relative configuration at C-8 was enabled by the corresponding NOESY correlations to compound **4**, respectively, as generally described above and the cross-peaks of H-1 and H-5. In this way, **3** was elucidated as 4β,5α-epoxyglechomanolide.

Compound **4**, isolated as a yellowish oil in mixture with compounds **3** and **22**, showed a molecular formula of C_17_H_22_O_5_ as determined by HRESIMS at *m*/*z* 307.1543 [M+H]^+^ (calc. 307.1540). As indicated by its 1D and 2D NMR data, it represents the acetylated derivate of **3**, as well as the epoxidated derivative of 2β-acetyloxyglechomanolide [39]. NOESY signals as described above showed that **4** featured the same relative configuration at C-2 and C-8 (Figure 3). Furthermore, the correlations of H-3b with H-2 and H_3_-15, on the one hand, and H-3a with H-5, on the other hand, helped to establish the *trans*-configuration of the epoxide. Consequently, the structure of **4** was elucidated as 2β-acetyloxy-4β,5α-epoxyglechomanolide.

Compound **5**, obtained as a white solid, had a molecular formula of C_17_H_22_O_6_ as determined by HRESIMS at *m*/*z* 323.1492 [M+H]^+^ (calc. 323.1489). Its 1D (Table 2) and 2D NMR spectra indicated a structure similar to **4** that was distinguished from it by an additional, α-oriented hydroxy group at C-8 (*δ*_C_ 106.0). Its relative configuration was determined based on the NOESY correlations between H-6b with H_3_-13, on the one hand, and H-6a with H_3_-15, on the other hand (Figure 3). The configuration corresponding to 8-*epi*-2β-methoxyglechomanolide and 8-*epi*-2β-acetyloxyglechomanolide [5] was confirmed by its similar CD spectrum showing a maximum at λ (Δε) at 221 nm (+5.9) and a minimum at 245 nm (−5.0; Figure S24). The structure of **5** was thus identified as 2β-acetyloxy-4β,5α-epoxy-8-*epi*-hydroxyglechomanolide.

Compound **6** was obtained as a colourless oil and its molecular formula of C_15_H_20_O_3_ was determined by HRESIMS at *m*/*z* 249.1491 [M+H]^+^ (calc. 249.1485). Evaluation of its 1D and 2D NMR data revealed the constitution of serralactone A [26] that was also isolated separately as compound **18**. However, the NOESY correlations (Figure 3) between H-5 and H-8 of **6** indicated the opposite configuration at C-8, such that **6** was determinable as 8-*epi*-serralacton A.

Compound **7**, isolated as colourless oil, had a molecular formula of C_17_H_24_O_5_ as determined by HRESIMS at *m*/*z* 309.1698 [M+H]^+^ (calc. 309.1697). Its NMR data showed signals for an ethyloxy substituent (*δ*_H_ 3.55/3.65 (dq/dq, H_2_-16), 1.17 (dd, H_3_-17); *δ*_C_ 65.8, 15.9, respectively) and the HMBC cross-peaks between H_2_-16 and C-2 revealed its position (Figure 2). Apart from this moiety, the constitution as well as parts of the configuration of **7** were elucidated to be the same as for compound **19**, which is known as 1β,8β-dihydroxyeudesman-3,7(11)-dien-8α,12-olide [27]. The NOESY correlations of H-1 with H_3_-14 and H_2_-16 indicated the different relative configuration of **7** at C-1 (Figure 3). Notably, the ^1^H NMR signal of H-1 appeared as a singlet despite its neighbouring hydrogen, which is explainable by their dihedral angle causing a coupling constant that is too small for resolution in the spectrum. Consequently, **7** can be named 1α,8β-dihydroxy-2β-ethyloxyeudesma-3,7(11)-dien-8α,12-olide.

Compound **8**, obtained as a yellowish oil, showed a molecular formula of C_15_H_20_O_3_ as determined by HRESIMS at *m*/*z* 249.1490 [M+H]^+^ (calc. 249.1491). The structure elucidated by its NMR spectra (Table 3) represents the H-8 epimer of neolitacumone B [28] that was also isolated as compound **20**. Based on the NOESY cross-peaks between H-5 and H-8 (Figure 3) that deviate from neolitacumone B, compound **8** was identified as 8-*epi*-neolitacumone B.

Compound **9** was obtained as a white solid and its molecular formula of C_17_H_22_O_6_ was determined by HRESIMS at *m*/*z* 321.1344 [M–H]^–^ (calc. 321.1344). Examination of its 1D and 2D NMR data revealed the C-2 acetylated derivative of neolitacumone A [27], which was also isolated as compound **21**. Therefore, the structure of 2α-acetyloxyneolitacumone A (**9**) was established as shown in Figure 1.

Compound **10**, obtained as yellowish oil, showed a molecular formula of C_15_H_16_O_3_ as determined by HRESIMS at *m*/*z* 245.1174 [M+H]^+^ (calc. 245.1172). The 1D and 2D NMR spectra revealed a so far unnamed and only synthetically obtained semichinoid eudesmanolide [50]. As only its ^1^H NMR data have been published so far, the spectroscopic data of **10** were now completed during this study. Analogous to the known 3-oxo-5α*H*,8β*H*-eudesma-1,4(15),7(11)-trien-8,12-olide (**22**) [29], compound **10** was systematically named 3-oxo-8α*H*-eudesma-1,4,7(11)-trien-8,12-olide.

Compound **11** was obtained as a colourless oil and its molecular formula was determined to be C_15_H_16_O_4_ by HRESIMS analysis at *m*/*z* 261.1126 [M+H]^+^ (calc. 261.1121). Similar NMR data revealed an hydroxy-derivate of 3-oxo-5α*H*,8β*H*-eudesma-1,4(15),7(11)-trien-8,12-olide (**22**) [29], which was indicated by the downfield shifted signal of C-8 at *δ*_C_ 105.0. Compound **11**, which was consequently identified as 3-oxo-8β-hydroxy-5α*H*-eudesma-1,4(15),7(11)-trien-8,12-olide, could possibly represent a biosynthetic interstage between compounds **23** and **24** during the isomerization of the methyl C-15.

Compound **12**, obtained as yellowish oil, showed a molecular formula of C_15_H_16_O_5_ as determined by HRESIMS at *m*/*z* 277.1067 [M+H]^+^ (calc. 277.1071). Its constitution was determined by its 1D (Table 4) and 2D NMR data to be corresponding to linderolide J [31]. Notable was a deviation in the colour of the HSQC signals of the geminal H_2_-15, both of which were blue. Examination of the NOESY correlations (Figure 3) of compound **12** revealed a diastereomer with an opposite configuration at the spiro carbon C-4, which was indicated by the signals between H-5 and H-15a in the exocyclic oxirane ring. Thus, **12** was elucidated to be 4-*epi*-linderolide J.

Compound **13**, obtained as a white solid, showed a molecular formula of C_15_H_18_O_4_ as determined by HRESIMS at *m*/*z* 263.1277 [M+H]^+^ (calc. 263.1278). By its 1D and 2D NMR spectra, a derivative of neolitacumone A (**21**) was elucidated with an additional double bond in the position C-2 as indicated by the chemical shifts. Similar to **7**, the following deviations from the usual ^1^H NMR signal multiplicities were observed: H-1 and H-2 showed reductions to a singlet and a doublet, respectively, due to a very small coupling constant. In contrast, H-3 appeared as doublet of doublet with H-1 via the double bond, as established by an intense COSY cross-peak; however, this coupling again did not show up in the H-1 multiplicity. The positions of the carbons in this order were established by the HMBC signal of H_2_-15 with C-3 (Figure 2), as well as by the NOESY signals between H-1/H-2 and H-3/H-15b (Figure 3). For compound **13**, the name 1β,8β-dihydroxy-5α*H*-eudesma-2,4(15),7(11)-trien-8,12-olide is proposed.

Compound **14**, obtained as a white solid, showed a molecular formula of C_15_H_18_O_5_ as determined by HRESIMS at *m*/*z* 279.1226 [M+H]^+^ (calc. 279.1227). Investigation of its 1D and 2D NMR data revealed a trihydroxylated eudesmanolide differing from **13** by an additional hydroxy group at C-5 (*δ*_C_ 78.2), as well as the relative stereochemical configuration at C-1, according to the NOESY correlation of H-1 with H_3_-14 (Figure 3). The remaining stereo centres were also determined by the NOESY cross-peaks in combination with molecular models obtained with Chem3D; for the opposite configuration at C-5, the calculated distance of 4,9 Å between H_3_-14 and H-6a would prohibit the intense NOESY signal that was observed. Consequently, compound **14** was identified as 1α,5α,8β-trihydroxyeudesma-2,4(15),7(11)-trien-8,12-olide.

Compound **15**, obtained as a yellowish oil, showed a molecular formula of C_15_H_16_O_4_ as determined by HRESIMS at *m*/*z* 261.1125 [M+H]^+^ (calc. 261.1121). The 1D (Table 5) and 2D NMR data suggested a molecule related to **14**, which differs from it by the elimination of a water molecule, resulting in a double bond between C-8 (*δ*_C_ 147.9) and C-9 (*δ*_C_ 112.1). The relative stereochemical configuration at C-5, respectively, the *trans* configuration of the annulated cyclohexenes, could be determined by the NOESY signals of H_3_-14 with H-1 and H-2, in accordance with their distance being less than 4 Å only in this configuration according to the Chem3D calculations (Figure 3). Therefore, the structure of 1α,5α-dihydroxyeudesma-2,4(15),7(11),8-tetraen-8,12-olide (**15**) was established as shown in Figure 1.

Compound **16** was obtained as a yellowish oil and showed a molecular formula of C_14_H_14_O_3_ as determined by HRESIMS at *m*/*z* 231.1017 [M+H]^+^ (calc. 231.1016). Its 1D and 2D NMR data indicated a norsesquiterpenoid structure similar to tussfarfarin A [51] with a missing C-13. The measured NMR spectra showed an additional double bond between C-1 and C-2 (*δ*_H_ 7.08 (d, H-1), 6.34 (d, H-2); *δ*_C_ 155.6, 126.4, respectively) as well as a keto group at C-8 (*δ*_C_ 194.8) as structural differences to tussfarfarin A. Consequently, the molecule was identified as 1,2-anhydro-8-oxo-tussfarfarin A (**16**).

Compound **17** was isolated as a white solid and had a molecular formula of C_15_H_18_O_4_ as determined by HRESIMS at *m*/*z* 263.1274 [M+H]^+^ (calc. 263.1278). As indicated by its NMR data, it was a secoeudesmanolide similar to the previously described 9,10-seco-isolindestrenolide [5], differing in two additional hydroxylations at C-8 and C-14. Therefore, the name 8,14-dihydroxy-9,10-seco-isolindestrenolide is suggested.

Identical NMR data revealed **21** to be neolitacumone A, which showed a positive rotation when isolated from *Neolitsea acuminatissima* [27]. The measured optical rotation has an opposite sign (−7.3, in MeOH). Therefore, the isolation of (–)-neolitacumone A is postulated.

Compound **24**, obtained as yellowish oil, showed a molecular formula of C_15_H_18_O_4_ as determined by HRESIMS at *m*/*z* 263.1281 [M+H]^+^ (calc. 263.1278). It was established by its 1D and 2D NMR signals to have the chemical structure and relative configuration of linderolide I [31]. As key NOESY signals for the relative stereochemical characterisation, correlations between H_3_-14 and H_3_-15 with H-6b, on the one hand, and H-6a and H_3_-13, on the other hand, were used to determine C-8 (Figure 3). However, the ^1^H and ^13^C NMR signals (both in CDCl_3_) as well as the CD spectrum (Figure S25) are deviant from the literature data. Therefore, the determination of the configuration at C-8 performed by Liu et al. [31] by the interpretation of the CD spectrum has to be doubted.

For compound **28**, obtained as a yellowish oil, a molecular formula of C_15_H_22_O_4_ was determined by HRESIMS at *m*/*z* 267.1595 [M+H]^+^ (calc. 267.1591). Its NMR data were identical to those of chloraniolide A [34], and the elucidation revealed a secoguaianolide with the same constitution. Thereby, the missing HSQC signal between C-12 and H-12 stood out; however, H-12 showed COSY and NOESY correlations with H_3_-13 (Figure 3). Regarding the relative stereochemical configuration, the NOESY correlations of H_3_-15 were more intense with H_2_-6 than with H-5. Therefore, the at C-4 epimeric structure of chloraniolide A published by Xu et al. [34] was determined to be its true configuration, so that its structure can be corrected. The stereo centre at C-12 cannot be determined based on the NOESY data due to its distance, as in the cited publication.

### 2.2. Further Structural Types

**Figure 4:**
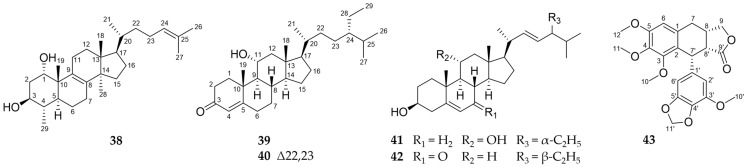
Chemical structures of the isolated triterpene (**38**), the phytosterols (**39**–**42**), and the lignan (**43**).

The structures of the isolated triterpene (**38**), the phytosterols (**39**–**42**) characterized by an extended side chain, and the lignan (**43**) are presented in Figure 4.

**Figure 5:**
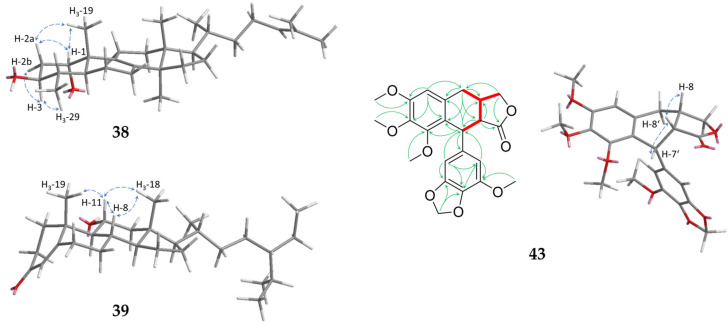
Key NOESY (blue arrows) correlations of compounds **38**, **39**, and **43** as well as HMBC (green arrows) and COSY correlations (red, bold) of **43**.

Compound **38** was obtained as a white solid. Its molecular formula of C_29_H_48_O_2_ was determined by GC-APCI at *m*/*z* 429.3727 [M+H]^+^ (calc. 429.3727). The NMR data (1D: Table 6) of the isolated compound showed a lot of similarities to the already known 29-norlanost-8,24-dien-1α,2α,3β-triol [52]. In contrast to this substance, **38** showed no hydroxylation at C-2 (*δ*_H_ 1.67/2.08 (ddd/m, H_2_-2); *δ*_C_ 36.3). Thus, the substitution pattern in the A ring matches the other triterpenes isolated from *C. myrrha* like cycloartan-24-ene-1α,3β-diol [53]. Due to strong NOESY signals below the ring level between H-3, H-2b, and H-29 as well as above the ring level between H-1, H-2a and H-19 (Figure 5), a relative 1α,3β-configuration could be assigned to the substance in accordance with the similar molecules mentioned above. To the best of our knowledge, this is the first description of this triterpene and, in accordance with trivial nomenclature, the name 29-norlanost-8,24-dien-1α,3β-diol is suggested.

Compound **39**, obtained as a colourless oil, showed a molecular formula of C_29_H_48_O_2_ as determined by GC-APCI at *m*/*z* 429.3728 [M+H]^+^ (calc. 429.3727). The NMR data of **39** showed great similarities to previously isolated phytosterols [54] but also differences in the hydroxylation pattern. Whereas the substances described in the literature have a hydroxy group in 12β-position, this moiety is shifted to C-11 in **39**. This is indicated by COSY correlations between H-11 and H-9, while the signals between H_2_-12 and H-9 are missing. The HMBC data display a similar pattern with strong correlations of H-12a (*δ*_H_ 1.24) to C-18 and H-12b (*δ*_H_ 2.30) to C-14. Thus, the hydroxy group can be unambiguously located at C-11. Based on strong NOESY correlations above the ring level between H-11 and the protons H-8, H_3_-18, and H-19, an α-configuration at position 11 could be established (Figure 5). Thus, **39** was elucidated to be 11α-hydroxysitost-4-en-3-one.

Compound **40** was obtained as a white solid and its molecular formula of C_29_H_46_O_2_ determined by GC-APCI at *m*/*z* 427.3570 [M+H]^+^ (calc. 427.3571). Its NMR data show many similarities to **39** indicating the same substitution pattern in the steroidal annulated ring system. In contrast, the signals of the side chain included typical chemical shifts in the lower field, indicating the presence of another double bond between C-22 and C-23 as also present in **41** and **42**. Therefore, the structure of 11α-hydroxystigmast-4-en-3-one was established as shown in Figure 4.

Compound **43** was obtained as a yellowish solid and showed a molecular formula of C_23_H_24_O_8_ as determined by HRESIMS at *m*/*z* 429.1547 [M+H]^+^ (calc. 429.1544). On the basis of its NMR spectra (Table 7), it was elucidated as a stereoisomer of a previously synthetically obtained multiple methoxylated aryltetralin lignanolide [55]. Intense NOESY correlations between H-8′ and H-8 as well as H-7′ indicated their positions on the same side of the ring (Figure 5). Taking into account the biosynthetic origin as dimeric phenylpropanoid, we suggest the name *rel*-7′-(3′-methoxy-4′,5′-methylendioxyphenyl)-3,4,5-trimethoxy-7,8*S*,7′*R*,8′*R*-tetrahydronaphtho[2,3-c]furan-9′(3*H*)-one for **43**.

### 2.3. Activity against NO Production

**Figure 6:**
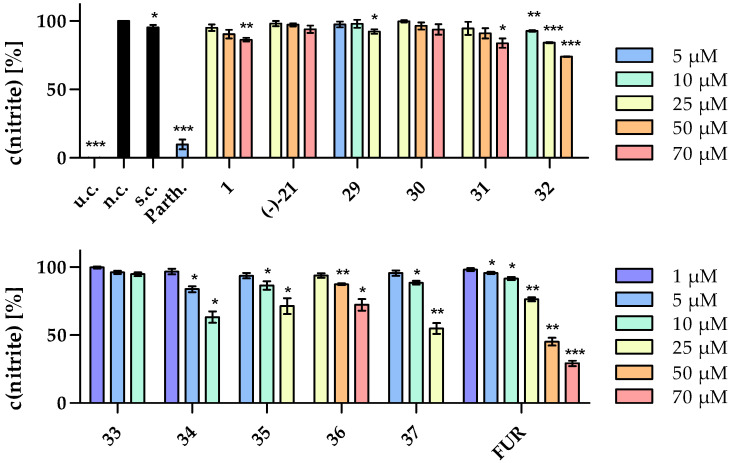
Influence of compounds **1**, (−)-**21**, **29**–**37**, and furanoeudesma-1,3-diene (FUR) on LPS-induced (1 µg/mL) NO production by RAW 264.7 macrophages in the Griess assay. The tests were performed in hexaplicates including an untreated control (u.c., medium without LPS), a negative control (n.c.), a solvent control (s.c., 0.1% DMSO, *v*/*v*), and a positive control (parthenolide, 5 µM). Data are presented as mean ± SEM, * *p* < 0.05, ** *p* < 0.01, *** *p* < 0.001 vs. n.c. (student’s *t*-test, *n* = 3).

For the investigation of the anti-inflammatory principle of myrrh, sesquiterpenes obtained in a sufficient HPLC-DAD purity (>90%) (**1**, **21**, **29**–**37**; ^1^H and ^13^C NMR spectra in Supplementary Materials Figures S1 and S28–S37) as well as the reference substance, furanoeudesma-1,3-diene (FUR), were selected. They were evaluated for their activity against NO production in LPS-stimulated RAW 264.7 murine macrophages in vitro using the Griess reagent (Figure 6). Non-toxic concentrations that affect cell viability by less than 10% were previously determined by MTT assays (Figure S27).

**Figure 7:**
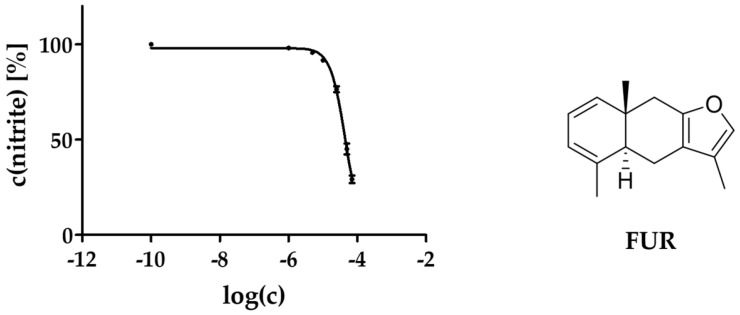
Influence of different concentrations of FUR on LPS-induced NO production by RAW 264.7 macrophages (IC_50_ = 46.0 µM). The tests were performed in hexaplicates (*n* = 3) and the data are presented as mean ± SEM including nonlinear regression curve. c in mol/L.

Eight of the eleven tested compounds (**1**, **29**, **31**, **32**, **34**–**37**) as well as FUR showed concentration-dependent slight but significant reductions in NO release by up to 40% (**37**; Figure 6). The strongest activity was shown by compound **34** with a reduction in NO synthesis to 63% at a concentration of 10 µM. Interestingly, FUR was the fourth most active compound when comparing the results at a concentration of 25 µM but was the only one showing a reduction by more than 50% percent in a non-toxic concentration range. Therefore, its IC_50_ value could be calculated by nonlinear regression analysis to be 46.0 µM, which can be classified as moderate activity (Figure 7).

## 3. Discussion and Conclusions

### 3.1. Structural Diversity

The terpenoids, phytosterols, and the lignan presented here contribute to the still far from complete phytochemical characterization of myrrh and illustrate the heterogeneity of the resin. Most of the compounds isolated from *C. myrrha* in the present work were eudesmanolides, but germacranes, a noreudesmane, a secoeudesmane, two eremophilanes, a secoguaiane, a triterpene, phytosterols and a lignan were also found. In addition, the structures varied in multiple functionalities including a rare ethyloxy substitution (**7**). With compound **1**, the first 4,8-cycloeudesmane type sesquiterpene was elucidated. Compound **16** represents the first norsesquiterpenoid, and compounds **26** and **27** were the first eremophilanes described for *Commiphora* sp. With **43**, the first aryltetralin lignan was found for *C. myrrha*. The analysis of the rich secondary metabolite spectrum is not only crucial for the quality analytics of the drug but also the basis for identifying the molecular principle of action, and it may even be useful for general drug discovery.

Overbridged ring systems as present in the new 4,8-cycloeudesmane structural type in compound **1** have been described for *C. myrrha*. With α-copaene und β-ylangene, two 5,10-cyclocadinanes are known constituents of its essential oil [56]. In other plants, cycloeudesmanes with additional covalent bonds between C-1 and C-3, C-2 and C-4, C-5 and C-7, as well as between C-6 and C-8 have been found [57,58].

The structural class of secoeudesmanes was recently discovered through our work on *C. myrrha* [21]. The introduced compound **17** is another representative of this class. Compound **28** is also formed by a cleavage step in the biosynthesis resulting in a rare secoguaiane scaffold. In addition, 7,8-secocadinanes are also known for *C. myrrha* [59].

Within the species *Commiphora*, steroids are dominating in *C. mukul* [3]. With β-sitostenone and β-sitosterol, two phytosterols were described for *C. myrrha* [7]. The four phytosterols **39**–**42** found in the present study extend the known spectrum and thus provide evidence for their possible contribution to bioactivity as a generally active structural class [60].

Further lignans of the aryltetralin type, as present in **43**, have been described for *C. incisa* [61] and *C. erlangeriana* [62]. Two of them, the erlangerins C and D, are known to be toxic and show the same substitution pattern of the aromatic rings as present in podophyllotoxin [63]. For *C. myrrha*, only lignans of the bisepoxy (or furofuran) structural type have been described so far [8].

### 3.2. Anti-Inflammatory Activity

The weakly to moderately active compounds presented might be part of the multi-target molecular principles behind myrrh’s efficacy in inflammatory diseases in general, as well as, in particular, in UC patients with increased NO levels. The results therefore support the evidence that multiple sesquiterpenes are the bioactive principles of myrrh [22]. Further investigations are necessary, however, to clarify their structure–activity relationship(s) as well as their target(s) and the principles of the effectiveness of myrrh in humans. Synergistic effects between the compounds may also be involved, as they have been described for the combination of myrrh with frankincense [22] or with chamomile flower extract and coffee charcoal [64].

Apart from our results, the known compounds **19**, **21**, **25** [32], **23** [65] and **36** [66] were described in the literature as active NO inhibitors in the Griess assay. The published data were reproduced in parts for **36**, as a significant reduction was measured; the ineffectiveness of **21** could maybe attributed to its stereochemistry as possibly the (+)-enantiomer was tested to be active by Liu et al. [32].

The active reference substance FUR represents one of the main components of *C. myrrha*’s volatile oil with a proportion of up to 30% [67,68]. Its moderate inhibitory activity on the expression of the intercellular adhesion molecule 1 (ICAM-1) in vitro has been shown [21], as well as the analgesic effects via the opioid receptors in mice [69].

The observed anti-inflammatory activity raises the question of possible common activity codetermining structural elements. α-Methylene-γ-lactone groups as present in the positive control, parthenolide, are a known feature but were not present in the tested compounds [70,71]. After comparison of the structures within the scope of our own results, the conjugated double bonds (as present in the most active **34**, **37**, **35**, FUR and **32**) could be hypothesized as an influencing structural element. For a conclusive statement, however, more experiments are needed due to the structural heterogeneity of the active substances. Initial evidence suggests that myrrh mediates the expression of haem oxygenase-1 and thus suppresses the inflammatory response in the in vitro model used [25]. However, there is likely more than one target in the inflammatory cascade from the stimulation by LPS to the final NO synthesis.

## 4. Materials and Methods

### 4.1. Chemicals

EtOH for extraction was purchased in technical quality from CSC Jäcklechemie (Nuremberg, Germany) and purified by evaporation. MeOH, *n*-heptane, ethyl acetate, dichloromethane, diethyl ether, and toluene (all pro analysis quality, p.a.) were obtained from Fisher Scientific (Hampton, NH, USA). Anisaldehyde (4-methoxybenzaldehyde for synthesis), sulfuric acid (95–97%, p.a.), acetonitrile (ACN; HPLC-grade), parthenolide, and sodium nitrite (p.a.) were purchased from Merck Chemicals (Darmstadt, Germany), as well as CD_3_OD from Deutero (Kastellaun, Germany), and *n*-hexane (p.a.), and chloroform-*d* (99.8%) from Sigma-Aldrich (St. Louis, MO, USA). Formic acid (p.a.) and dimethyl sulfoxide (DMSO, ≥99.5% for molecular biology) were provided by Carl Roth (Karlsruhe, Germany).

Roswell Park Memorial Institute (RPMI) 1640 medium (2 g/L NaHCO_3_, with and without phenol red), foetal bovine serum (FBS) Superior and L-glutamine (200 mM) were purchased from Biochrom (Berlin, Germany), and 3-(4,5-dimethylthiazol-2-yl)-2,5-diphenyltetrazoliumbromide (MTT), sodium dodecyl sulfate (SDS), as well as bacterial lipopolysaccharides (LPS, from *Escherichia coli* (055:B5)) from Sigma-Aldrich (Taufkirchen, Germany). Griess reagent was prepared from 250 mg sulfanilamide (99%, Sigma-Aldrich), 25 mg N-(1-naphtyl)-ethylenediamine-dihydrochloride (≥98%, Sigma-Aldrich), 87.5 µL *o*-phosphoric acid (p.a., 85%, Merck Chemicals), and 25 mL purified water. Furanoeudesma-1,3-diene (FUR, 96%) was provided by PhytoLab (Vestenbergsgreuth, Germany).

### 4.2. Plant Material and Extraction

The resin of *C. myrrha* (Myrrha, Ph. Eur. 2016) was provided from Lomapharm^®^ (lot NM0160, Rudolf Lohmann GmbH KG, Emmerthal, Germany). The powdered resin of *C. myrrha* (3 kg) was extracted by maceration and percolation with EtOH 96% (26 L) as described before [21], and 761.7 g dry extract were obtained.

### 4.3. Isolation

Much of the isolation process has already been published [5,21] and is therefore presented briefly and supplemented with the new methods.

#### 4.3.1. Liquid–Liquid Partition

As a first separation step, portions of the extract were dissolved in MeOH, mixed with *n*-heptane in separatory funnels, and divided into an *n-*heptane (HEP, 70.0 g) and a MeOH soluble fraction (MeOH, 328.1 g) by liquid–liquid partition.

#### 4.3.2. Flash Chromatography and CPC of the HEP Fraction

The second separation method used for the HEP fraction was silica flash chromatography (Spot flash system, Armen Instrument, Paris, France). The sample was partitioned into ten fractions (HEP1–10) using an SVP D40 silica column (13 × 4 cm, SI60 15–40 µm, 90 g, Götec Labortechnik GmbH, Bickenbach, Germany) with an *n*-hexane/ethyl acetate gradient. Fraction HEP7 was further separated by CPC and another silica chromatography as described earlier to gain compounds **30**, **31** [5], **32**, and **33** [21].

HEP8 (2951–3200 mL retention volume, 2.3 g weight) was then subjected to a centrifugal partition chromatography (CPC) using a Spot CPC device with a 250 mL rotor (Armen Instrument, Paris, France), a 510 HPLC pump (Waters GmbH, Eschborn, Germany), and a 2111 Multirac Fraction Collector (LKB-Produkter AB, Bromma, Sweden). The two-phase solvent system consisted of *n*-hexane, acetonitrile, and MeOH (40/25/10) [72]. The lower phase was first used as a stationary phase in ascending mode (ASC) for 800 mL; secondly, the process was continued in descending mode (DSC) for another 200 mL (flow rate 5 mL/min, rotation speed 1000 rpm). Two of the following obtained subfractions of HEP8C1–10 were further processed by preparative HPLC: fraction (mode, retention volume, and weight)—HEP8C4 (ASC 476–625 mL, 29.8 mg) and HEP8C6 (ASC 153–160 mL + DSC 15–40 mL, 154.3 mg).

#### 4.3.3. Solid Phase Extraction and Flash Chromatography of the MeOH Fraction

To the MeOH fraction, a solid phase extraction was applied first as follows: fraction M1 (107.0 g) was eluted by ethyl acetate from the silica gel (Geduran Si 60 (0.063–0.200 mm), Merck Chemicals, Darmstadt, Germany) and fraction M2 (42.3 g) with MeOH afterwards. As a second step, a flash chromatography was again applied to M1 using a silica column (100 × 3.6 cm, SI60 0.063–0.2 mm, approx. 520 g, Merck KGaA, Darmstadt, Germany) and a *n*-hexane/ethyl acetate/MeOH gradient. Ten subfractions M1.1–10 were collected, and fractions M1.2 (1150–1575 mL retention volume, 0.6 g weight) and M1.4 (2250–2850 mL, 7.7 g) were further processed.

#### 4.3.4. CPC of Fraction M1.2

M1.2 was subjected to a CPC using the same conditions and two-phase solvent system as described above for HEP8. In contrast, the ASC mode was performed for 925 mL and DSC for another 275 mL. Subfractions M1.2C1–7 were formed, of which the following were processed further: fraction (mode, retention volume, and weight)—M1.2C3 (ASC 311–620 mL, 107.0 mg), M1.2C4 (ASC, 621–925 mL, 70.0 mg), M1.2C6 (DSC 986–1060 mL, 129.7 mg), and M1.2C7 (DSC, 1061–1200 mL, 73.5 mg). By preparative HPLC, M1.2C3 was processed as earlier described [5] and, additionally to the compounds presented there, **36** (18.0 min retention time; 5.6 mg weight), 1(10)*E*,4*E*-furanodienone (21.0 min; 1.9 mg), and 2-acetoxyfuranodiene (25.8 min; 1.5 mg) were gained. Furthermore, **37** was isolated from M1.2C6 and **34** and **35** from M1.2C7, as published before [5], as well as, additionally, 1*R*,2*R*-epoxy-4*S*-furanogermacr-10(14)-en-6-one (10.2 min; 5.2 mg).

#### 4.3.5. Flash Chromatography of M1.4 and the Resulting Fraction M1.4R1F7

A dry sample of M1.4 was placed in a SVP D40 RP18 column (13 × 4 cm, RP18 25–40 µm, 90 g, Merck, Darmstadt, Germany) and separated into seven subfractions M1.4R1–7 by a water (A)/MeOH (B) gradient (0–30 min 40% A/60% B, 20 mL/min; 30–60 min 60–100% B, 20–30 mL/min; 60–90 min 100% B, 30 mL/min). After evaporation and freeze drying, M1.4R1 (90–260 mL retention volume, 1.6 g weight) was further fractionated using a FlashPure EcoFlex silica column (silica 50 µm irregular, 40 g, Büchi Labortechnik, Flawil, Switzerland) and a dichloromethane (A)/ethyl acetate (B)/MeOH (C) gradient (0–60 min 90% A/10% B–60% B, 60–80 min 40% A/60% B–100% B, 80–85 min 100% B, 85–95 min 100% B–100% C, 95–106 min 100% C) and a flow of 15 mL/min. The following four of nine subfractions were further investigated: fraction (retention volume, weight)—M1.4R1F1 (0–150 mL, 169.2 mg), M1.4R1F4 (225–330 mL, 245.0 mg), M1.4R1F6 (480–570 mL, 117.4 mg), and M1.4R1F7 (570–855 mL, 171.4 mg).

While the first three fractions were processed directly by preparative HPLC, fraction M1.4R1F7 was separated on another silica flash column (Geduran Si60 0.063–0.2 mm, 26 g, Merck, Darmstadt, Germany). An *n*-hexane (A)/ethyl acetate (B)/MeOH (C) gradient (0–25 min 60% A/40% B, 25–35 min 60% A/40% B–60% B, 35–45 min 40% A/60% B–100% B, 45–55 min 100% B, 55–65 min 100% B–100% C, 65–70 min 100% C; flow: 10 mL/min) divided it into further four subfractions M1.4R1F7.1–4, of which two were used for compound isolation as follows: fraction (retention volume, weight)—M1.4R1F7.1 (145–430 mL, 43.7 mg) and M1.4R1F7.2 (430–490 mL, 24.4 mg).

#### 4.3.6. Thin-Layer Chromatography (TLC)

TLC analysis of the fractions of CPC and flash chromatographic separations was conducted on silica gel 60 F254 (Merck, Darmstadt, Germany) with mobile phases consisting of toluene/ethyl acetate 95/5 or 80/20 (*v*/*v*) for the HEP fractions and the fractions gained from M1.2, *n*-hexane/ethyl acetate/MeOH 15/80/5 (*v*/*v*/*v*) for the MeOH fractions or *n*-hexane/ethyl acetate/formic acid 2/3/0.1 (*v*/*v*/*v*) for the fractions gained from M1.4. Compounds were derivatized by anisaldehyde reagent *R* (Ph. Eur.) and detected as well as documented by a Camag TLC visualizer (Camag AG, Muttenz, Switzerland).

#### 4.3.7. Preparative HPLC

For final substance isolation, a preparative HPLC device including a 1260 Infinity binary pump, a 1260 Infinity manual injector, a 1260 Infinity fraction collector, a 1260 Infinity diode array detector (all Agilent Technologies, Santa Clara, CA, USA), and a Kinetex^®^ column (Biphenyl, 100 Å, 5 µm, 21.2 × 250 mm, Phenomenex, Aschaffenburg, Germany; 21 mL/min flow rate) were used; only for M1.2C4, a Nucleodur^TM^ C18 Isis column was used (RP18, 5 µm, 10 × 250 mm, Macherey-Nagel, Düren, Germany; 5 mL/min flow rate). The compounds were separated by ACN/water gradients (Table 8) and detected at 200 nm. Afterwards, the ACN was eliminated by evaporation, the aqueous phases partitioned four times with diethyl ether and the organic phases were dried under a nitrogen stream.

### 4.4. Compound Characterisation

The 1D and 2D NMR spectra (^1^H, ^13^C, HSQC, HMBC, COSY and NOESY) were recorded at 298 K in CD_3_OD or CDCl_3_ on an AVANCE III 600 NMR equipped with a 5 mm TBI CryoProbe or an AVANCE III HD 400 NMR (Bruker Corporation, Billerica, MA, USA).

HRESIMS analysis of the isolates was performed with a 1290 Infinity UHPLC as well as a ZORBAX Eclipse column (XDB-C18 RRHD, 2.1 × 100 mm, 1.8 µm; Agilent Technologies, Santa Clara, CA, USA). The solvent system consisted of 0.1% formic acid in water or in ACN, and electrospray ionization (ESI) was carried out in the positive and negative modes. For the triterpenoid and phytosteroid compounds, GC combined with chemical ionisation (APCI) were chosen to achieve their sufficient ionisation. An Agilent 7890A GC system and a (5% phenyl)-methylpolysiloxane phase capillary column Agilent 19091S-433HP-5MS (30 m, 0.25 mm, 0.25 µm, 7-inch cage) were used (Agilent Technologies). For both separation modes, LC and GC, the ionisation and mass spectrometric analysis were conducted using a Q-TOF 6540 UHD mass spectrometer (Agilent Technologies).

Optical properties of all compounds were measured using solutions in MeOH. The specific optical rotation at 589 nm was obtained using an UniPol L 1000 polarimeter (Schmidt + Haensch GmbH & Co., Berlin, Germany). A Cary 50 Scan UV–Vis spectrophotometer (Varian Deutschland GmbH, Darmstadt, Germany) was applied for recording UV–Vis spectra in a range of 200–800 nm. Additionally, CD spectra were measured on a J-715 spectropolarimeter (JASCO Deutschland GmbH, Gross-Umstadt, Germany) with a 0.1 cm quartz cuvette. Thereby, ten scans from 190–300 or 350 nm (scanning rate: 100 nm/min; 0.5 nm steps; 22 °C) were averaged and smoothed by the Savitzky–Golay algorithm (convolution width: 15).

The purity of the isolates was determined by HPLC-DAD analysis on an Elite LaChrom system consisting of an autosampler L-2200, a pump L-2130, a column oven L-2350, a DAD L-2455 (all Hitachi, Tokyo, Japan) and a Kinetex^®^ biphenyl column (100 Å, 5 µm, 4.6 × 250 mm, Phenomenex, Aschaffenburg, Germany), or a Nucleodur^TM^ C18 Isis column (RP18, 5 µm, 4.6 × 250 mm, Macherey-Nagel, Düren, Germany), respectively. The respective gradients used for isolation (Table 8) were conducted at a flow rate of 1 mL/min. The purity was calculated as the proportion of the integral of the main peak in the chromatogram in the maxplot (190–400 nm for the HEP fraction or 200–400 nm for the MeOH fraction) using the software EZChrom Elite 3.1.7 (Hitachi, Tokyo, Japan).

### 4.5. Isolated Compounds

The following chemical structures were drawn in ChemDrawProfessional 20.0, and three-dimensional models were generated with Chem3D Ultra 20.0 (both PerkinElmer Informatics Inc., Waltham, MA, USA).

*1β,5,8-Trihydroxy-4α,8-cycloeudesma-2,7(11)*Z*-dien-12-al* (**1**): 1.4 mg, colourless oil; ${\left[\alpha \right]}_{D}^{25}$ −226 (*c* 1.17, MeOH); UV (MeOH) λ_max_ (log *ε*): 257 nm (3.92); CD: Figure S24; ^1^H and ^13^C NMR data: Table 1, original spectra: Figure S1, HSQC and HMBC: Figure S2, COSY and NOESY: Figure S3; HRESIMS *m*/*z* 265.1435 [M+H]^+^ (calc. for C_15_H_21_O_4_, 265.1434); purity: 92.1%.

*2β-Acetyloxy-6β-hydroxyglechomanolide* (**2**): 2.5 mg, colourless oil; ${\left[\alpha \right]}_{D}^{22}$ −15 (*c* 2.51, MeOH); UV (MeOH) λ_max_ (log *ε*): 208 nm (4.06); CD: Figure S24; ^1^H and ^13^C NMR data: Table 1, original spectra: Figure S4; HRESIMS *m*/*z* 307.1545 [M+H]^+^ (calc. for C_17_H_23_O_5_, 307.1540); purity: 87.6%.

*4β,5α-Epoxyglechomanolide* (**3**): in mixture with **4** and **22**: 13.1 mg, yellowish oil; ^1^H and ^13^C NMR data: Table 1, original spectra: Figure S5; HRESIMS *m*/*z* 249.1485 [M+H]^+^ (calc. for C_15_H_21_O_3_, 249.1491).

*2β-Acetyloxy-4β,5α-epoxyglechomanolide* (**4**): in mixture with **3** and **22**: 13.1 mg, yellowish oil; ^1^H and ^13^C NMR data: Table 1, original spectra: Figure S6; HRESIMS *m*/*z* 307.1543 [M+H]^+^ (calc. for C_17_H_23_O_5_, 307.1545).

*2β-Acetyloxy-4β,5α-epoxy-8-*epi*-hydroxyglechomanolide* (**5**): 5.2 mg, white solid; ${\left[\alpha \right]}_{D}^{23}$ +17 (*c* 2.61, MeOH); UV (MeOH) λ_max_ (log *ε*): no maximum detected; CD: Figure S24; ^1^H and ^13^C NMR data: Table 2, original spectra: Figure S7; HRESIMS *m*/*z* 323.1492 [M+H]^+^ (calc. for C_17_H_23_O_6_, 323.1489); purity: 88.7%.

*8-*epi*-Serralactone A* (**6**): 4.3 mg, colourless oil; ${\left[\alpha \right]}_{D}^{23}$ −2.0 (*c* 2.13, MeOH); UV (MeOH) λ_max_ (log *ε*): 219 nm (4.10); CD: Figure S24; ^1^H and ^13^C NMR data: Table 2, original spectra: Figure S8; HRESIMS *m*/*z* 249.1491 [M+H]^+^ (calc. for C_15_H_21_O_3_, 249.1485); purity: 93.2%.

*1α,8β-Dihydroxy-2β-ethyloxyeudesma-3,7(11)-dien-8α,12-olide* (**7**): 2.6 mg, colourless oil; ${\left[\alpha \right]}_{D}^{23}$ +5.8 (*c* 2.36, MeOH); UV (MeOH) λ_max_ (log *ε*): no maximum detected; CD: Figure S24; ^1^H and ^13^C NMR data: Table 2, original spectra: Figure S9; HRESIMS *m*/*z* 309.1698 [M+H]^+^ (calc. for C_17_H_25_O_5_, 309.1697); purity: 81.9%.

*8-*epi*-Neolitacumone B* (**8**): 1.8 mg, yellowish oil; ${\left[\alpha \right]}_{D}^{22}$ +43 (*c* 1.81, MeOH); UV (MeOH) λ_max_ (log *ε*): 221 nm (3.99), 276 nm (3.34); CD: Figure S24; ^1^H and ^13^C NMR data: Table 3, original spectra: Figure S10; HRESIMS *m*/*z* 249.1490 [M+H]^+^ (calc. for C_15_H_21_O_3_, 249.1491); purity: 76.7%.

*2α-Acetyloxyneolitacumone A* (**9**): 1.6 mg, white solid; ${\left[\alpha \right]}_{D}^{25}$ −89 (*c* 1.57, MeOH); UV (MeOH) λ_max_ (log *ε*): 214 nm (3.96); CD: Figure S24; ^1^H and ^13^C NMR data: Table 3, original spectra: Figure S11; HRESIMS *m*/*z* 321.1344 [M–H]^–^ (calc. for C_17_H_21_O_6_, 321.1344); purity: 84.0%.

*3-Oxo-8α*H*-eudesma-1,4,7(11)-trien-8,12-olide* (**10**): 2.5 mg, yellowish oil; ${\left[\alpha \right]}_{D}^{23}$ −37 (*c* 2.05, MeOH); UV (MeOH) λ_max_ (log *ε*): 221 nm (3.87); CD: Figure S24; ^1^H and ^13^C NMR data: Table 3, original spectra: Figure S12; HRESIMS *m*/*z* 245.1174 [M+H]^+^ (calc. for C_15_H_17_O_3_, 245.1172); purity: 93.0%.

*3-Oxo-8β-hydroxy-5α*H*-eudesma-1,4(15),7(11)-trien-8,12-olide* (**11**): 2.1 mg, colourless oil; ${\left[\alpha \right]}_{D}^{24}$ −18 (*c* 2.08, MeOH); UV (MeOH) λ_max_ (log *ε*): 220 nm (4.06); CD: Figure S24; ^1^H and ^13^C NMR data: Table 3, original spectra: Figure S13; HRESIMS *m*/*z* 261.1126 [M+H]^+^ (calc. for C_15_H_17_O_4_, 261.1121); purity: 86.2%.

*4-*epi*-Linderolide J* (**12**): 1.8 mg, yellowish oil; ${\left[\alpha \right]}_{D}^{23}$ +71 (*c* 1.82, MeOH); UV (MeOH) λ_max_ (log *ε*): 221 nm (3.88); CD: Figure S24; ^1^H and ^13^C NMR data: Table 4, original spectra: Figure S14; HRESIMS *m*/*z* 277.1067 [M+H]^+^ (calc. for C_15_H_17_O_5_, 277.1071); purity: 87.4%.

*1β,8β-Dihydroxy-5α*H*-eudesma-2,4(15),7(11)-trien-8,12-olide* (**13**): 1.8 mg, white solid; ${\left[\alpha \right]}_{D}^{23}$ +33 (*c* 1.78, MeOH); UV (MeOH) λ_max_ (log *ε*): 223 nm (3.93); CD: Figure S24; ^1^H and ^13^C NMR data: Table 4, original spectra: Figure S15; HRESIMS *m*/*z* 263.1277 [M+H]^+^ (calc. for C_15_H_19_O_4_, 263.1278); purity: 95.5%.

*1α,5α,8β-Trihydroxyeudesma-2,4(15),7(11)-trien-8,12-olide* (**14**): 0.8 mg, white solid; ${\left[\alpha \right]}_{D}^{25}$ +146 (*c* 0.87, MeOH); UV (MeOH) λ_max_ (log *ε*): 223 nm (3.98); CD: Figure S24; ^1^H and ^13^C NMR data: Table 4, original spectra: Figure S16; HRESIMS *m*/*z* 279.1226 [M+H]^+^ (calc. for C_15_H_19_O_5_, 279.1227); purity: 93.0%.

*1α,5α-Dihydroxyeudesma-2,4(15),7(11),8-tetraen-8,12-olide* (**15**): 5.0 mg, yellowish oil; ${\left[\alpha \right]}_{D}^{24}$ +168 (*c* 2.49, MeOH); UV (MeOH) λ_max_ (log *ε*): 224 nm (4.02), 276 nm (3.96); CD: Figure S24; ^1^H and ^13^C NMR data: Table 5, original spectra: Figure S17; HRESIMS *m*/*z* 261.1125 [M+H]^+^ (calc. for C_15_H_17_O_4_, 261.1121); purity: 87.3%.

*1,2-Anhydro-8-oxo-tussfarfarin A* (**16**): 4.7 mg, yellowish oil; ${\left[\alpha \right]}_{D}^{23}$ +75 (*c* 2.04, MeOH); UV (MeOH) λ_max_ (log *ε*): 318 nm (3.81); CD: Figure S24; ^1^H and ^13^C NMR data: Table 5, original spectra: Figure S18; HRESIMS *m*/*z* 231.1017 [M+H]^+^ (calc. for C_14_H_15_O_3_, 231.1016); purity: 98.3%.

*8,14-Dihydroxy-9,10-seco-isolindestrenolide* (**17**): 1.0 mg, white solid; ${\left[\alpha \right]}_{D}^{23}$ −9.5 (*c* 0.99, MeOH); UV (MeOH) λ_max_ (log *ε*): shoulder at 275 nm; CD: Figure S24; ^1^H and ^13^C NMR data: Table 5, original spectra: Figure S19; HRESIMS *m*/*z* 263.1274 [M+H]^+^ (calc. for C_15_H_19_O_4_, 263.1278); purity: 94.0%.

*Serralactone A* (**18**): in mixture with **20**: 6.6 mg, white solid; ^1^H and ^13^C NMR data: comparable to [26]; HRESIMS *m*/*z* 249.1491 [M+H]^+^ (calc. for C_15_H_21_O_3_, 249.1491).

*1β,8β-Dihydroxyeudesman-3,7(11)-dien-8α,12-olide* (**19**): 5.0 mg, white solid; ${\left[\alpha \right]}_{D}^{25}$ −9.7 (*c* 2.19, MeOH); UV (MeOH) λ_max_ (log *ε*): 214 nm (3.99); CD: Figure S25; ^1^H and ^13^C NMR data: comparable to [27]; HRESIMS *m*/*z* 265.1437 [M+H]^+^ (calc. for C_15_H_21_O_4_, 265.1434); purity: 82.5%.

*Neolitacumone B* (**20**): in mixture with **18**: 6.6 mg, white solid; ^1^H and ^13^C NMR data: comparable to [28]; HRESIMS *m*/*z* 249.1491 [M+H]^+^ (calc. for C_15_H_21_O_3_, 249.1491).

*(–)-Neolitacumone A* ((–)-**21**): 3.6 mg, white solid; ${\left[\alpha \right]}_{D}^{25}$ −7.3 (*c* 2.02, MeOH); UV (MeOH) λ_max_ (log *ε*): 220 nm (3.96); CD: Figure S25; ^1^H and ^13^C NMR data: comparable to [28]; HRESIMS *m*/*z* 265.1438 [M+H]^+^ (calc. for C_15_H_21_O_4_, 265.1434); purity: 94.1%.

*3-Oxo-5α*H*,8β*H*-eudesma-1,4(15),7(11)-trien-8,12-olide* (**22**): in mixture with **3** and **4**: 13.1 mg, yellowish oil; ^1^H and ^13^C NMR data: comparable to [29]; HRESIMS *m*/*z* 245.1176 [M+H]^+^ (calc. for C_15_H_17_O_3_, 245.1172).

*(+)-Eudebeiolide B* (**23**): 1.6 mg, colourless oil; ${\left[\alpha \right]}_{D}^{24}$ +46 (*c* 1.58, MeOH); UV (MeOH) λ_max_ (log *ε*): 220 nm (4.03); CD: Figure S25, determination of the absolute configuration in comparison to [30]; ^1^H and ^13^C NMR data: comparable to [30]; HRESIMS *m*/*z* 263.1280 [M+H]^+^ (calc. for C_15_H_19_O_4_, 263,1278); purity: 70.2%.

*Linderolide I* (**24**): 2.7 mg, yellowish oil; ${\left[\alpha \right]}_{D}^{23}$ +82 (*c* 2.72, MeOH); UV (MeOH) λ_max_ (log *ε*): 221 nm (4.07), shoulder at 275 nm; CD: Figure S25; ^1^H and ^13^C NMR data: Table 5; HRESIMS *m*/*z* 263.1281 [M+H]^+^ (calc. for C_15_H_19_O_4_, 263.1278); purity: 92.3%.

*1β,8β-Dihydroxyeudesma-4,7(11)-dien-8α,12-olide* (**25**): 1.8 mg, white solid; ${\left[\alpha \right]}_{D}^{25}$ −19 (*c* 0.92, MeOH); UV (MeOH) λ_max_ (log *ε*): 218 nm (4.00); CD: Figure S25; ^1^H and ^13^C NMR data: comparable to [73]; HRESIMS *m*/*z* 265.1438 [M+H]^+^ (calc. for C_15_H_21_O_4_, 265.1434); purity: 78.6%.

*Istanbulin B* (**26**): 6.4 mg, yellowish oil; ${\left[\alpha \right]}_{D}^{23}$ −5.4 (*c* 2.12, MeOH); UV (MeOH) λ_max_ (log *ε*): 220 nm (4.14); CD: Figure S25; ^1^H and ^13^C NMR data: comparable to [74]; HRESIMS *m*/*z* 249.1490 [M+H]^+^ (calc. for C_15_H_21_O_3_, 249.1491); purity: 94.8%.

*Istanbulin A* (**27**): 4.0 mg, white solid; ${\left[\alpha \right]}_{D}^{24}$ +23 (*c* 2.47, MeOH); UV (MeOH) λ_max_ (log *ε*): 219 nm (3.93); CD: Figure S25; ^1^H and ^13^C NMR data: comparable to [74]; HRESIMS *m*/*z* 265.1436 [M+H]^+^ (calc. for C_15_H_21_O_4_, 265.1434); purity: 70.3%.

*Chloraniolide A* (**28**): 2.3 mg, yellowish oil; ${\left[\alpha \right]}_{D}^{25}$ −4.2 (*c* 2.33, MeOH); UV (MeOH) λ_max_ (log *ε*): shoulder at 218 nm; CD: Figure S25; ^1^H and ^13^C NMR data: comparable to [34]; HRESIMS *m*/*z* 267.1595 [M+H]^+^ (calc. for C_15_H_23_O_4_, 267.1591); purity: 65.2%.

*2*S*-Methoxy-4*S*-furanogermacra-1(10)*E*-en-6-one* (**29**): 11.6 mg, white solid; ${\left[\alpha \right]}_{D}^{22}$ −89 (*c* 2.58, MeOH); UV (MeOH) λ_max_ (log *ε*): 206 nm (3.98); CD: Figure S25, determination of the absolute configuration in comparison to [38]; ^1^H and ^13^C NMR data: comparable to [37]; HRESIMS *m*/*z* 263.1640 [M]^+^ (calc. for C_16_H_23_O_3_, 263.1642) purity: 91.2%.

The physicochemical properties of compounds **30**, **31**, **34**, **35** and **37** [5] as well as **32** and **33** [21] were published before.

*Alismol* (**36**): 5.6 mg, colourless oil; ${\left[\alpha \right]}_{D}^{22}$ −0.8 (*c* 2.1, MeOH); UV (MeOH) λ_max_ (log *ε*): no maximum detected; CD: Figure S25; ^1^H and ^13^C NMR data: comparable to [75]; HRESIMS *m*/*z* 220.1816 [M]^+^ (calc. for C_15_H_24_O; 220.1827); purity: 90.5%.

*29-Norlanost-8,24-dien-1α,3β-diol* (**38**): 2.4 mg, white solid; ${\left[\alpha \right]}_{D}^{22}$ +93 (*c* 2.40, MeOH); UV (MeOH) λ_max_ (log *ε*): 203 nm (4.10); CD: Figure S25; ^1^H and ^13^C NMR data: Table 6, original spectra: Figure S20; GC-APCI *m*/*z* 429.3727 [M+H]^+^ (calc. for C_29_H_49_O_2_, 429.3727); purity: 84.8%.

*11α-Hydroxysitost-4-en-3-one* (**39**): 0.8 mg, colourless oil; ${\left[\alpha \right]}_{D}^{23}$ +40 (*c* 0.89, MeOH); UV (MeOH) λ_max_ (log *ε*): 203 nm (3.96); CD: Figure S25; ^1^H and ^13^C NMR data: Table 6, original spectra: Figure S21; GC-APCI *m*/*z* 429.3728 [M+H]^+^ (calc. for C_29_H_49_O_2_, 429.3727); purity: 83.5%.

*11α-Hydroxystigmast-4-en-3-one* (**40**): 0.4 mg, white solid; ${\left[\alpha \right]}_{D}^{22}$ +39 (*c* 0.44, MeOH); UV (MeOH) λ_max_ (log *ε*): 243 nm (4.36); CD: Figure S25; ^1^H and ^13^C NMR data: Table 6, original spectra: Figure S22; GC-APCI *m*/*z* 427.3570 [M+H]^+^ (calc. for C_29_H_47_O_2_, 427.3571); purity: 87.5%.

*Stigmasta-5,22*E*-diene-3β,11α-diol* (**41**): 4.7 mg, yellowish solid; ${\left[\alpha \right]}_{D}^{23}$ −2.9 (*c* 2.35, MeOH); UV (MeOH) λ_max_ (log *ε*): 201 nm (3.96); CD: Figure S25; ^1^H and ^13^C NMR data comparable to [36]; GC-APCI *m*/*z* 446.3988 [M+NH_4_]^+^ (calc. for C_29_H_49_NO_2_, 446.3993); purity: 45.1%.

*7-Ketostigmasterol* (**42**): 0.5 mg, white solid; ${\left[\alpha \right]}_{D}^{22}$ −21 (*c* 0.56, MeOH); UV (MeOH) λ_max_ (log *ε*): 238 nm (4.03); CD: Figure S26; ^1^H and ^13^C NMR data: comparable to [76]; GC-APCI *m*/*z* 427.3572 [M+H]^+^ (calc. for C_29_H_47_O_2_, 427.3571); purity: 84.1%.

rel*-7′-(3′-Methoxy-4′,5′-methylendioxyphenyl)-3,4,5-trimethoxy-7,8*S*,7′*R*,8′*R*-tetrahydronaphtho[2,3-c]furan-9′(3H)-one* (**43**): 1.3 mg, yellowish solid; ${\left[\alpha \right]}_{D}^{24}$ −1.7 (*c* 1.26, MeOH); UV (MeOH) λ_max_ (log *ε*): shoulder at approx. 280 nm; CD: Figure S26; ^1^H and ^13^C NMR data: Table 7, original spectra: Figure S23; HRESIMS *m*/*z* 429.1547 [M+H]^+^ (calc. for C_23_H_25_O_8_, 429.1544); purity: 42.3%.

### 4.6. RAW 264.7 Experiments

The RAW 264.7 mouse macrophages cell line was purchased from CLS (Eppelheim, Germany). The cells were cultured in RPMI 1640 medium with phenol red, supplemented with 10% heat inactivated FBS and 1% L-glutamine, as well as in an atmosphere of 5% CO_2_ and 90% relative humidity at 37 °C. Mycoplasma contamination was excluded by PCR and culture from GATC Biotech AG (Konstanz, Germany).

#### 4.6.1. MTT Assay

This assay was performed similarly as previously described [5,77]. Cells from a confluent culture flask were seeded into 96-well plates in RPMI medium without phenol red (10,000 cells/well in 100 µL) and incubated for 24 h. The medium was then removed, a sample solution in medium was added (5–100 µM, max. 0.14% DMSO, *v*/*v*), and the cells were incubated for a further 24 h. Subsequently, the supernatant was replaced with 100 µL of an MTT solution in medium (0.4 mg/mL) and incubated for three hours. The cells were then treated with 10% SDS in water and stored at room temperature in the dark. By the next day, the formazan crystals had dissolved and the absorbance at 560 nm could be determined using a Tecan microplate reader (Tecan Trading AG, Maennedorf, Switzerland). Cell viability was calculated as a proportion compared to the average absorbance of the negative control (medium only). To exclude the possibility of solvent effects, some cells were also treated with the highest DMSO concentration used. All assays were performed three times independently in hexaplicates.

#### 4.6.2. Griess Assay

Cells from a confluent culture flask were seeded into 96-well plates in RPMI medium without phenol red (100,000 cells/well in 100 µL) and incubated for 24 h. The medium was then removed, a sample solution in medium containing 1 µg/mL LPS was added (1–70 µM, max. 0.1% DMSO, *v*/*v*), and the cells were incubated for a further 24 h. A total of 70 µL of the supernatants were then mixed with the same volume of Griess reagent under exclusion of light and, after 15 min, the absorbance at 560 nm was measured using a Tecan microplate reader (Tecan Trading AG, Maennedorf, Switzerland). Nitrite concentrations were determined by an external calibration with sodium nitrite, and their proportions to the negative control were calculated. Parthenolide (5 µM) was used as a positive control, medium without LPS as an untreated control, medium with LPS as a negative control, and the highest DMSO concentration used as a solvent control. All assays were performed three times independently in hexaplicates.

#### 4.6.3. Statistics

Significance levels were calculated in two-sided Student’s *t*-tests using Microsoft Excel 2305 (Microsoft Corporation, Redmond, WA, USA). The diagrams were made by GraphPad Prism 5.00 (GraphPad Software, Boston, MA, USA), which was also used to determine the nonlinear regression curve.

## Figures and Tables

**Table 1:** ^1^H and ^13^C NMR data of compounds **1**–**4** (**1**, **2**: 600 and 151 MHz; **3**, **4**: 400 and 101 MHz) in CD_3_OD (*δ* in ppm, *J* in Hz, s: singlet, d: doublet, t: triplet, q: quartet, br: broad, and m: multiplet).

No.	1		2		3		4	
	*δ*_H_	*δ*_C_	*δ*_H_	*δ*_C_	*δ*_H_	*δ*_C_	*δ*_H_	*δ*_C_
1	3.61 (1H, d, 4.5)	76.6	5.00 (1H, d, 10.3)	132.2	4.87 (1H, brd, 11.6)	129.3	5.35 (1H, d, 10.4)	128.4
2	5.97 (1H, dd, 4.5, 9.8)	130.5	5.40 (1H, ddd, 5.0, 10.5, 10.5)	72.0	2.08 ^a^ (1H, m) 2.36 ^a^ (1H, m)	24.6	5.54 (1H, ddd, 5.0, 10.6, 11.2)	69.0
3	5.45 (1H, d, 9.7)	133.2	2.15 (1H, dd, 11.0, 11.0) 2.48 (1H, dd, 4.9, 11.3)	45.4	1.14 ^a^ (1H, m) 2.01 ^a^ (1H, m)	37.3	1.28 ^a^ (1H, m) 2.43 ^a^ (1H, m)	42.6
4		55.5		134.7		60.1		59.4
5		79.6	5.20 (1H, d, 7.3)	134.7	2.94 ^a^ (1H, m)	63.2	3.16 (1H, dd, 3.9, 8.4)	61.5
6	2.43 (1H, d, 18.7) 2.75 (1H, d, 18.7)	38.2	5.53 (1H, d, 7.3)	65.8	2.22 (1H, dd, 11.9, 14.9) 2.95 ^a^ (1H, m)	22.0	2.56 ^a^ (1H, m) 3.01 ^a^ (1H, m)	26.6
7		165.4		163.9		162.5		159.5
8		88.6	5.42 ^a^ (1H, m)	83.4	5.11 (1H, d, 6.0)	83.1	5.39 (1H, dd, 5.4, 6.2)	83.5
9	1.54 (1H, d, 12.5) 1.68 ^a^ (1H, d, 12.7)	47.8	2.79 (1H, dd, 6.1, 14.9) 2.91 (1H, dd, 2.3, 14.8)	42.7	2.54 ^a^ (1H, m) 2.64 ^a^ (1H, m)	38.6	2.40 ^a^ (1H, m) 3.00 ^a^ (1H, m)	40.5
10		43.2		137.1		127.4		133.5
11		131.3		127.5		124.4		126.5
12	10.68 (1H, s)	195.7		175.8		174.4		174.2
13	1.67 ^a^ (3H, s)	11.1	1.94 (3H, d, 1.7)	9.7	2.17 (3H, s)	12.4	1.80 (3H, brs)	8.0
14	1.20 (3H, s)	18.9	1.36 (3H, s)	17.1	1.67 (3H, brs)	18.6	1.69 (3H, brs)	17.9
15	1.02 (3H, s)	15.0	1.59 (3H, s)	18.7	1.16 (3H, s)	14.8	1.23 (3H, s)	16.8
16				172.4				170.6
17			2.01 (3H, s)	21.0			1.99 (3H, s)	19.5

^a^ overlapped signal.

**Table 2:** ^1^H and ^13^C NMR data of compounds **5**–**7** (**5**, **6**: 400 and 101 MHz, **7**: 600 and 151 MHz) in CD_3_OD (*δ* in ppm, *J* in Hz, s: singlet, d: doublet, t: triplet, q: quartet, br: broad, and m: multiplet).

No.	5		6		7	
	*δ*_H_	*δ*_C_	*δ*_H_	*δ*_C_	*δ*_H_	*δ*_C_
1	5.21 (1H, d, 9.7)	129.5	3.58 (1H, dd, 6.0, 10.4)	74.8	3.44 (1H, s)	74.9
2	5.64 (1H, m)	69.1	1.94–1.88 (1H, m) 2.20–2.12 (1H, m)	31.3	3.58 (1H, m)	80.6
3	1.27 (1H, dd, 10.7, 10.7) 2.46 (1H, dd, 5.2, 11.7)	41.7	5.36 (1H, dd, 1.0, 2.3)	120.5	5.50 (1H, m)	121.9
4		59.7		133.3		137.7
5	2.74 ^a^ (1H, m)	63.7	2.60 (1H, m)	40.8	2.26 ^a^ (1H, m)	42.9
6	2.35 (1H, dd, 8.8, 14.6) 2.74 ^a^ (1H, m)	22.8	2.49 (1H, dd, 12.9, 17.7) 2.86 (1H, ddq, 1.9, 3.7, 17.7)	25.0	2.31 (1H, dd, 12.5, 12.5) 2.96 (1H, dd, 2.8, 12.2)	24.6
7		159.0		164.4		162.9
8		106.0	5.20 (1H, ddq, 1.9, 8.0, 8.0)	78.3		106.3
9	2.40 (1H, d, 13.4) 2.97 (1H, d, 13.3)	50.4	1.40 (1H, dd, 6.6, 14.1) 2.43 (1H, dd, 10.9, 14.0)	40.5	1.96 (1H, d, 13.2) 2.01 (1H, d, 11.5)	46.3
10		133.8		38.0		38.5
11		129.5		120.2		122.8
12		171.5		176.0		174.5
13	1.89 (3H, s)	7.5	1.79 (3H, brs)	6.8	1.82 (3H, s)	8.0
14	1.99 (3H, s)	17.6	0.67 (3H, s)	12.8	1.11 (3H, s)	16.6
15	1.30 (3H, s)	16.9	1.70 (3H, s)	19.2	1.80 (3H, s)	21.4
16		170.6			3.55 (1H, dq, 7.1, 9.4) 3.65 (1H, dq, 7.1, 9.2)	65.8
17	1.99 (3H, s)	19.5			1.17 (3H, dd, 6.9, 6.9)	15.9

^a^ overlapped signal.

**Table 3:** ^1^H and ^13^C NMR data of compounds **8**–**11** (600 and 151 MHz, respectively) in CD_3_OD (*δ* in ppm, *J* in Hz, s: singlet, d: doublet, t: triplet, q: quartet, br: broad, and m: multiplet).

No.	8		9		10		11	
	*δ*_H_	*δ*_C_	*δ*_H_	*δ*_C_	*δ*_H_	*δ*_C_	*δ*_H_	*δ*_C_
1	3.60 (1H, dd, 4.3, 11.6)	79.1	3.29 ^a^ (1H, m)	80.9	6.97 (1H, d, 9.9)	157.3	6.99 (1H, d, 10.0)	162.3
2	1.47 ^a^ (1H, m) 1.78 ^a^ (1H, m)	32.3	4.80 (1H, ddd, 6.0, 10.5, 11.9)	74.9	6.18 (1H, d, 9.9)	125.8	5.96 (1H, d, 9.9)	127.1
3	2.17 ^a^ (1H, m) 2.37 (1H, ddd, 2.4, 4.4, 13.7)	35.0	2.10 (1H, dd, 12.0, 12.0) 2.71 (1H, dd, 5.5, 12.8)	40.3		187.3		190.2
4		148.1		145.6		133.9		146.1
5	2.52 (1H, dd, 5.5, 12.7)	42.0	2.02 ^a^ (1H, m)	50.4		156.6	2.69 (1H, dddd, 2.4, 2.4, 2.4, 12.6)	50.7
6	2.69 (1H, ddq, 1.7, 5.1, 17.1) 2.81 (1H, dd, 12.7, 18.8)	25.5	2.48 (1H, dd, 12.5, 12.5) 2.73 (1H, dd, 4.2, 13.3)	24.7	3.79 (1H, s) 3.81 (1H, s)	29.1	2.56 (1H, dd, 13.5, 13.5) 2.96 (1H, dd, 2.9, 13.2)	24.6
7		165.0		162.2		162.2		160.6
8	5.19 (1H, ddq, 1.7, 9.3, 9.3)	79.6		105.4	4.93 (1H, m)	78.0		105.0
9	1.20 (1H, dd, 9.1, 13.9) 2.60 (1H, dd, 9.9, 13.9)	42.6	1.45 (1H, d, 13.7) 2.66 (1H, d, 13.6)	48.5	1.90 ^a^ (1H, m) 2.40 (1H, dd, 10.7, 14.0)	40.1	1.77 (1H, d, 13.3) 2.43 (1H, d, 13.4)	47.7
10		41.4		41.9		40.6		39.7
11		121.5		123.0		122.9		124.0
12		177.5		174.4		176.7		174.0
13	1.81 (3H, brs)	8.2	1.80 (3H, brs)	8.1	1.90 (3H, brs)	8.3	1.82 (3H, brs)	8.2
14	0.64 (3H, s)	15.2	1.03 (3H, s)	12.1	1.19 (3H, s)	27.9	1.24 (3H, s)	19.7
15	4.75 (1H, brs) 4.93 (1H, brs)	109.3	4.82 ^a^ (1H, s) 5.00 (1H, s)	110.7	1.94 (3H, s)	11.0	5.45 (1H, d, 2.1) 6.13 (1H, d, 1.7)	119.8
16				172.7				
17			2.04 (3H, s)	21.1				

^a^ overlapped signal.

**Table 4:** ^1^H and ^13^C NMR data of compounds **12**–**14** (600 and 151 MHz, respectively) in CD_3_OD (*δ* in ppm, *J* in Hz, s: singlet, d: doublet, t: triplet, q: quartet, br: broad, and m: multiplet).

No.	12		13		14	
	*δ*_H_	*δ*_C_	*δ*_H_	*δ*_C_	*δ*_H_	*δ*_C_
1	7.14 (1H, d, 10.1)	163.2	4.00 (1H, s)	78.8	3.58 (1H, d, 5.3)	74.5
2	6.03 (1H, d, 10.0)	127.1	5.53 (1H, d, 9.9)	132.8	5.88 (1H, dd, 5.2, 10.1)	128.9
3		195.3	6.17 (1H, dd, 9.9, 2.8)	131.3	6.19 (1H, d, 9.8)	129.6
4		58.3		145.7		147.8
5	2.57 (1H, dd, 3.5, 13.8)	46.4	2.17 ^a^ (1H, m)	47.3		78.2
6	2.37 (1H, dd, 13.0, 13.0) 2.57 (1H, dd, 3.5, 13.8)	21.6	2.41 (1H, dd, 13.8, 13.8) 2.92 (1H, dd, 3.8, 13.2)	24.8	2.68 (1H, brd, 13.8) 3.06 (1H, d, 13.6)	32.1
7		160.6		162.0		160.2
8		104.8		105.4		106.2
9	1.77 (1H, d, 13.3) 2.41 (1H, d, 13.9)	48.0	1.48 (1H, d, 13.7) 2.66 (1H, d, 13.6)	49.0	1.93 (1H, d, 13.7) 2.57 (1H, d, 13.7)	41.5
10		38.8		41.6		41.8
11		124.1		122.9		125.8
12		173.9		174.4		174.6
13	1.81 (3H, brs)	8.1	1.81 (3H, brs)	8.1	1.81 (3H, brs)	8.2
14	1.45 (3H, s)	22.7	0.95 (3H, s)	11.9	1.07 (3H, s)	22.0
15	3.09 (1H, d, 5.5) 3.28 (1H, d, 5.6)	46.9	4.99 (1H, s) 5.03 (1H, s)	112.1	5.16 (1H, s) 5.33 (1H, s)	114.3

^a^ overlapped signal.

**Table 5:** ^1^H and ^13^C NMR data of compounds **15**–**17** and **24** (**17**: 600 and 151 MHz; **15**, **16** and **24**: 400 and 101 MHz) in CD_3_OD (**15**–**17**) or CDCl_3_ (**24**) (*δ* in ppm, *J* in Hz, s: singlet, d: doublet, t: triplet, q: quartet, br: broad, and m: multiplet).

No.	15		16		17		24	
	*δ*_H_	*δ*_C_	*δ*_H_	*δ*_C_	*δ*_H_	*δ*_C_	*δ*_H_	*δ*_C_
1	3.93 (1H, d, 5.3)	72.5	7.08 (1H, d, 9.9)	155.6	7.26 (1H, d, 7.7)	127.5	6.81 (1H, d, 9.9)	158.6
2	5.91 (1H, dd, 5.3, 9.9)	126.9	6.34 (1H, d, 9.9)	126.4	7.19 (1H, dd, 7.4, 7.4)	128.3	5.91 (1H, d, 10.1)	126.0
3	6.25 (1H, d, 9.9)	128.7		185.6	7.15 (1H, d, 7.1)	130.8		201.6
4		145.6		139.6		139.1	2.62 ^a^ (1H, m)	44.2
5		75.2		149.0		134.1	2.11 (1H, dd, 3.0, 6.0, 13.3)	46.4
6	2.93 (1H, dq, 1.8, 17.3) 3.16 (1H, d, 17.2)	29.3	8.24 (1H, s)	143.5	3.87 (2H, brs)	27.6	2.50 (1H, dd, 3.0, 13.5) 2.68 ^a^ (1H, m)	25.5
7		146.6		137.1		161.0		159.3
8		147.9		194.8		107.6		103.0
9	5.76 (1H, s)	112.1	2.68 (2H, d, 5.7)	48.5	1.48 (3H, s)	24.3	1.70 (1H, d, 13.6) 2.34 (1H, d, 13.4)	49.6
10		43.5		41.2		141.3		36.9
11		121.8		197.7		124.8		122.7
12		171.8	2.49 (3H, s)	29.3		174.3		171.1
13	1.91 (3H, brs)	6.9	1.32 (3H, s)	25.9	1.21 (3H, s)	7.7	1.84 (3H, brs)	8.4
14	0.99 (3H, s)	22.2	2.15 (3H, s)	10.0	4.63 (2H, s)	63.7	1.46 (3H, s)	22.5
15	5.22 (1H, s) 5.39 (1H, brs)	113.6			2.29 (3H, s)	20.0	1.27 (3H, d, 8.2)	13.4

^a^ overlapped signal.

**Table 6:** ^1^H and ^13^C NMR data of compounds **38**–**40** (600 and 151 MHz, respectively) in CDCl_3_ (*δ* in ppm, *J* in Hz, s: singlet, d: doublet, t: triplet, q: quartet, br: broad, and m: multiplet).

No.	38		39		40	
	*δ*_H_	*δ*_C_	*δ*_H_	*δ*_C_	*δ*_H_	*δ*_C_
1	3.97 (1H, dd, 3.0, 3.0)	72.7	1.98 ^a^ (1H, m) 2.67 (1H, ddd, 4.5, 4.5, 14.2)	37.6	2.00 (1H, ddd, 4.4, 13.8, 13.8) 2.67 (1H, ddd, 4.5, 4.5, 14.2)	37.6
2	1.67 (1H, ddd, 2.6, 11.1, 13.6) 2.08 ^a^ (1H, m)	36.3	2.32 (1H, ddd, 4.5, 4.5, 17.2) 2.44 (1H, ddd, 4.8, 14.2, 17.2)	34.2	2.33 (1H, ddd, 4.3, 4.3, 17.2) 2.45 (1H, ddd, 4.7, 13.5, 17.3)	34.2
3	3.55 (1H, ddd, 5.1, 9.5, 11.4)	72.3		200.2		200.2
4	1.44 ^a^ (1H, m)	38.6	5.73 (1H, s)	124.5	5.73 (1H, s)	124.5
5	1.45 ^a^ (1H, m)	40.1		171.3		171.2
6	1.32 ^a^ (1H, m) 1.80 ^a^ (1H, m)	19.9	2.27 ^a^ (1H, m) 2.36 (1H, ddd, 5.1, 14.2, 14.2)	33.7	2.27 ^a^ (1H, m) 2.37 (1H, ddd, 5.0, 14.3, 14.3)	33.7
7	2.15 (2H, m)	21.4	1.06 ^a^ (1H, m) 1.83 (1H, m)	31.6	1.05 ^a^ (1H, m) 1.83 (1H, dddd, 2.8, 2.8, 5.2, 12.9)	31.6
8		141.3	1.51 ^a^ (1H, dd, 12.9, 12.9)	34.9	1.52 ^a^ (1H, m)	35.0
9		129.2	1.08 (1H, dd, 10.8, 10.8)	59.3	1.08 ^a^ (1H, m)	59.3
10		42.2		39.9		39.9
11	2.06 (2H, m)	26.2	4.01 (1H, m)	69.3	4.02 (1H, ddd, 5.2, 10.7, 15.7)	39.2
12	1.79 ^a^ (2H, m)	30.9	1.24 ^a^ (1H, m) 2.30 ^a^ (1H, m)	51.9	1.26 ^a^ (1H, m) 2.28 ^a^ (1H, m)	51.8
13		44.5		43.1		43.0
14		50.3	1.13 ^a^ (1H, m)	55.3	1.21 ^a^ (1H, m)	55.8
15	1.21 (1H, ddd, 1.9, 9.6, 11.8) 1.61 ^a^ (1H, m)	30.8	1.10 ^a^ (1H, m) 1.61 ^a^ (1H, m)	24.1	1.09 ^a^ (1H, m) 1.58 ^a^ (1H, m)	24.1
16	1.33 ^a^ (1H, m) 1.93 ^a^ (1H, m)	28.0	1.26 ^a^ (2H, m)	29.7	1.30 ^a^ (1H, m) 1.76 (1H, dddd, 5.4, 9.0, 9.0, 14.0)	28.9
17	1.51 ^a^ (1H, m)	50.3	1.17 ^a^ (1H, m)	55.9	1.15 ^a^ (1H, m)	55.4
18	0.72 (3H, s)	15.7	0.75 (3H, s)	13.2	0.77 (3H, s)	13.3
19	1.01 (3H, s)	19.0	1.32 (3H, s)	18.3	1.32 (3H, s)	18.3
20	1.40 ^a^ (1H, m)	36.2	1.37 ^a^ (1H, m)	36.1	2.04 ^a^ (1H, m)	40.4
21	0.93 (3H, d, 6.6)	18.7	0.94 (3H, d, 6.1)	18.6	1.04 (3H, d, 6.6)	21.1
22	1.05 ^a^ (1H, m) 1.42 ^a^ (1H, m)	36.4	1.02 ^a^ (1H, m) 1.32 ^a^ (1H, m)	33.7	5.13 (1H, dd, 8.5, 15.1)	137.7
23	1.86 ^a^ (1H, m) 2.04 ^a^ (1H, m)	24.9	1.16 ^a^ (2H, m)	26.0	5.04 (1H, dd, 8.8, 14.9)	129.5
24	5.10 ^a^ (1H, dd, 7.2, 7.2)	125.2	0.93 ^a^ (1H, m)	45.7	1.54 ^a^ (1H, m)	51.2
25		131.2	1.66 ^a^ (1H, m)	29.1	1.54 ^a^ (1H, m)	31.0
26	1.60 (3H, s)	17.6	0.81 (3H, d, 6.6)	19.0	0.80 ^a^ (3H, m)	19.0
27	1.68 (3H, s)	25.7	0.84 ^a^ (3H, m)	19.8	0.85 (3H, d, 6.6)	21.1
28	0.92 ^a^ (3H, s)	25.1	1.23 ^a^ (1H, m) 1.27 ^a^ (1H, m)	23.0	1.17 ^a^ (1H, m) 1.43 ^a^ (1H, m)	25.4
29	1.02 ^a^ (3H, d, 5.5)	115.0	0.84 ^a^ (3H, m)	12.0	0.80 ^a^ (3H, m)	12.2

^a^ overlapped signal.

**Table 7:** ^1^H and ^13^C NMR data of compound **43** (600 and 151 MHz, respectively) in CD_3_OD (*δ* in ppm, *J* in Hz, s: singlet, d: doublet, t: triplet, q: quartet, br: broad, and m: multiplet).

No.	43	
	*δ*_H_	*δ*_C_
1		132.5
2		124.9
3		152.6
4		142.3
5		154.2
6	6.67 (1H, s)	109.7
7	2.51 (1H, dd, 1.9, 15.7) 2.75 (1H, dd, 7.7, 15.4)	32.9
8	3.18 (1H, m)	33.4
9	3.92 (1H, dd, 3.3, 9.4) 4.49 (1H, dd, 7.8, 9.1)	75.1
10	3.76 (3H, s)	61.6
11	3.82 (3H, s)	61.3
12	3.85 (3H, s)	56.5
1′		137.8
2′	6.35 (1H, brs)	108.7
3′		144.9
4′		135.1
5′		150.6
6′	6.29 (1H, brs)	102.4
7′	4.83 ^a^ (1H, m)	39.7
8′	3.62 (1H, dd, 2.2, 9.9)	46.3
9′		181.3
10′	3.79 (3H, s)	57.4
11′	5.86 (1H, s) 5.92 (1H, s)	102.5

^a^ overlapped signal.

**Table 8:** Water/ACN gradients for the separation of the respective fractions on a biphenyl column (M1.2C4: Nucleodur C18 column) and retention times of the isolated compounds.

Fraction	Gradient		Retention Time [min], Compound No.
	Time [min]	ACN [%]	
HEP8C4	0 11 11.1 13	70 87 100 100	7.5, **38**; 9.5, **42**; 9.8, **40**; 10.2, **39**
HEP8C6	0 20 25	60 100 100	19.7 min, **41**
M1.2C4	0 15 16 25	55 75 100 100	10.5, **29**; 12.0 min, curzerenone (2.1 mg); 15.0 min, myrrhone (6.4 mg)
M1.4R1F1	0 15 20 25	30 40 90 90	9.5, **16**; 10.0, **10**; 12.3, mixture of **3**, **4** and **22**; 15.0, **26**; 20.8, **43**
M1.4R1F4	0 20 21 26	20 32 90 90	11.6, **12**; 12.0, **24**; 13.6, **11**; 14.0, **23**; 15.9, **15**; 17.3, **5**; 17.6, **27**; 18.1, **2**; 20.5, **8**; 21.4, **6**; 22.1, mixture of **18** and **20**
M1.4R1F6	0 15 16 21	20 30 90 90	12.4, **7**; 13.2 **13**; 15.5, **17**
M1.4R1F7.2			4.9, **1**, 6.6, **14**; 14.4, **9**
M1.4R1F7.1	0 25 26 31	17 23 95 95	24.4, (–)-**21**; 25.2, **25**; 25.6, **19**; 28.0, **28**

## Data Availability

The data presented in this study are available on request from J.H. and A.U.

## Supplementary Materials

The following supporting information can be downloaded at: https://www.mdpi.com/article/10.3390/molecules29184315/s1, Figure S1. ^1^H (600 MHz) and ^13^C NMR (151 MHz) spectrum of compound **1** in CD_3_OD. Figure S2. HSQC and HMBC spectrum of compound **1** in CD_3_OD. Figure S3. COSY and NOESY spectrum of compound **1** in CD_3_OD. Figures S4–S23: ^1^H and ^13^C NMR spectra of compounds **2**–**17**, **38**–**40** and **43**. Figure S24. CD spectra of compounds **1**, **2**, **5**–**17** in MeOH, [*θ*] in [(°cm^2^) × dmol^−1^]. Figure S25**.** CD spectra of **19**, (-)-**21**, **23**–**29**, **36** and **38**–**41** in MeOH, [*θ*] in [(°cm^2^) × dmol^−1^]. Figure S26. CD spectra of compounds **42** and **43** in MeOH, [*θ*] in [(°cm^2^) × dmol^−1^]. Figure S27. Influence of compounds **1**, (–)-**21**, **29**–**37** and furanoeudesma-1,3-diene (FUR) on the viability of RAW 264.7 cells in the MTT assay. The test was performed including a negative control (n.c., medium only) and two solvent controls (s.c. 1: 0.1% DMSO, s.c. 2: 0.14% DMSO, *v*/*v*). Data are presented as mean ± SEM, * *p* < 0.05 vs. n.c. (student’s *t*-test, *n* = 3). Figures S28–S37. ^1^H and ^13^C NMR spectra of compounds **21** and **29**–**37** and **43**.
