# Supplementary material for: An Unprecedented 4,8-Cycloeudesmane, Further New Sesquiterpenoids, a Triterpene, Steroids, and a Lignan from the Resin of Commiphora myrrha and Their Anti-Inflammatory Activity In Vitro
Source: Molecules. 2024 Sep 11;29(18):4315. doi: 10.3390/molecules29184315 (PMC11434423; doi:10.3390/molecules29184315)
Supplement: Supplementary file 1 [file molecules-29-04315-s001.zip › molecules-3175879-supplementary.pdf]

molecules MDPI

Supplementary material

An unprecedented 4,8-Cycloeudesmane, further new Sesquiterpenoids, a Triterpene, Steroids and a Lignan from the resin of *Commiphora myrrha* and their anti-inflammatory activity *in vitro*

Anna Unterholzner [1], Katrin Kuck [1], Anna Weinzierl [1], Bartosz Lipowicz [2], and Jörg Heilmann [1,*]

[1] Institute of Pharmaceutical Biology, University of Regensburg, Universitätsstr. 31, D-93053 Regensburg, Germany
[2] Repha GmbH Biologische Arzneimittel, Alt-Godshorn 87, D-30855 Langenhagen, Germany
* Correspondence: joerg.heilmann@chemie.uni-regensburg.de; Tel.: +49-941-9434761

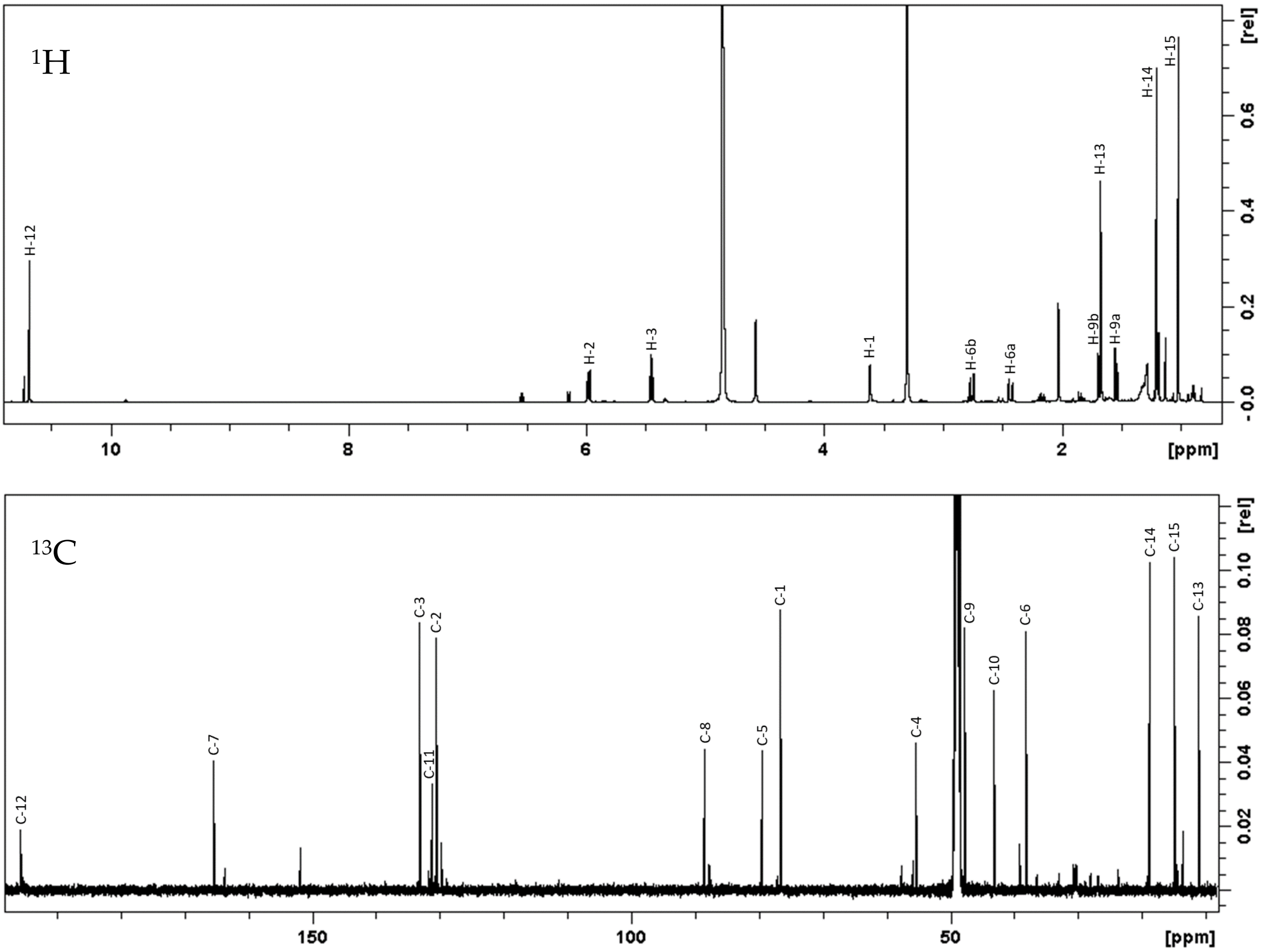

Figure S1. ^{1}H (600 MHz) and ^{13}C NMR (151 MHz) spectrum of compound **1** in CD_3OD.

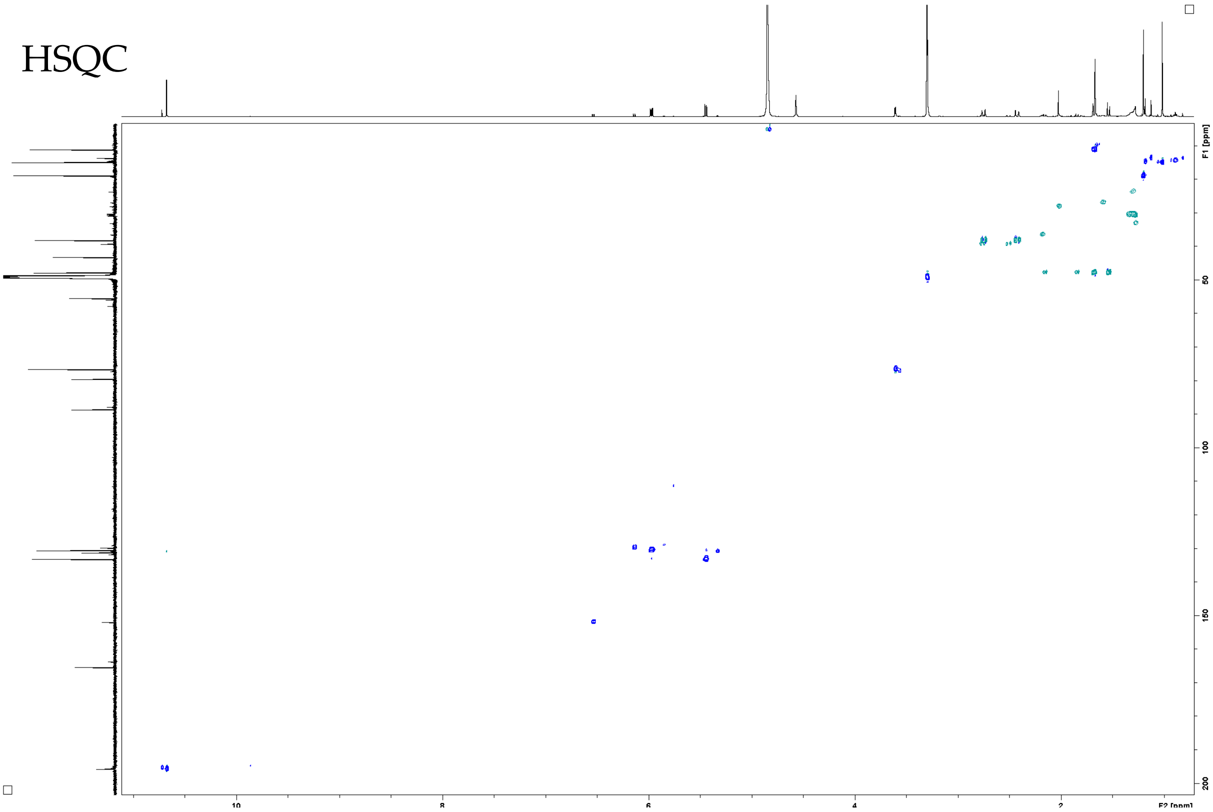

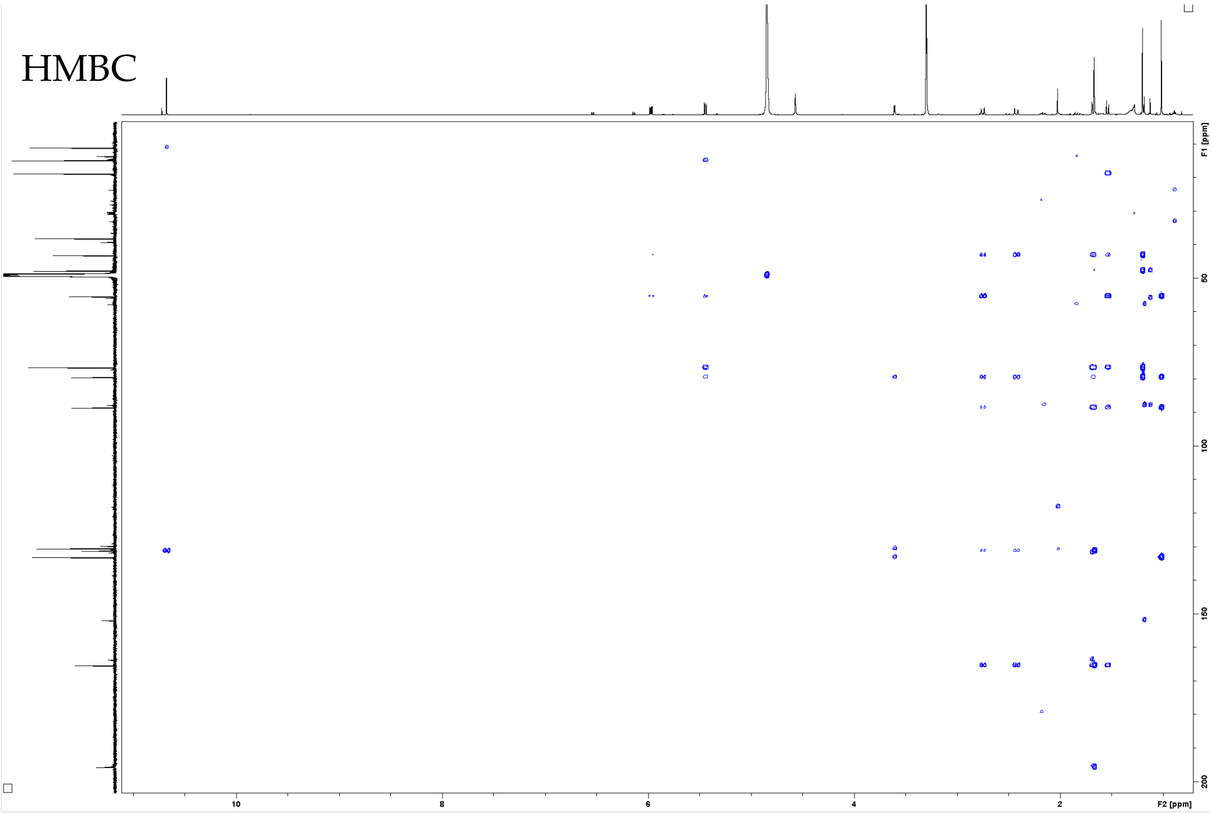

Figure S2. HSQC and HMBC spectrum of compound **1** in CD3OD.

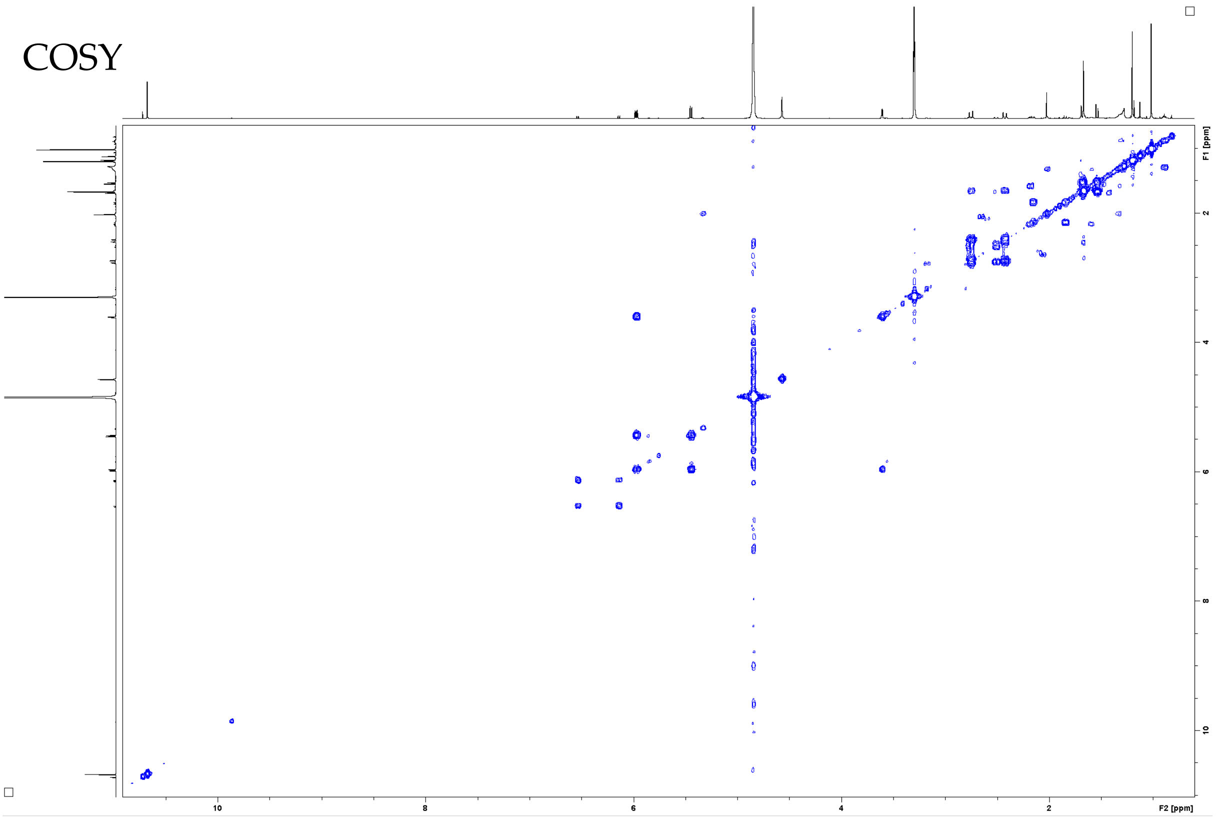

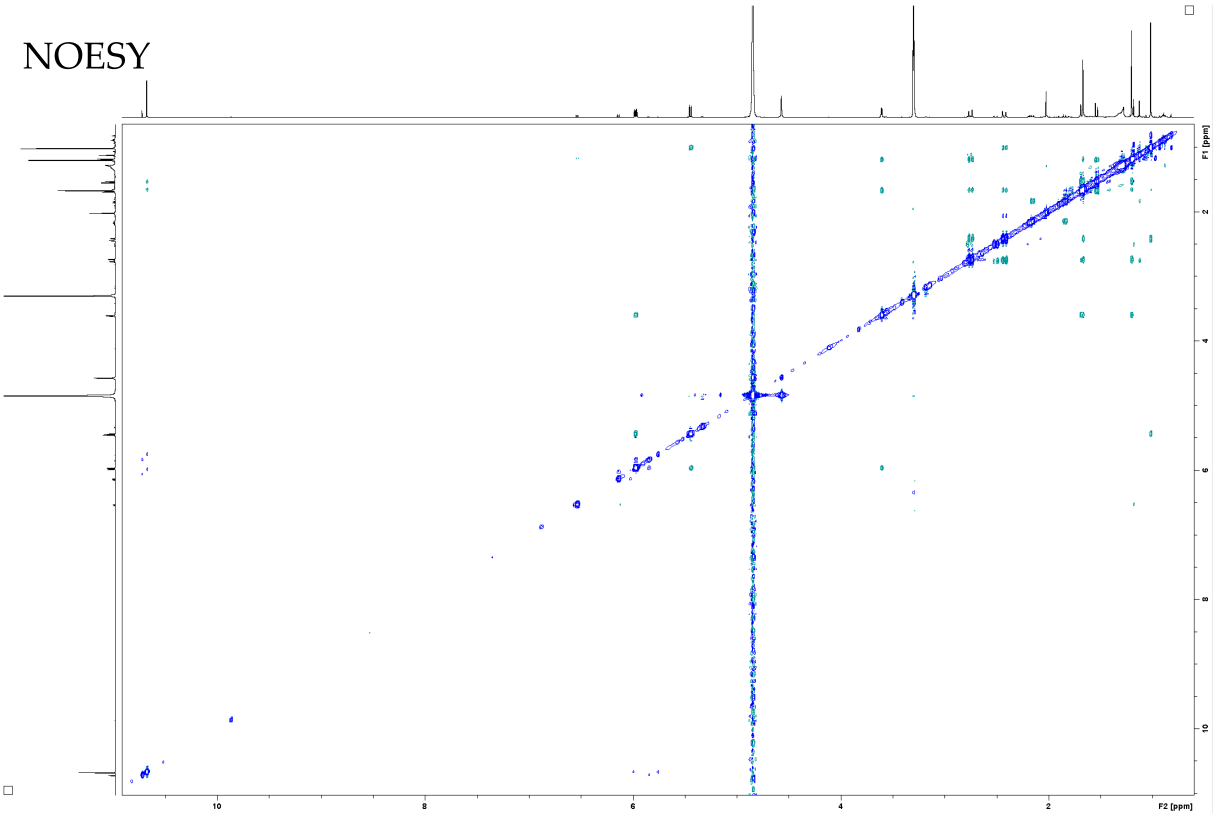

Figure S3. COSY and NOESY spectrum of compound **1** in CD_3OD.

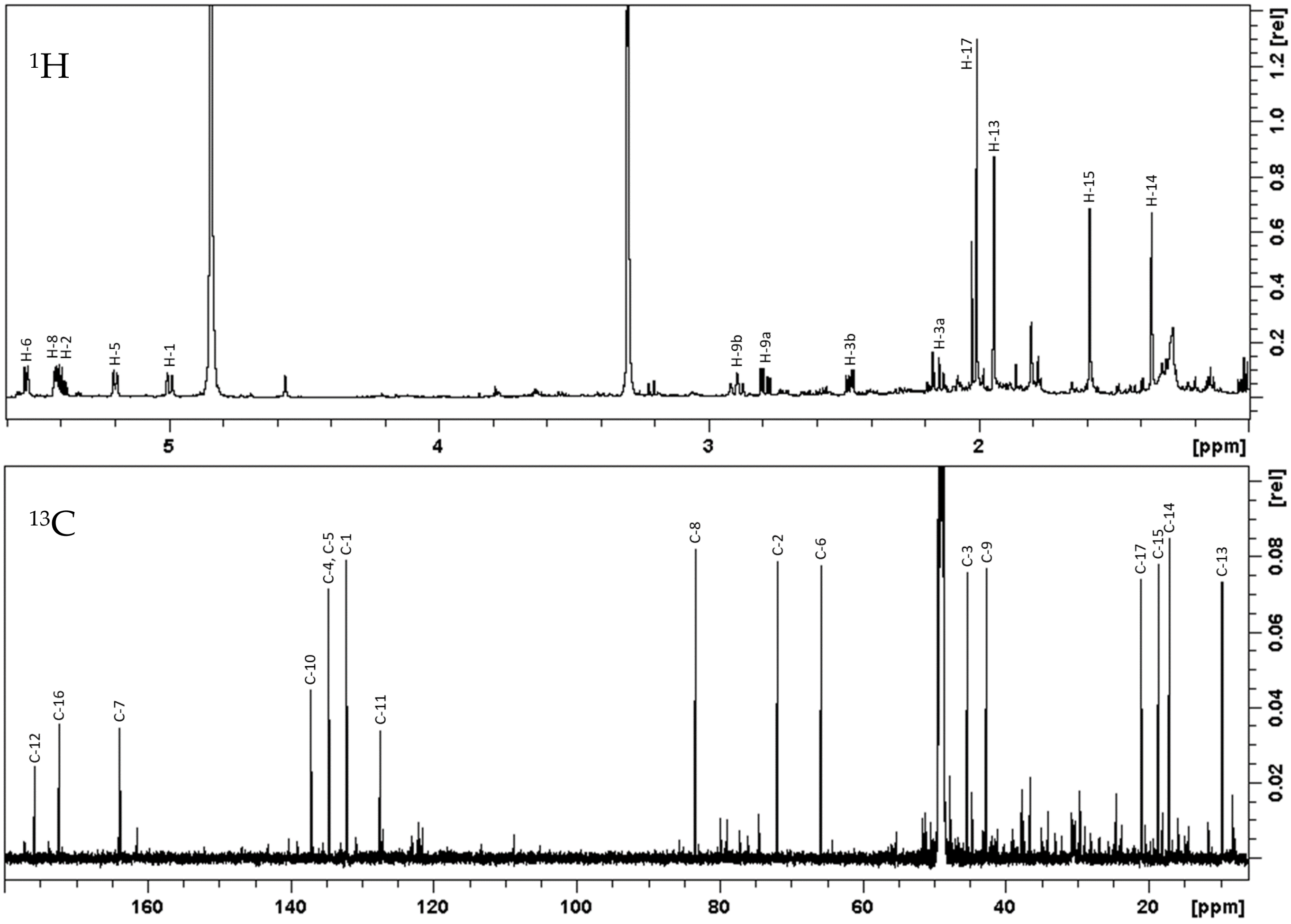

Figure S4. ^{1}H (600 MHz) and ^{13}C NMR (151 MHz) spectrum of compound **2** in CD_3OD.

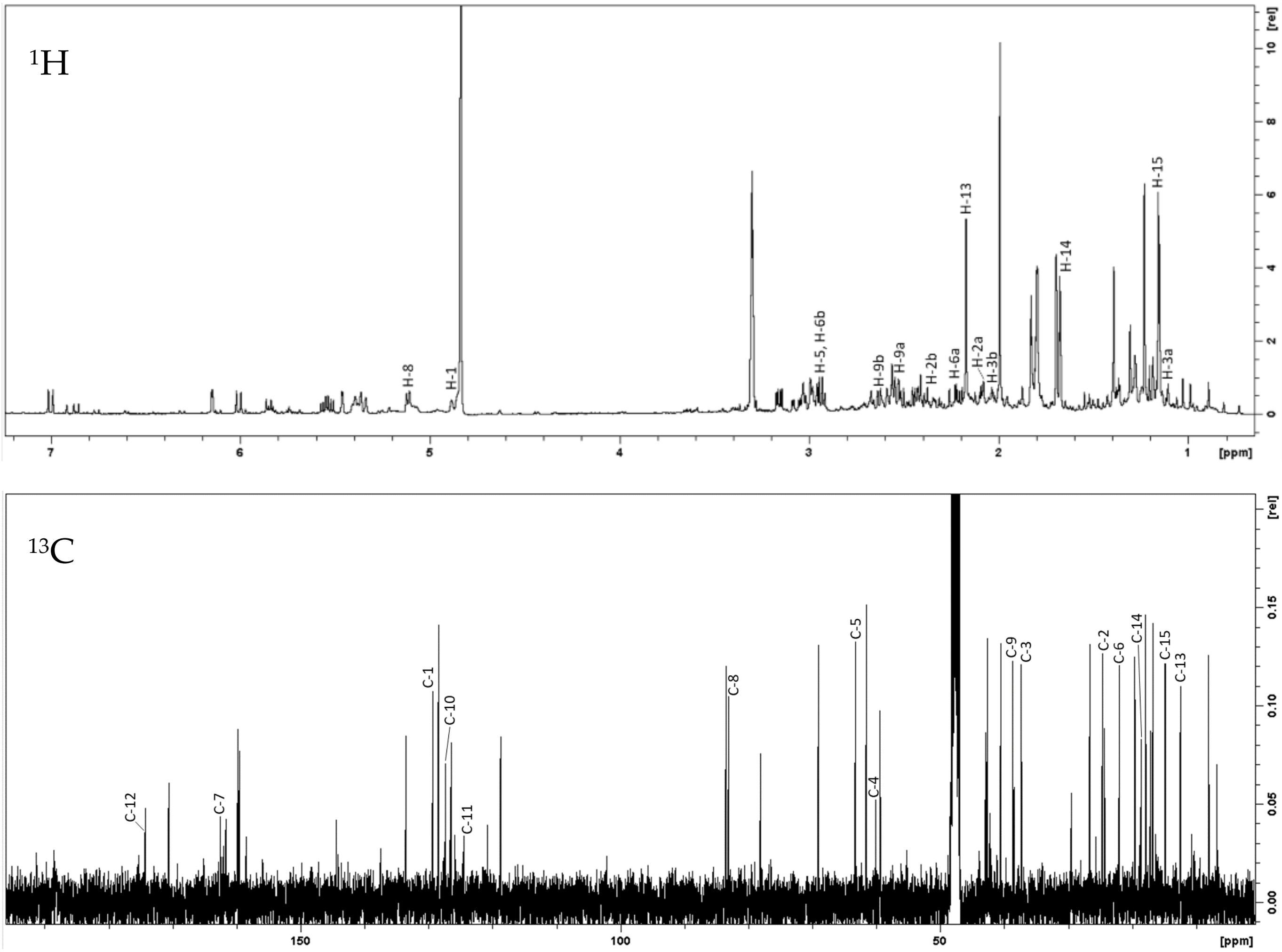

Figure S5. ^{1}H (400 MHz) and ^{13}C NMR (101 MHz) spectrum of the mixture of compounds **3**, **4** and **22** in CD_3OD. Signals of compound **3** are labelled.

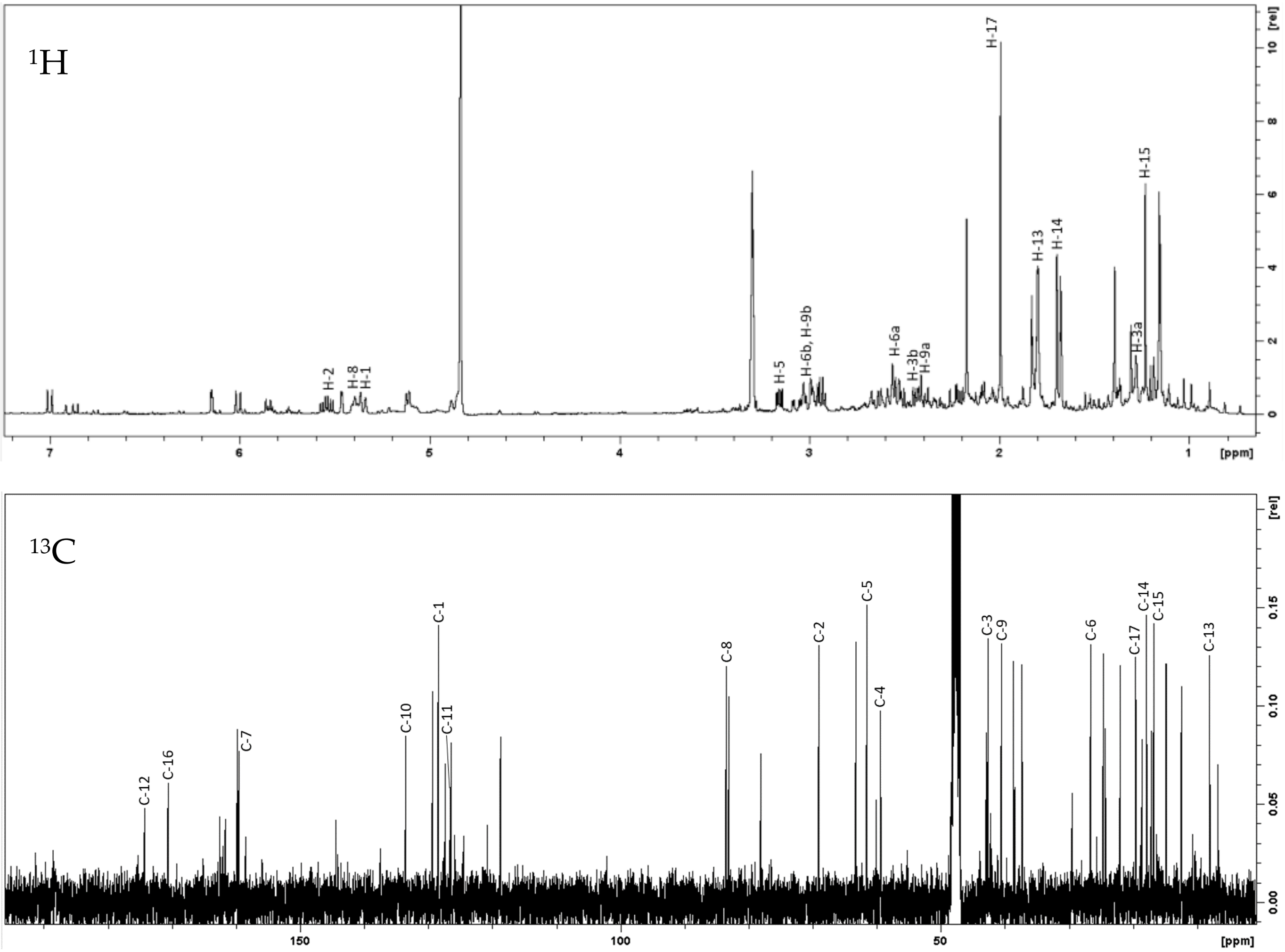

Figure S6. ^{1}H (400 MHz) and ^{13}C NMR (101 MHz) spectrum of the mixture of compounds **3**, **4** and **22** in CD_3OD. Signals of compound **4** are labelled.

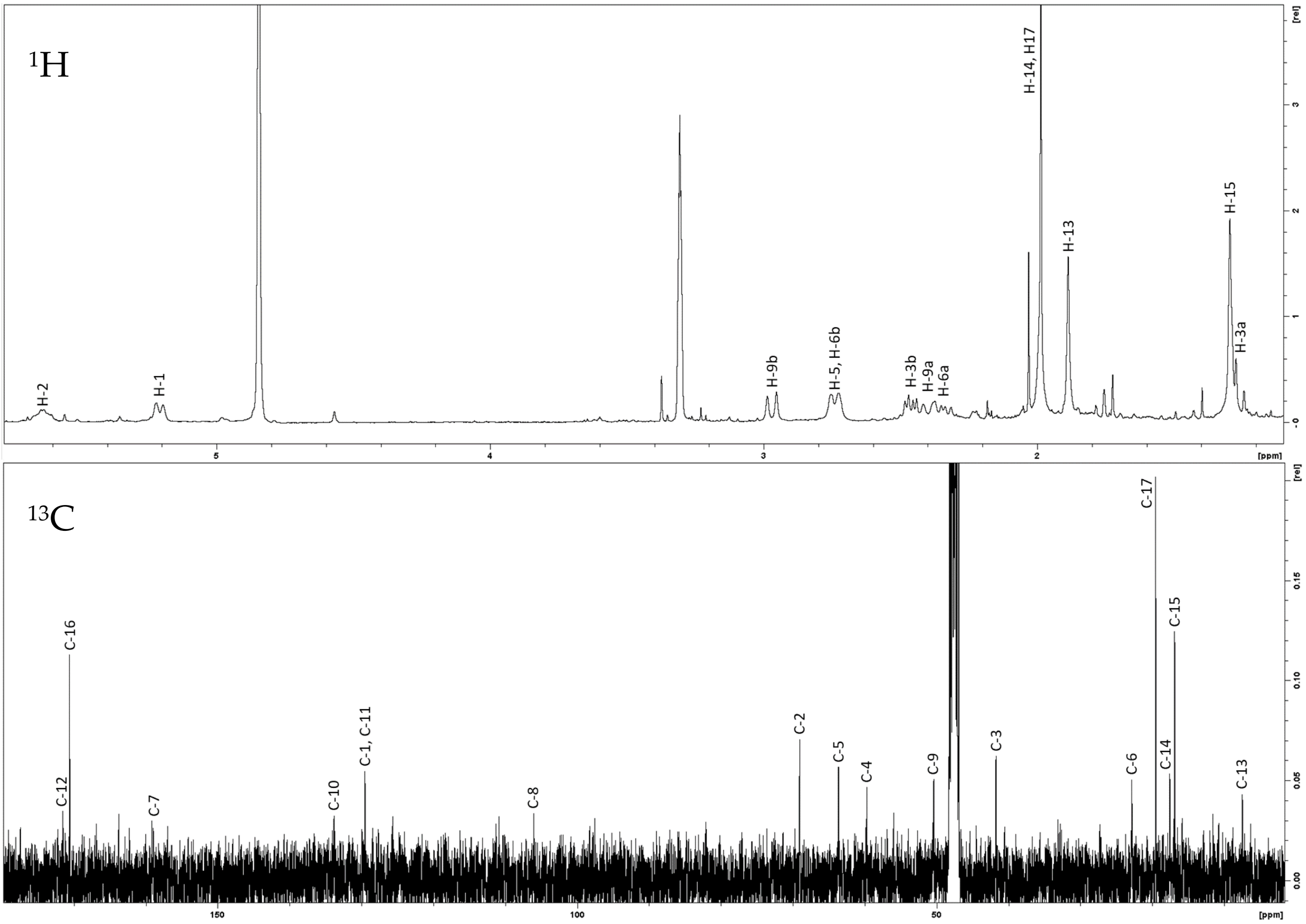

Figure S7. ¹H (400 MHz) and ¹³C NMR (101 MHz) spectrum of compound **5** in CD_3OD.

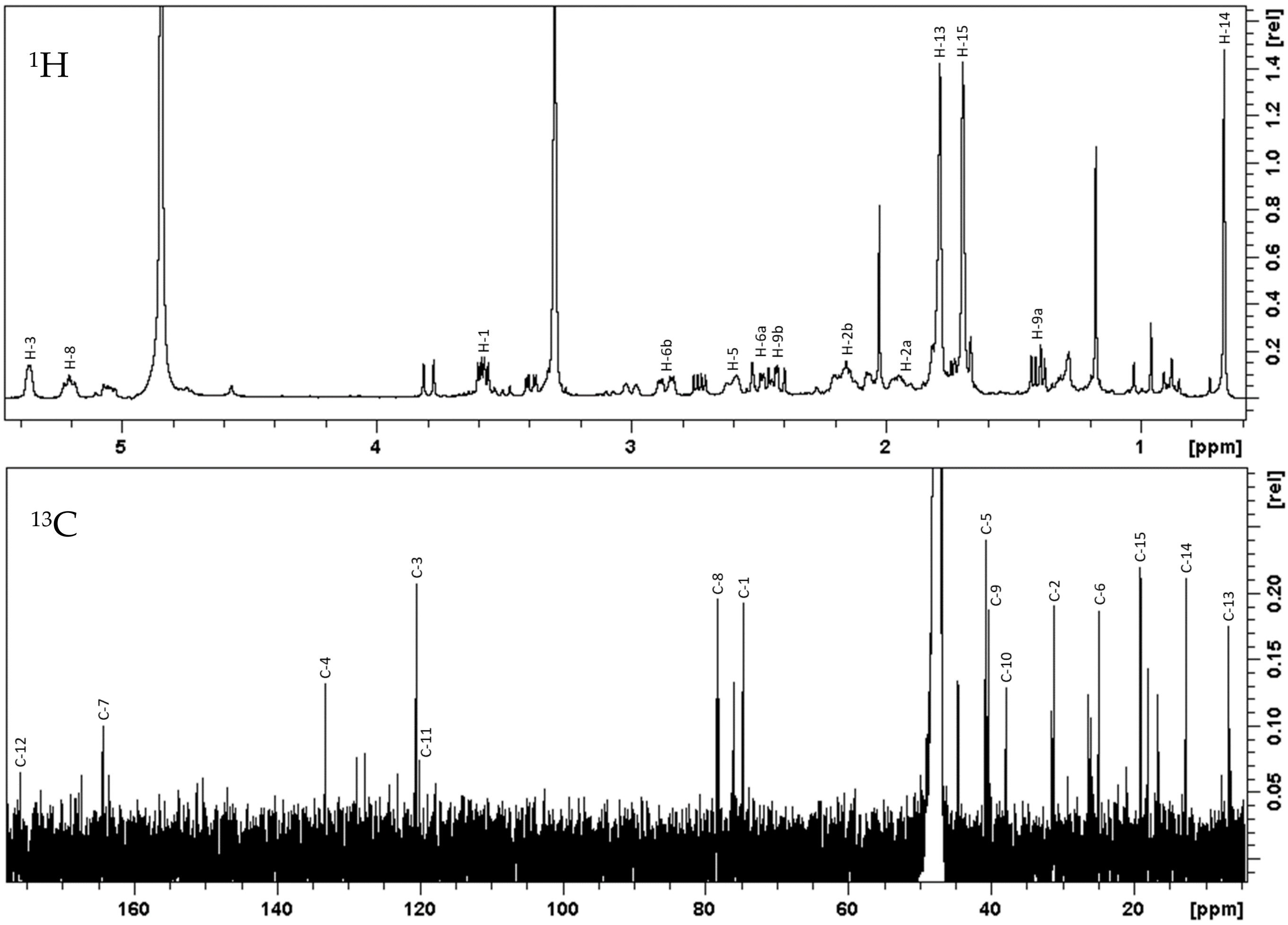

Figure S8. ^{1}H (400 MHz) and ^{13}C NMR (101 MHz) spectrum of compound **6** in CD_3OD.

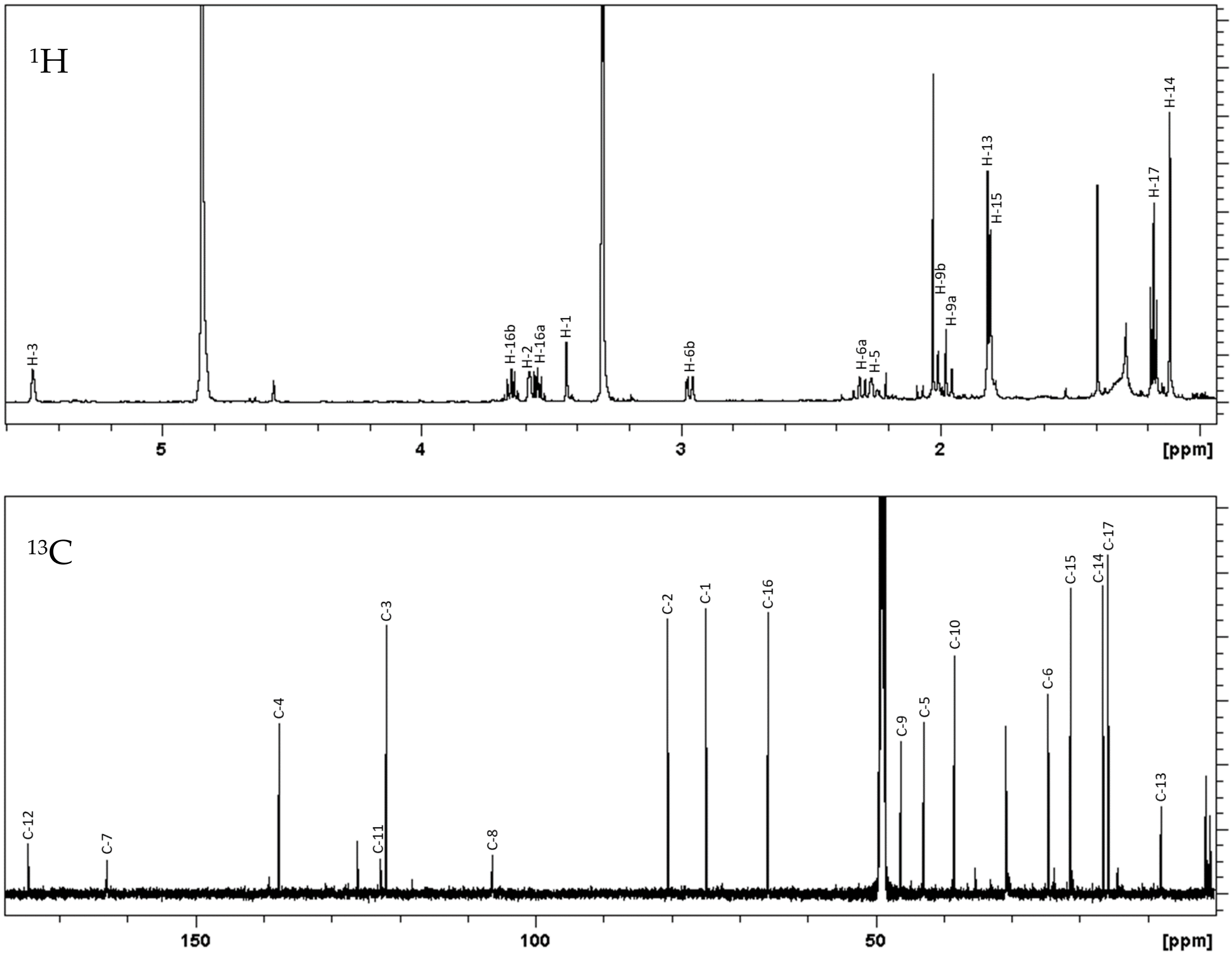

Figure S9. ^{1}H (600 MHz) and ^{13}C NMR (151 MHz) spectrum of compound **7** in CD_3OD.

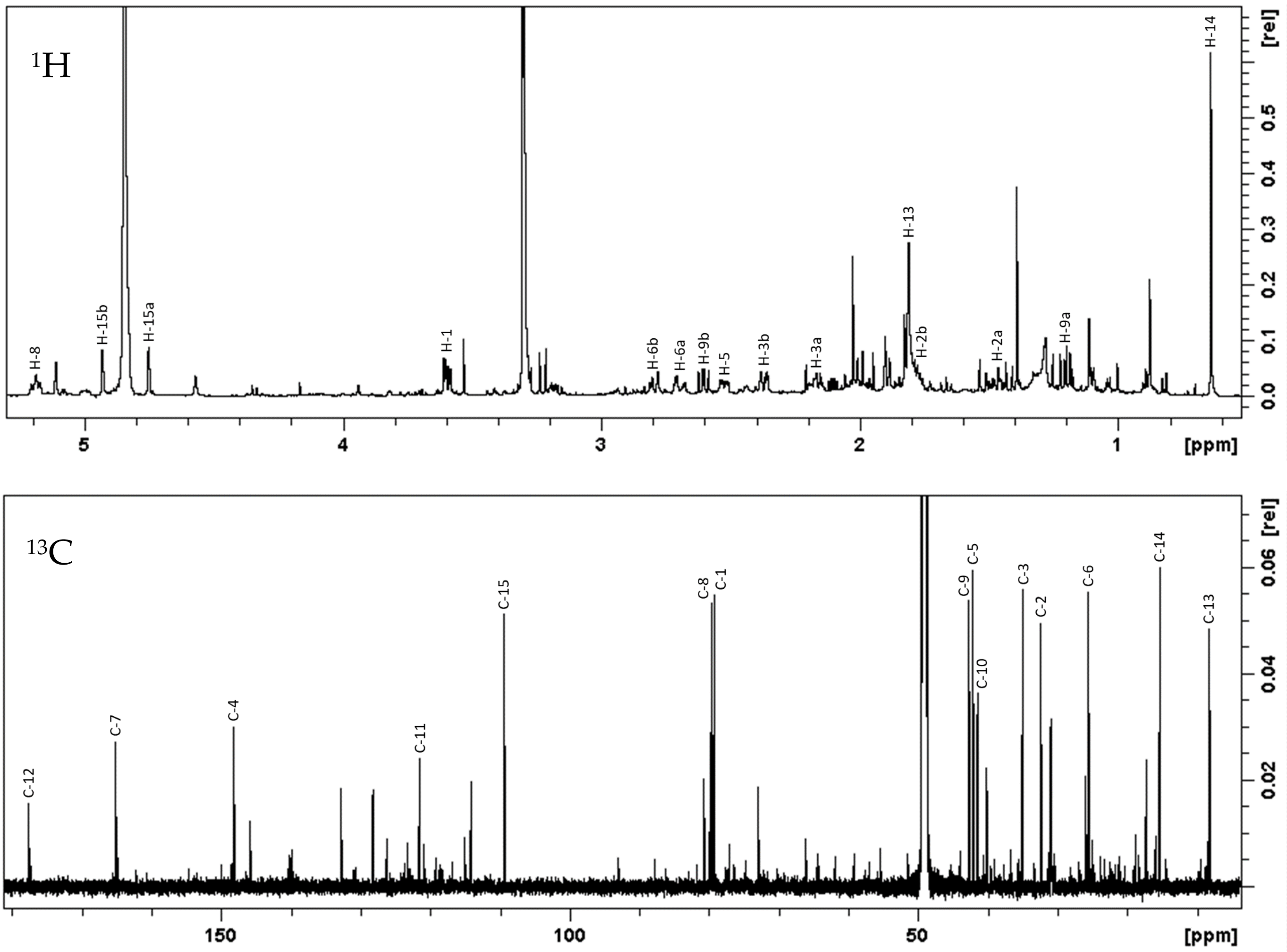

Figure S10. ^{1}H (600 MHz) and ^{13}C NMR (151 MHz) spectrum of compound **8** in CD_3OD.

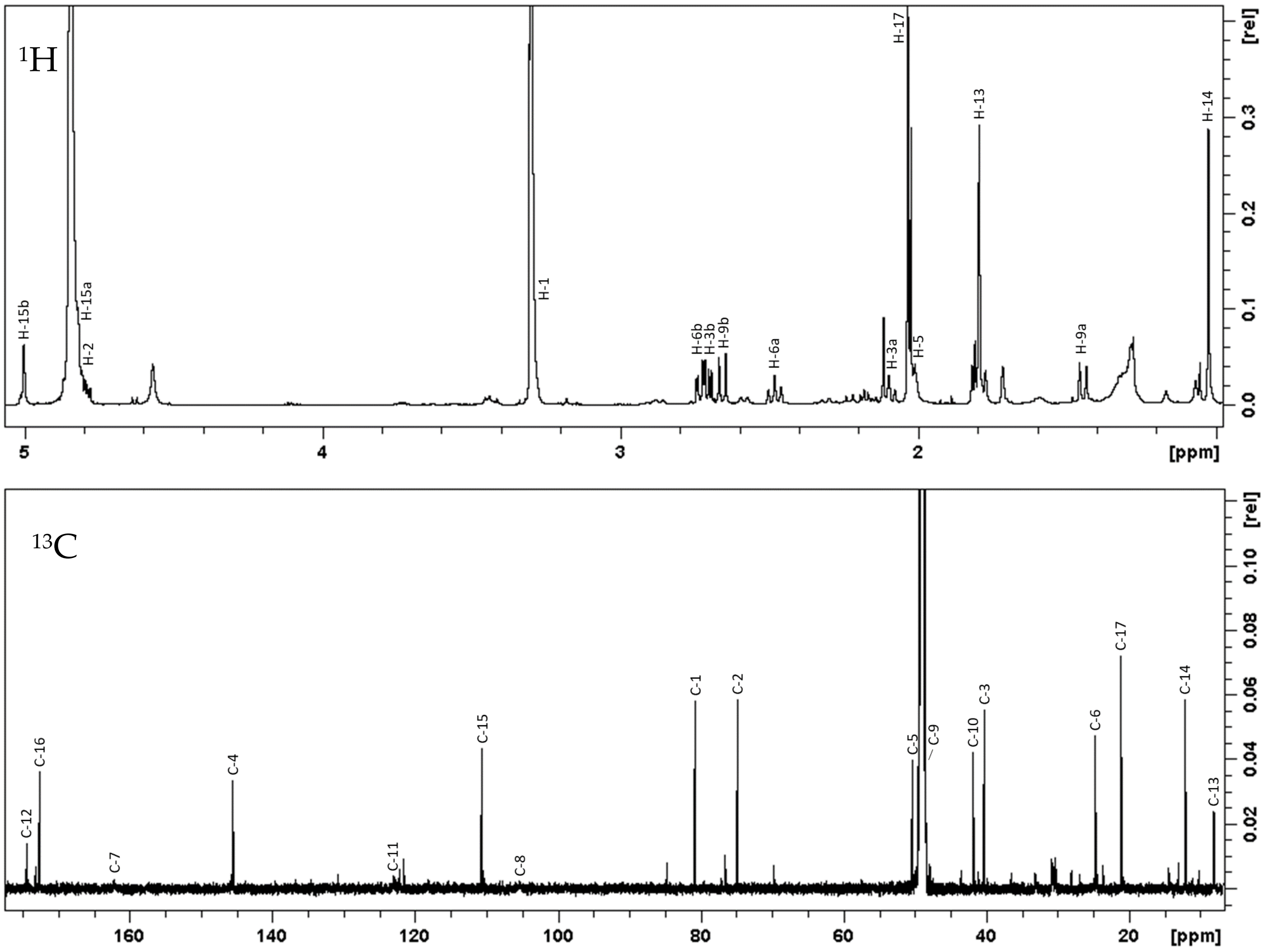

Figure S11. ^{1}H (600 MHz) and ^{13}C NMR (151 MHz) spectrum of compound **9** in CD_3OD.

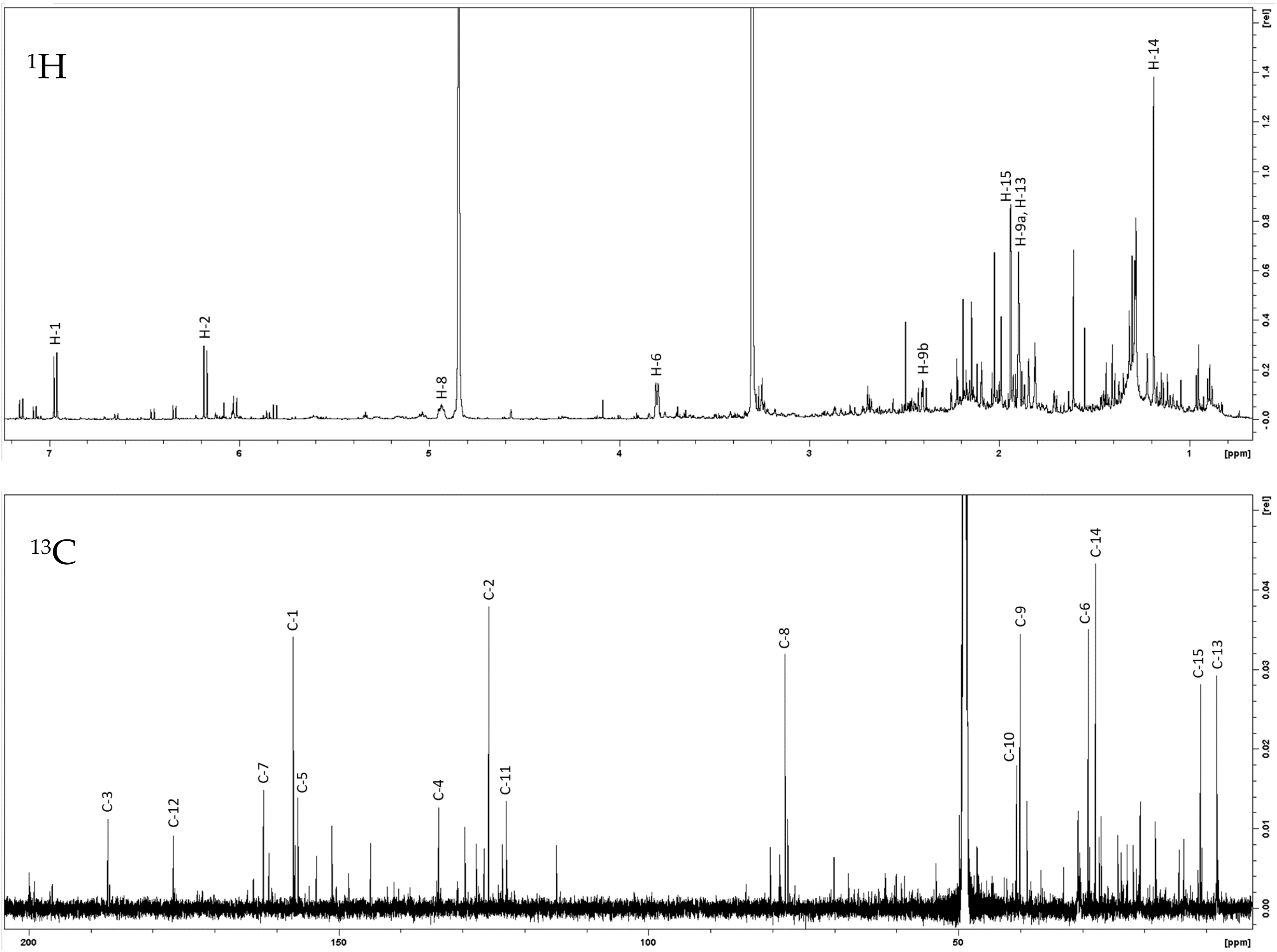

Figure S12. ^{1}H (600 MHz) and ^{13}C NMR (151 MHz) spectrum of compound **10** in CD_3OD.

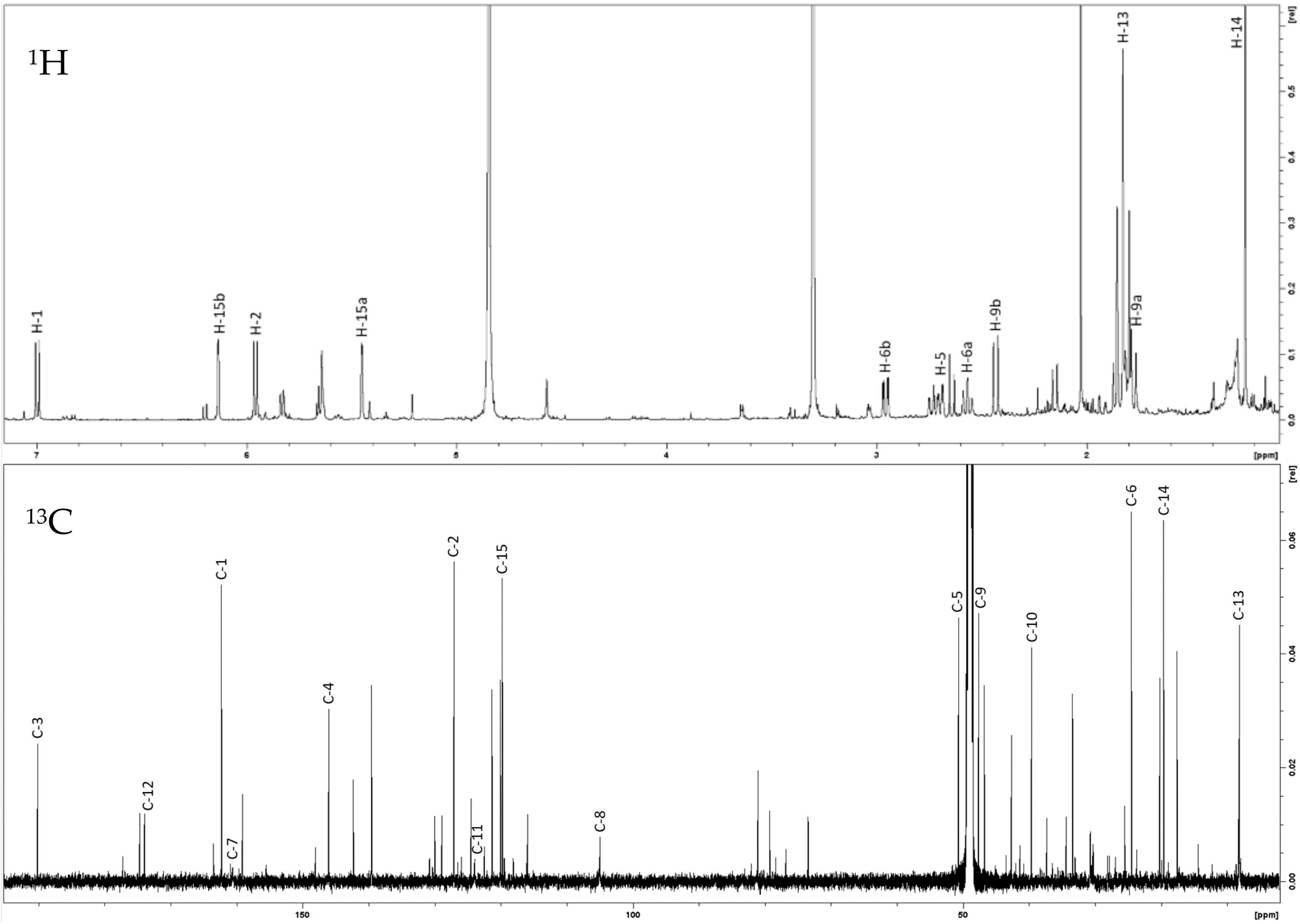

Figure S13. ^{1}H (600 MHz) and ^{13}C NMR (151 MHz) spectrum of compound **11** in CD_3OD.

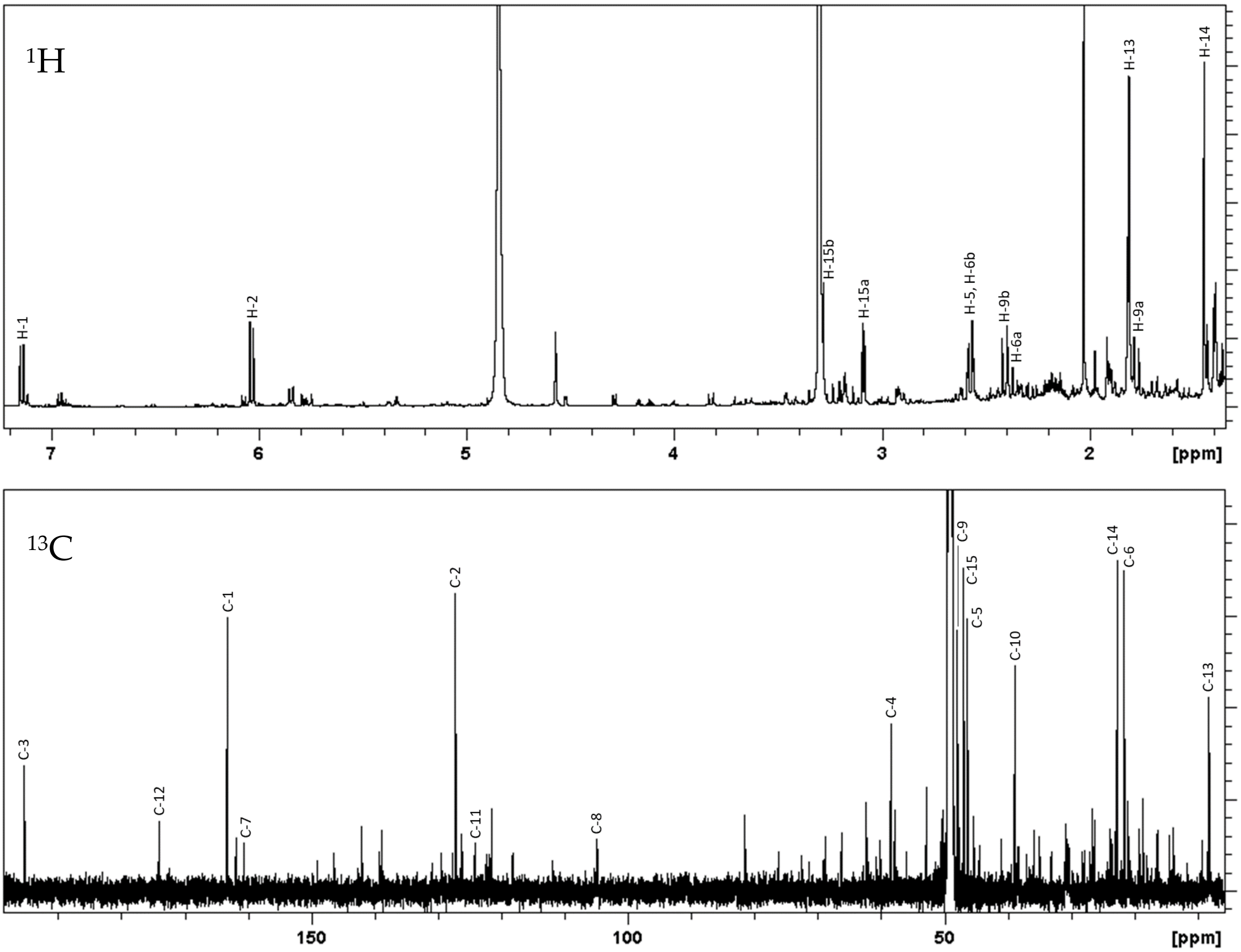

Figure S14. ^{1}H (600 MHz) and ^{13}C NMR (151 MHz) spectrum of compound **12** in CD_3OD.

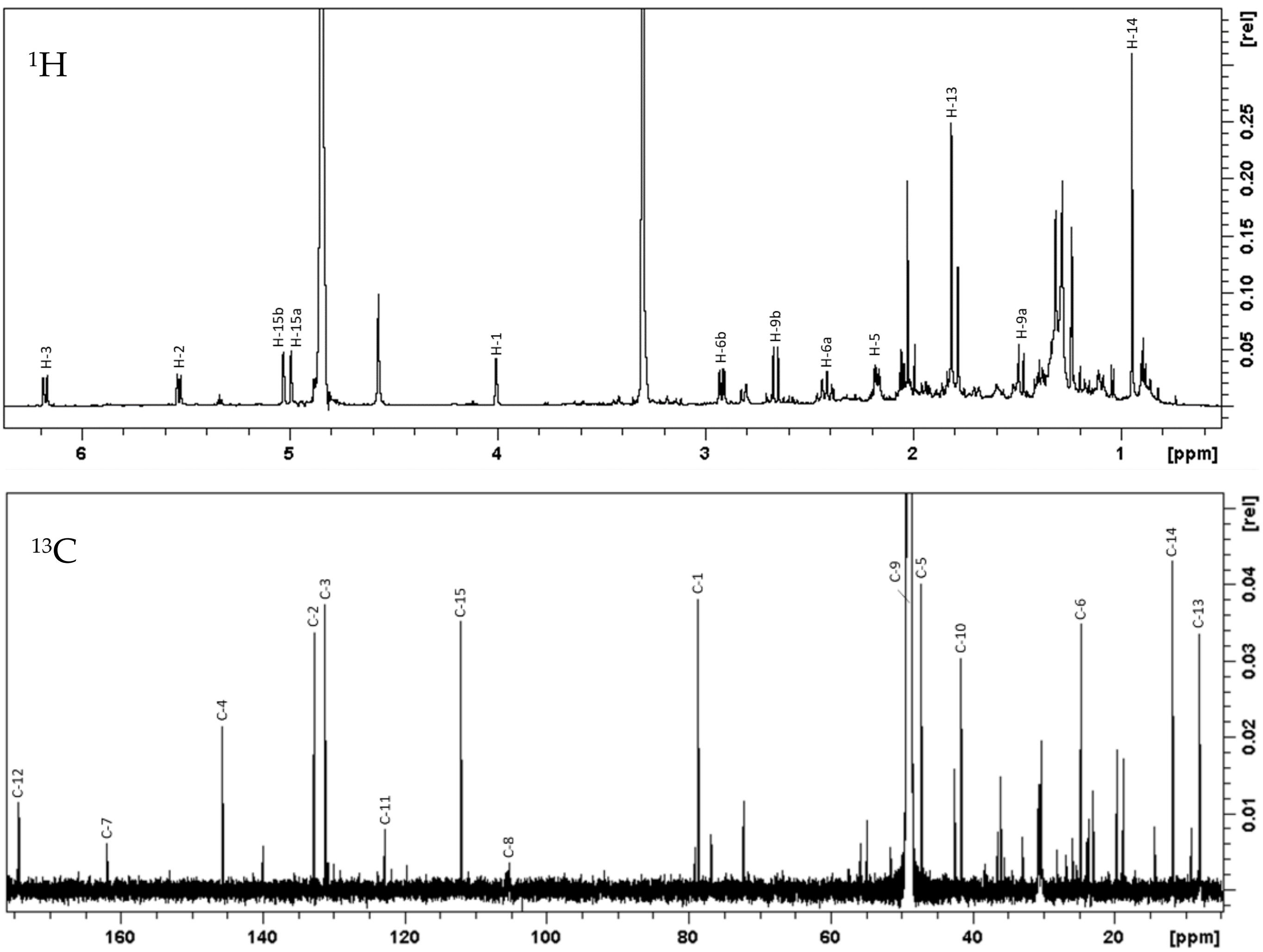

Figure S15. ^{1}H (600 MHz) and ^{13}C NMR (151 MHz) spectrum of compound **13** in CD_3OD.

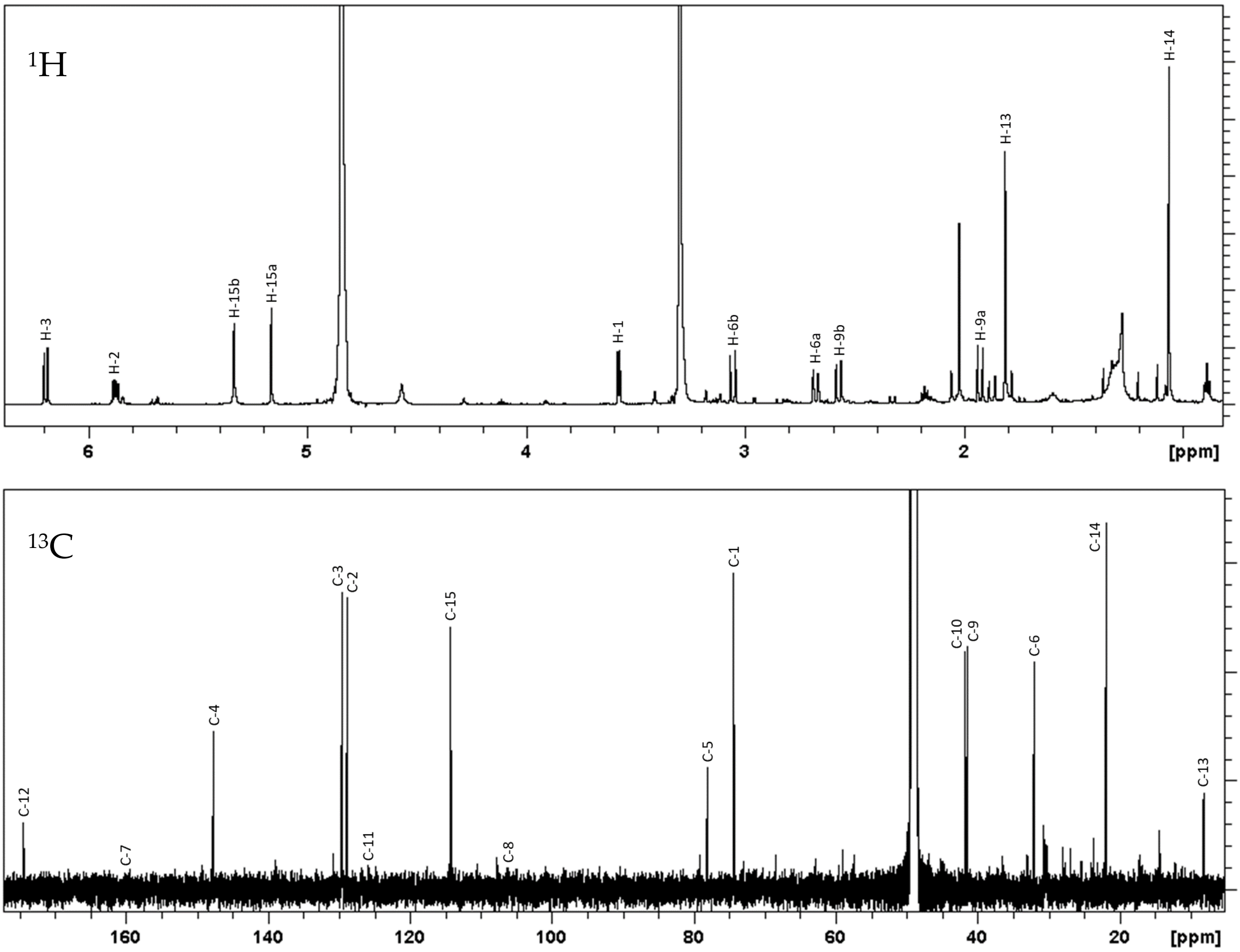

Figure S16. ^{1}H (600 MHz) and ^{13}C NMR (151 MHz) spectrum of compound **14** in CD_3OD.

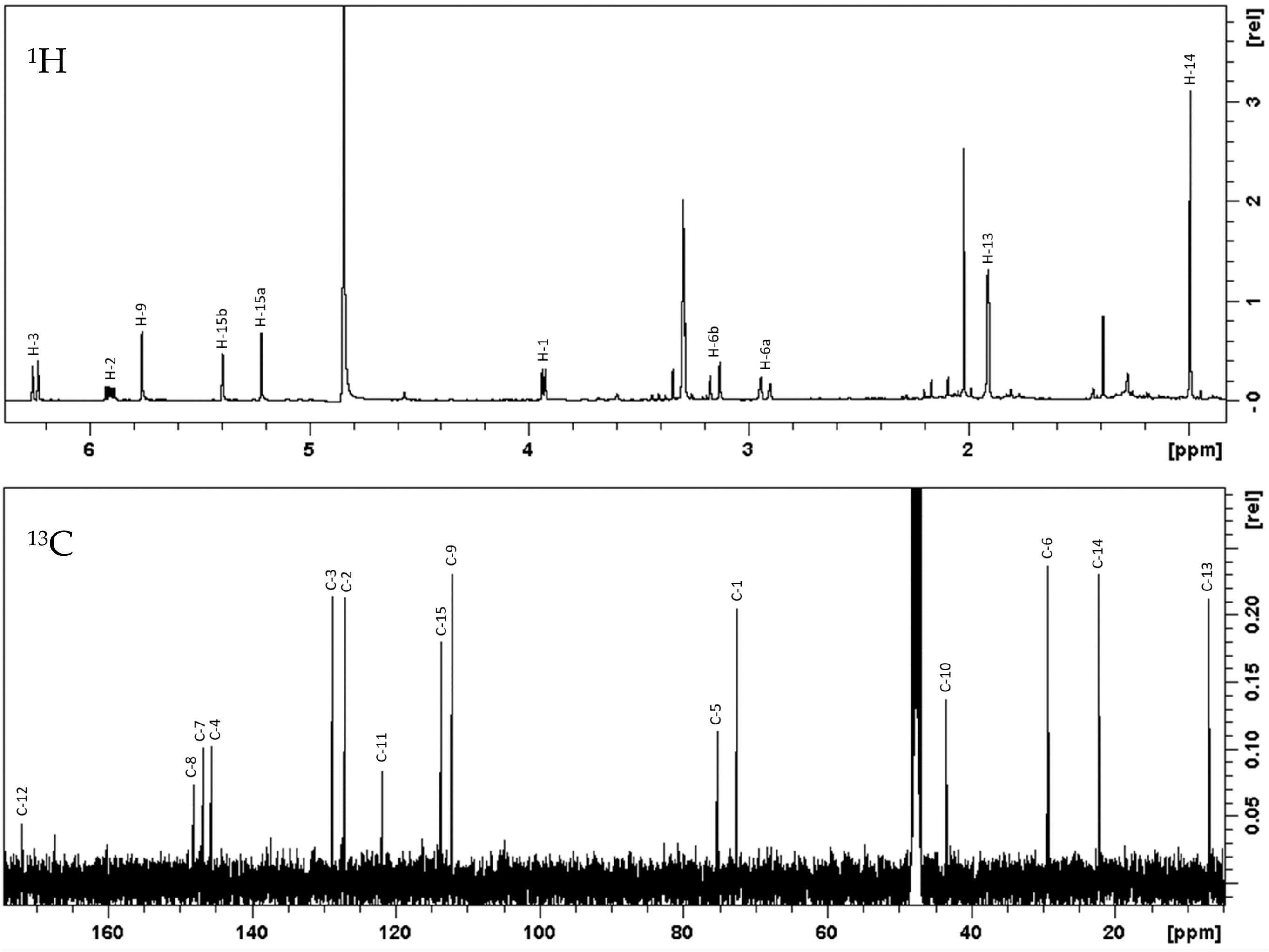

Figure S17. ^{1}H (400 MHz) and ^{13}C NMR (101 MHz) spectrum of compound **15** in CD_3OD.

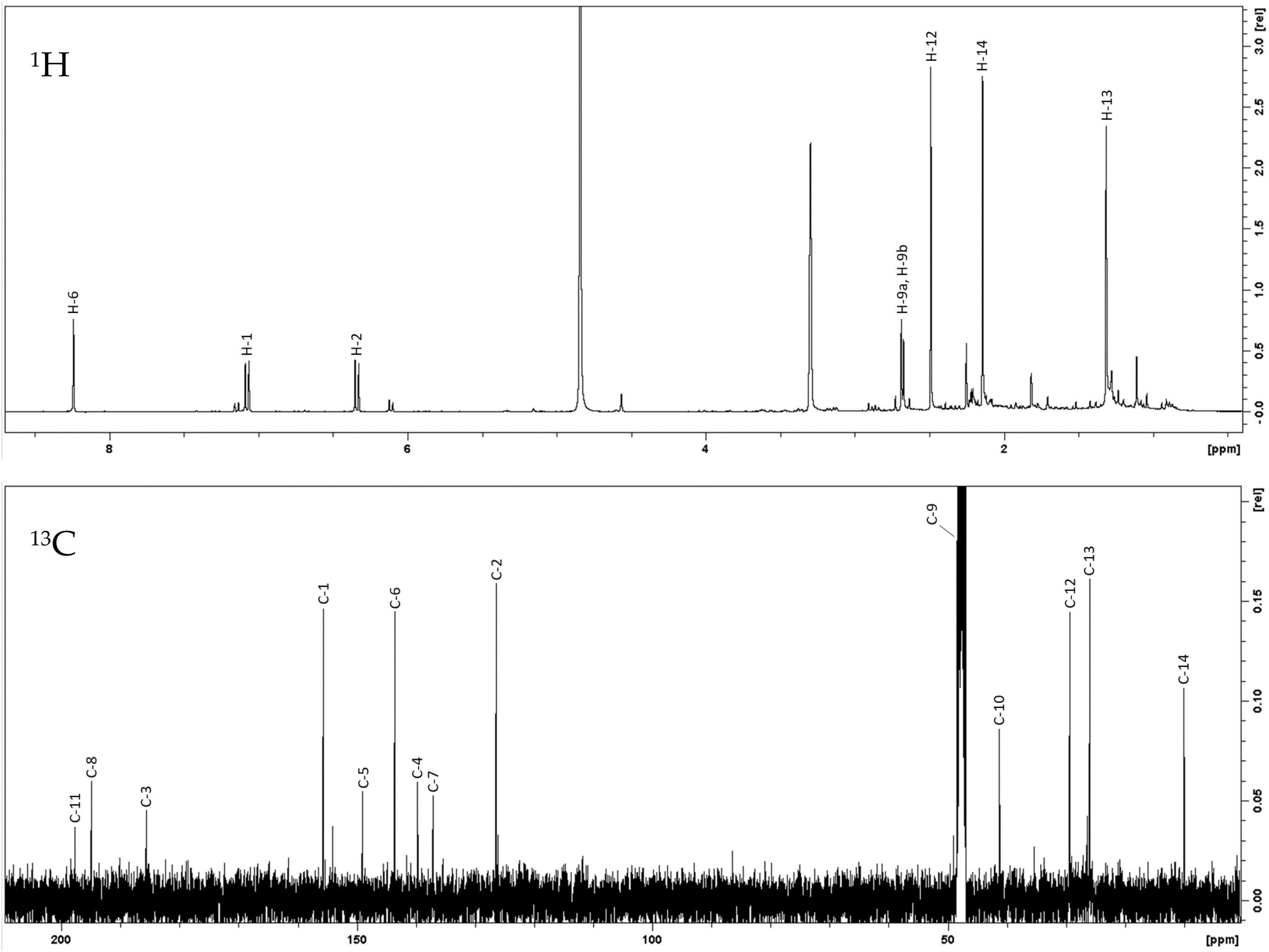

Figure S 18. ^{1}H (400 MHz) and ^{13}C NMR (101 MHz) spectrum of compound **16** in CD_3OD.

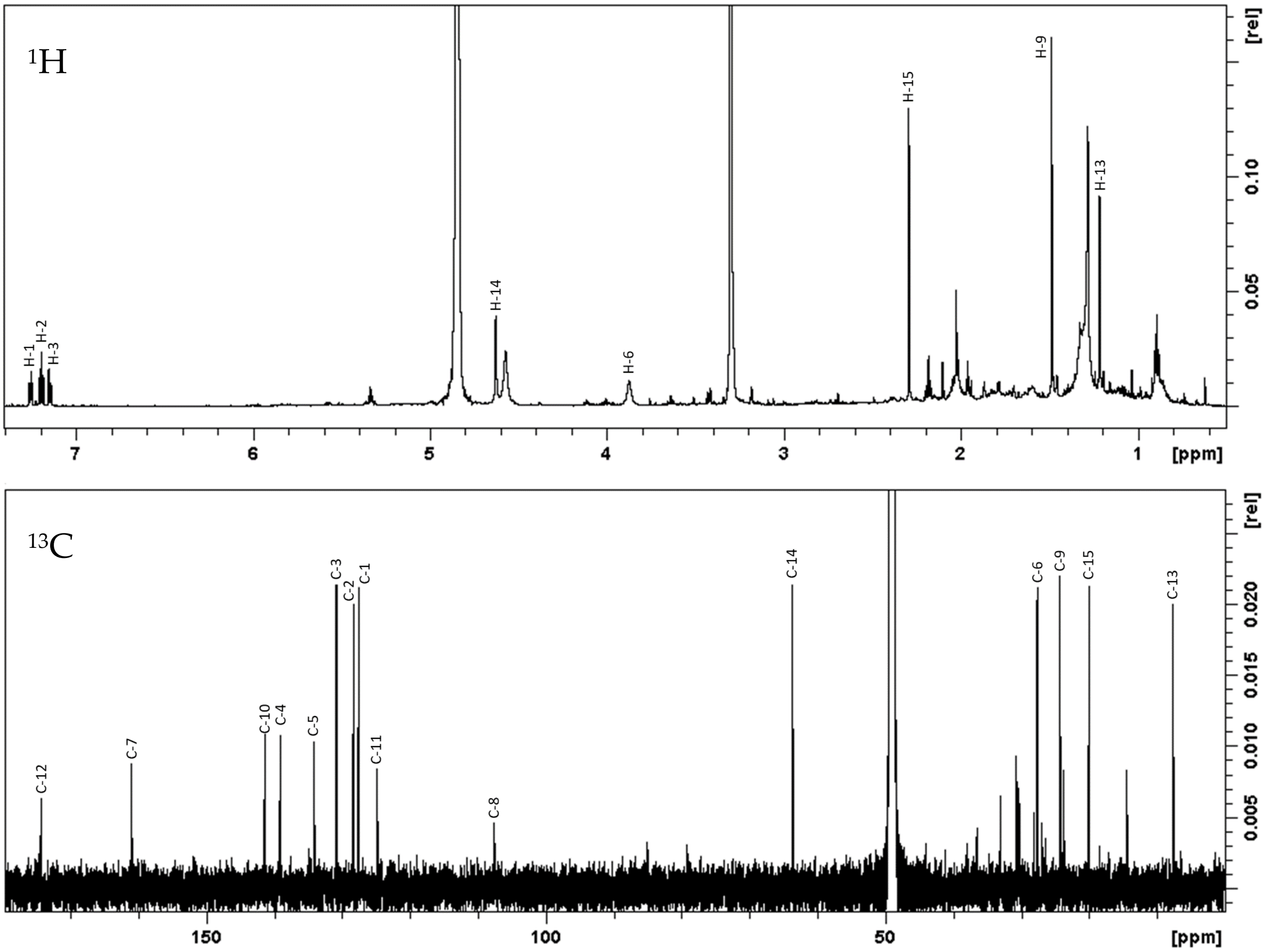

Figure S19. ^{1}H (600 MHz) and ^{13}C NMR (151 MHz) spectrum of compound **17** in CD_3OD.

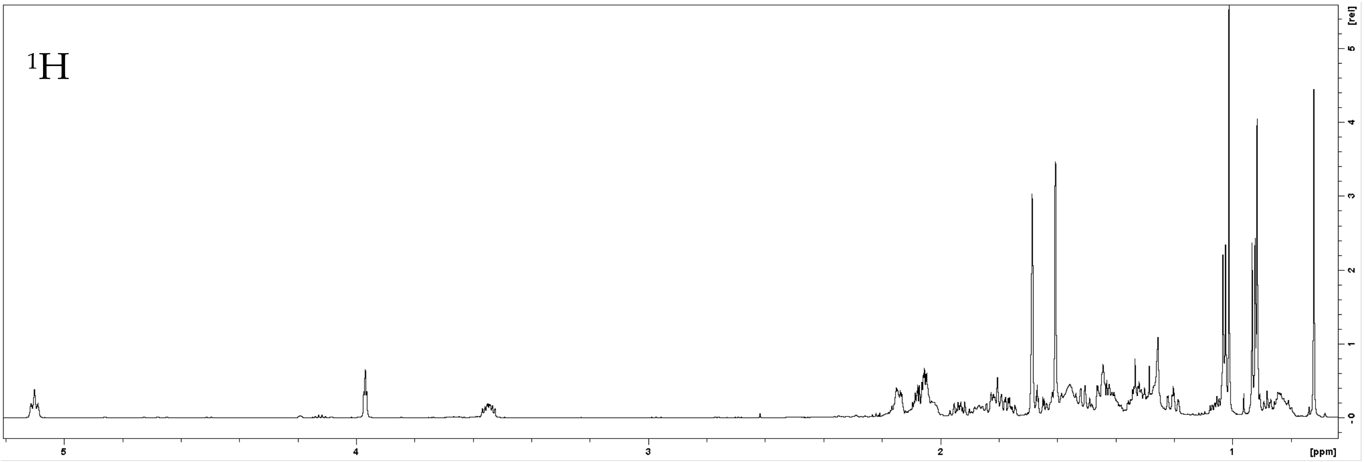

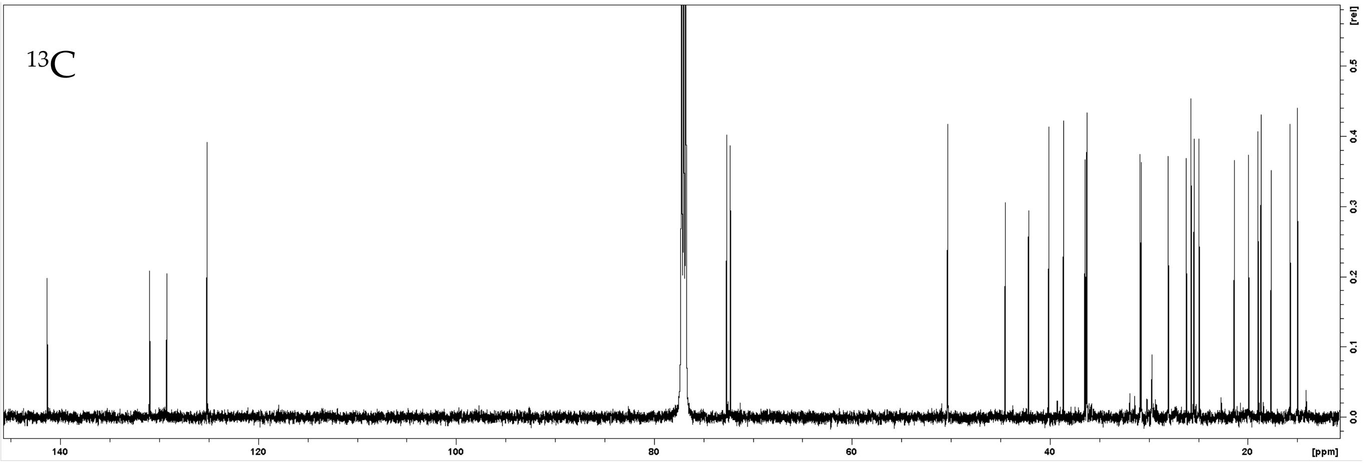

Figure S20. ^{1}H (600 MHz) and ^{13}C NMR (151 MHz) spectrum of compound **38** in $CDCl_3$.

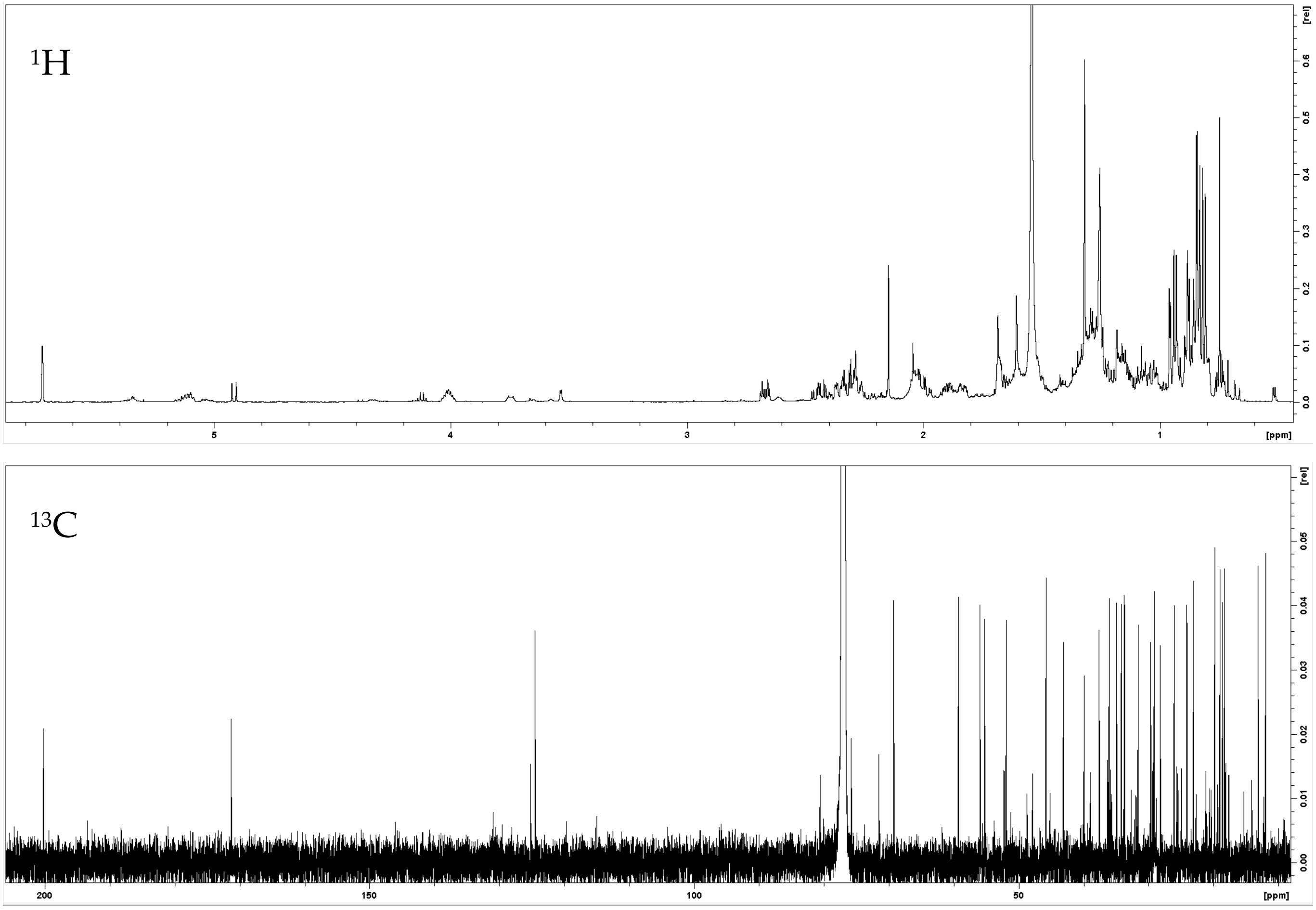

Figure S21. ^{1}H (600 MHz) and ^{13}C NMR (151 MHz) spectrum of compound **39** in $CDCl_3$.

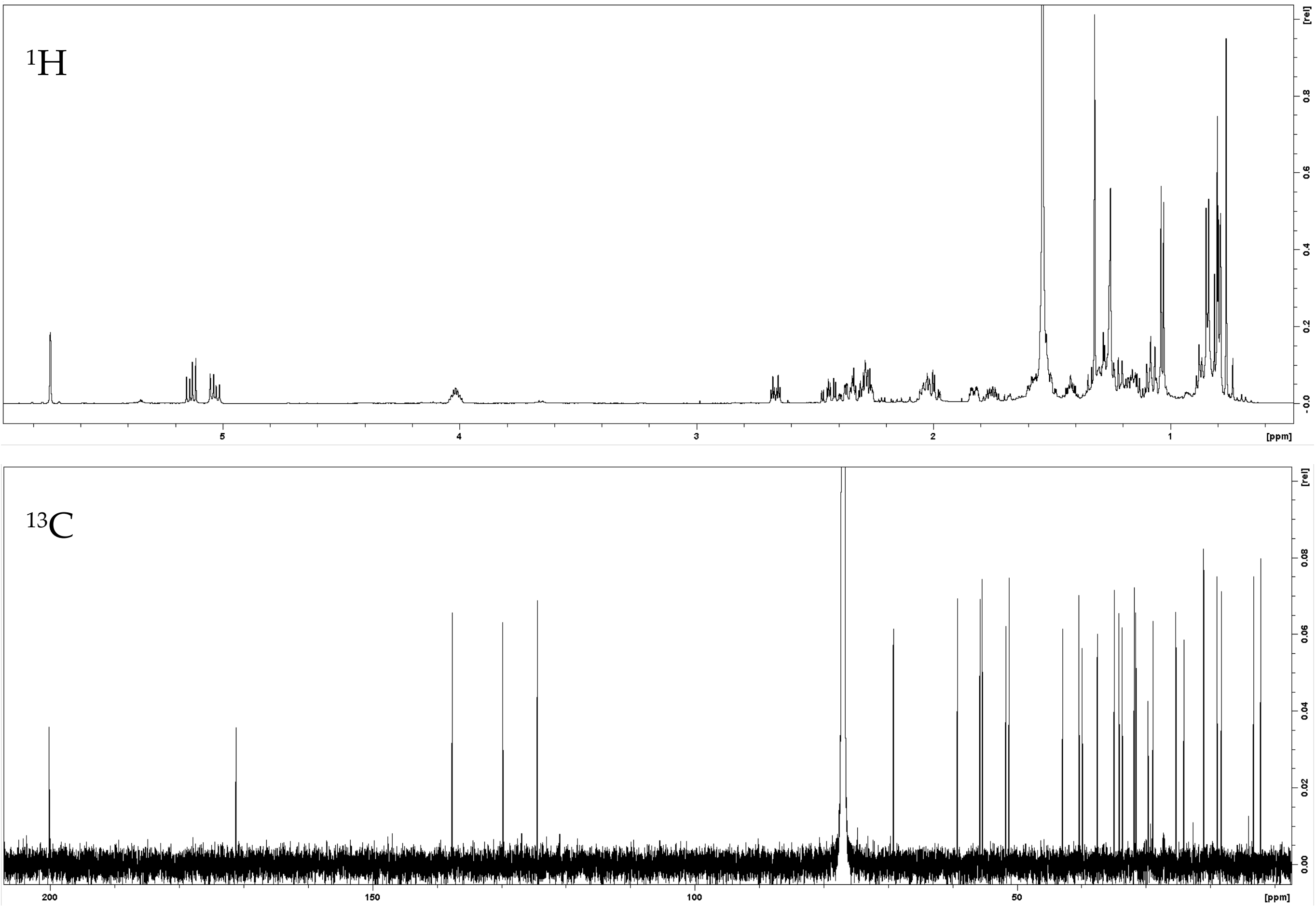

Figure S22. ^1H (600 MHz) and ^{13}C NMR (151 MHz) spectrum of compound **40** in $CDCl_3$.

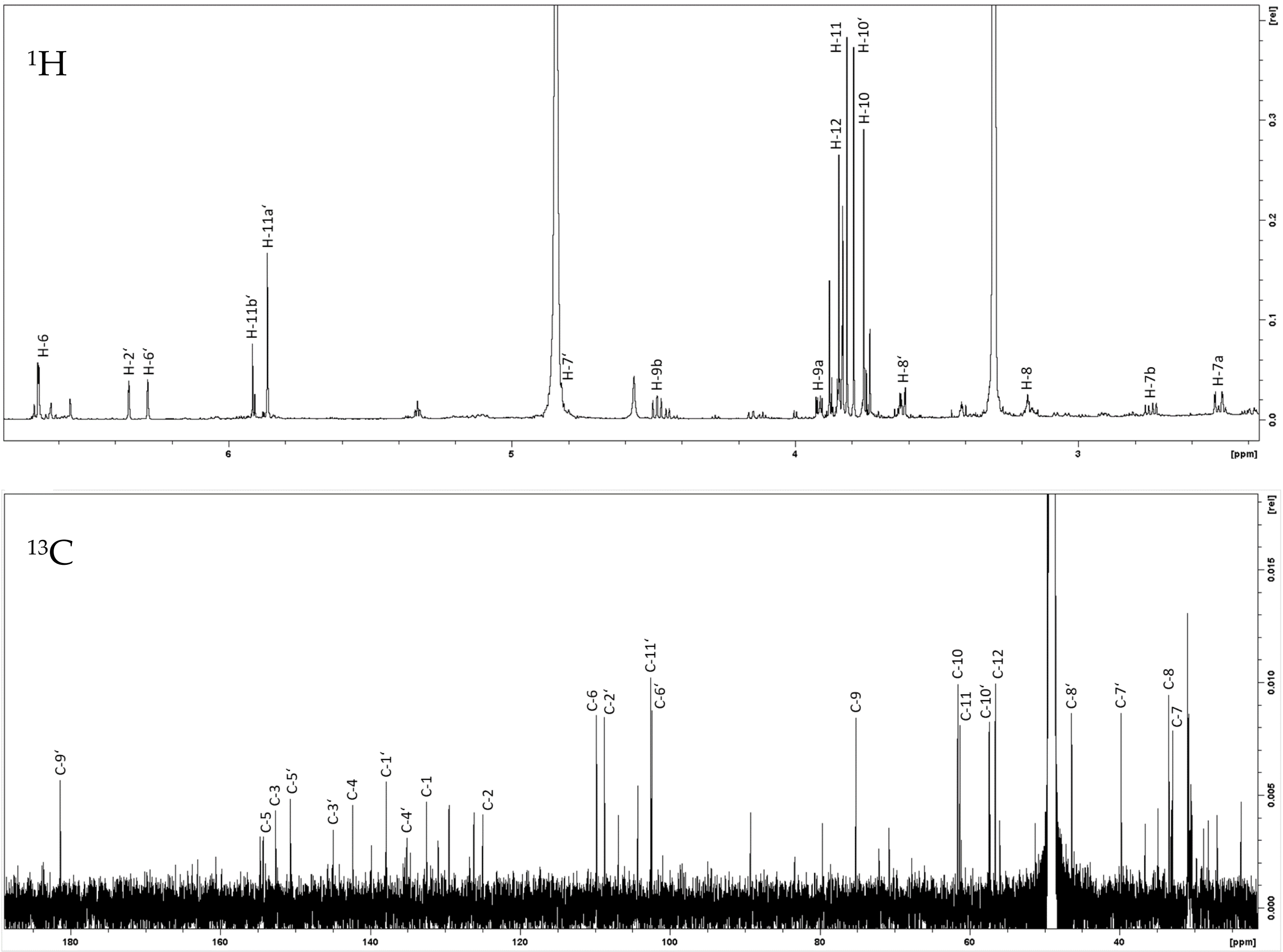

Figure S23. ^{1}H (600 MHz) and ^{13}C NMR (151 MHz) spectrum of compound **43** in CD_3OD.

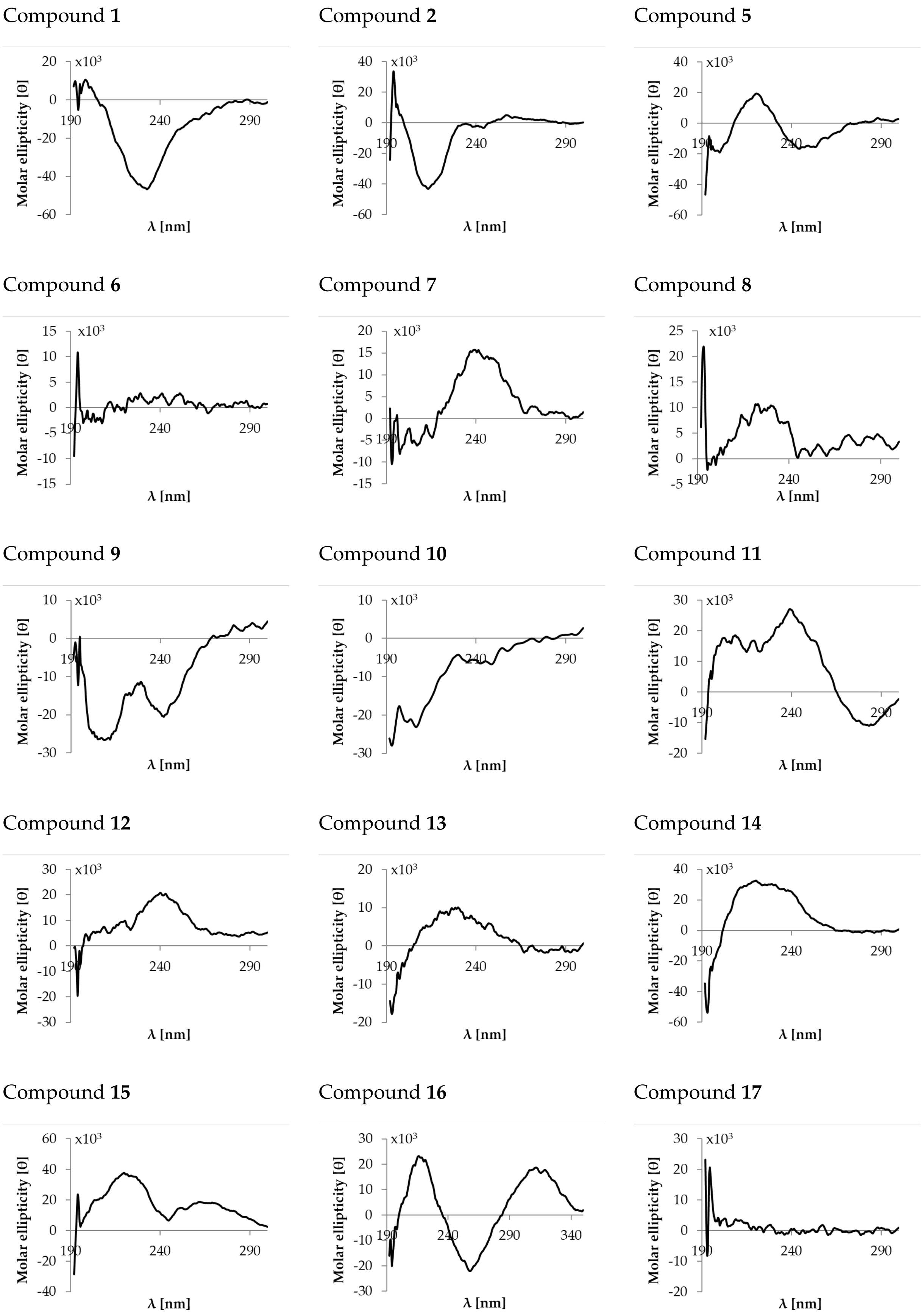

Figure S24. CD spectra of compounds **1**, **2**, **5–17** in MeOH, [θ] in [(°cm^2) x dmol^{-1}].

Compound **19**

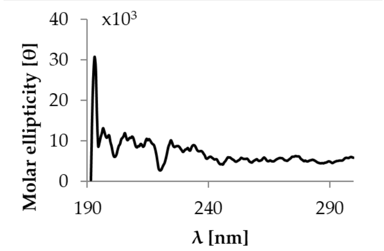

Compound (–)-**21**

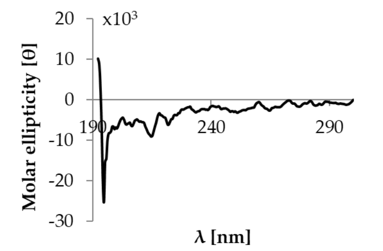

Compound **23**

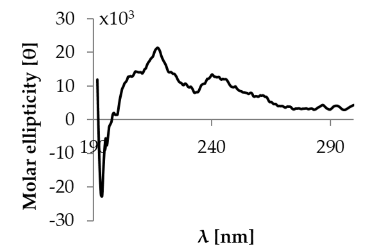

Compound **24**

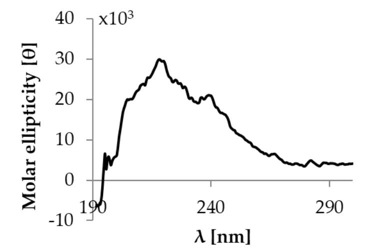

Compound **25**

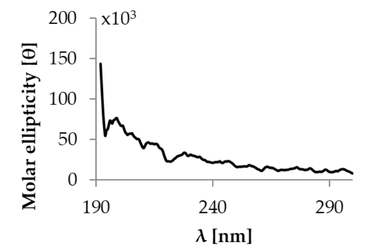

Compound **26**

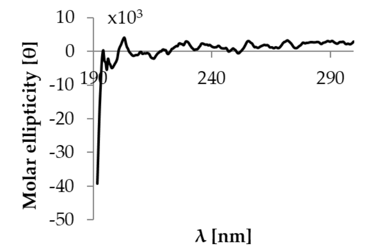

Compound **27**

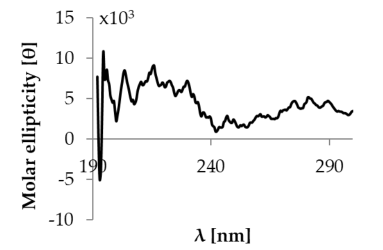

Compound **28**

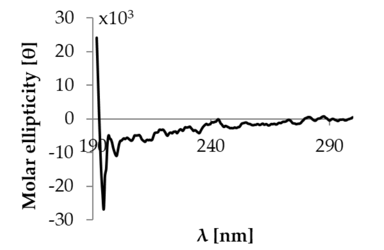

Compound **29**

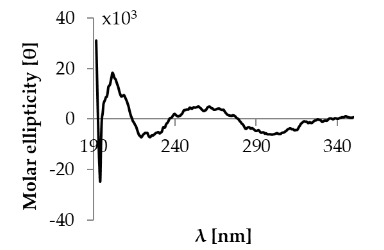

Compound **36**

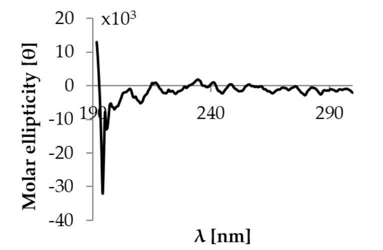

Compound **38**

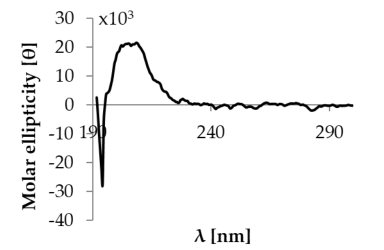

Compound **39**

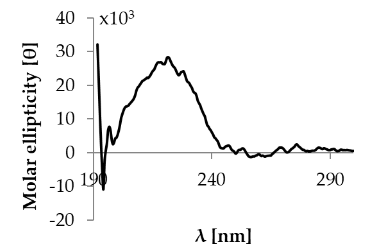

Compound **40**

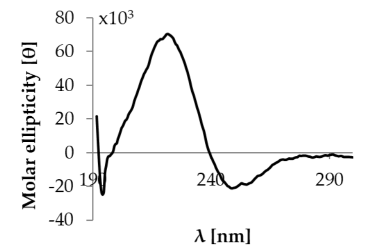

Compound **41**

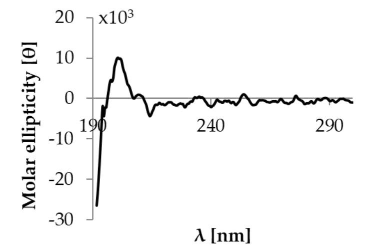

Figure S25. CD spectra of **19**, (–)-**21**, **23**–**29**, **36** and **38**–**41** in MeOH, [θ] in [(°cm^2) x dmol^{-1}].

Compound **42** Compound **43**

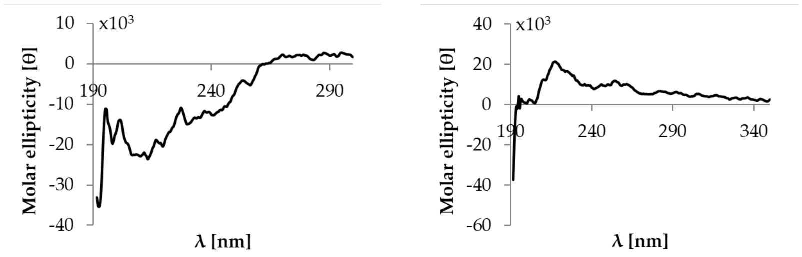

Figure S26. CD spectra of compounds **42** and **43** in MeOH, [*θ*] in [(°cm²) x dmol⁻¹].

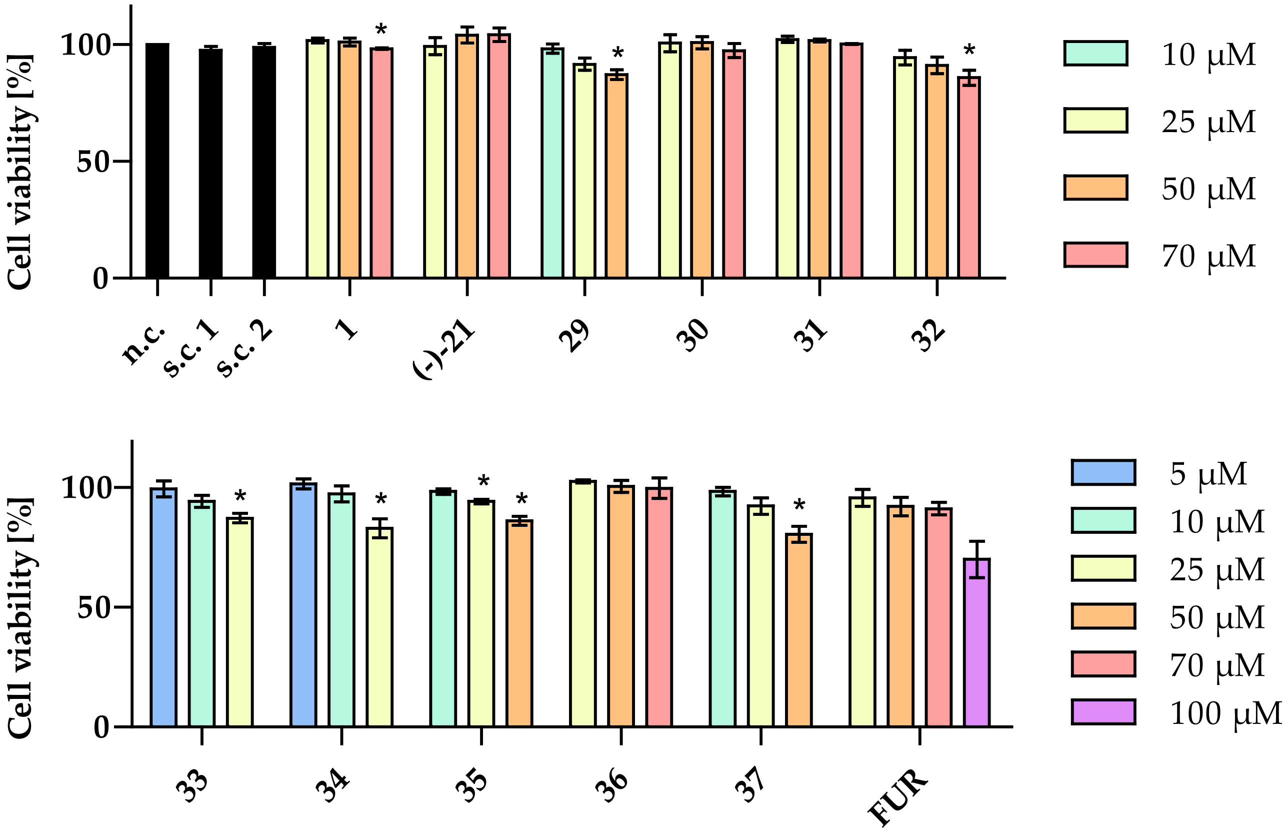

Figure S27. Influence of compounds **1**, (−)-**21**, **29**–**37** and furanoeudesma-1,3-diene (FUR) on the viability of RAW 264.7 cells in the MTT assay. The test was performed including a negative control (n.c., medium only) and two solvent controls (s.c. 1: 0.1% DMSO, s.c. 2: 0,14% DMSO, v/v). Data are presented as mean ± SEM, * $p < 0.05$ vs. n.c. (student's t-test, n=3).

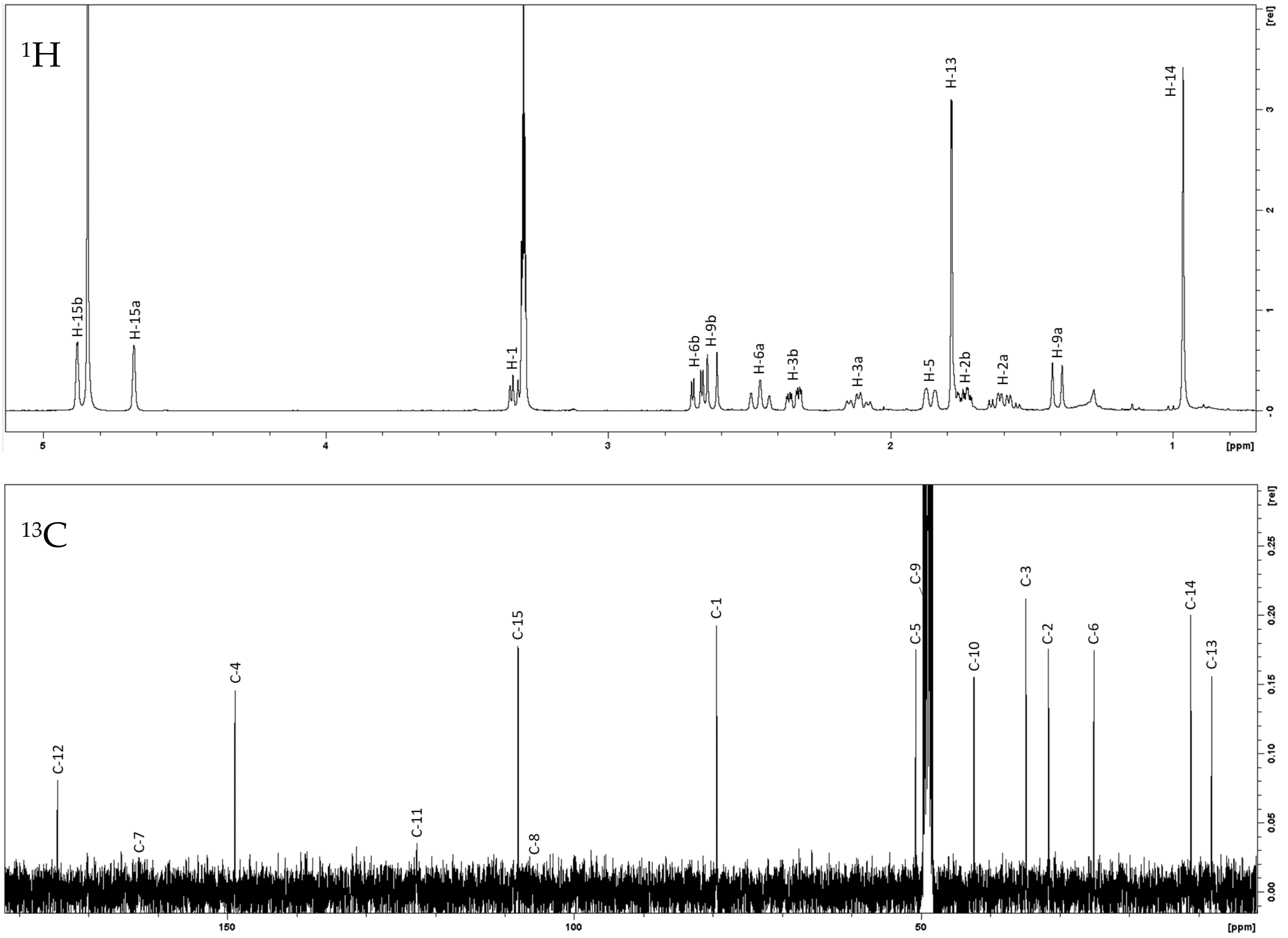

Figure S28. ^1H (400 MHz) and ^{13}C NMR (101 MHz) spectrum of compound (–)-**21** in CD_3OD.

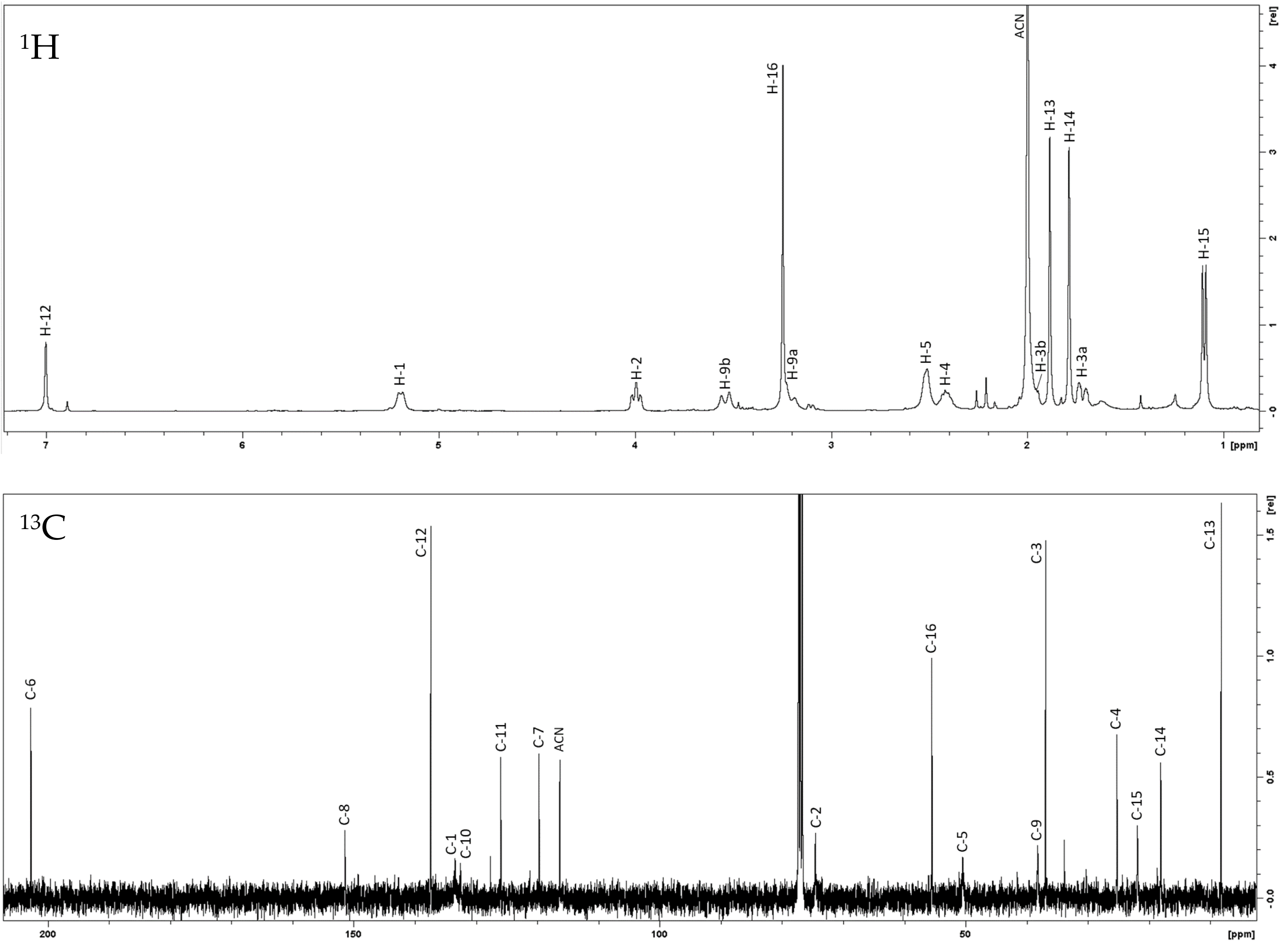

Figure S29. ^{1}H (400 MHz) and ^{13}C NMR (101 MHz) spectrum of compound **29** in $CDCl_3$.

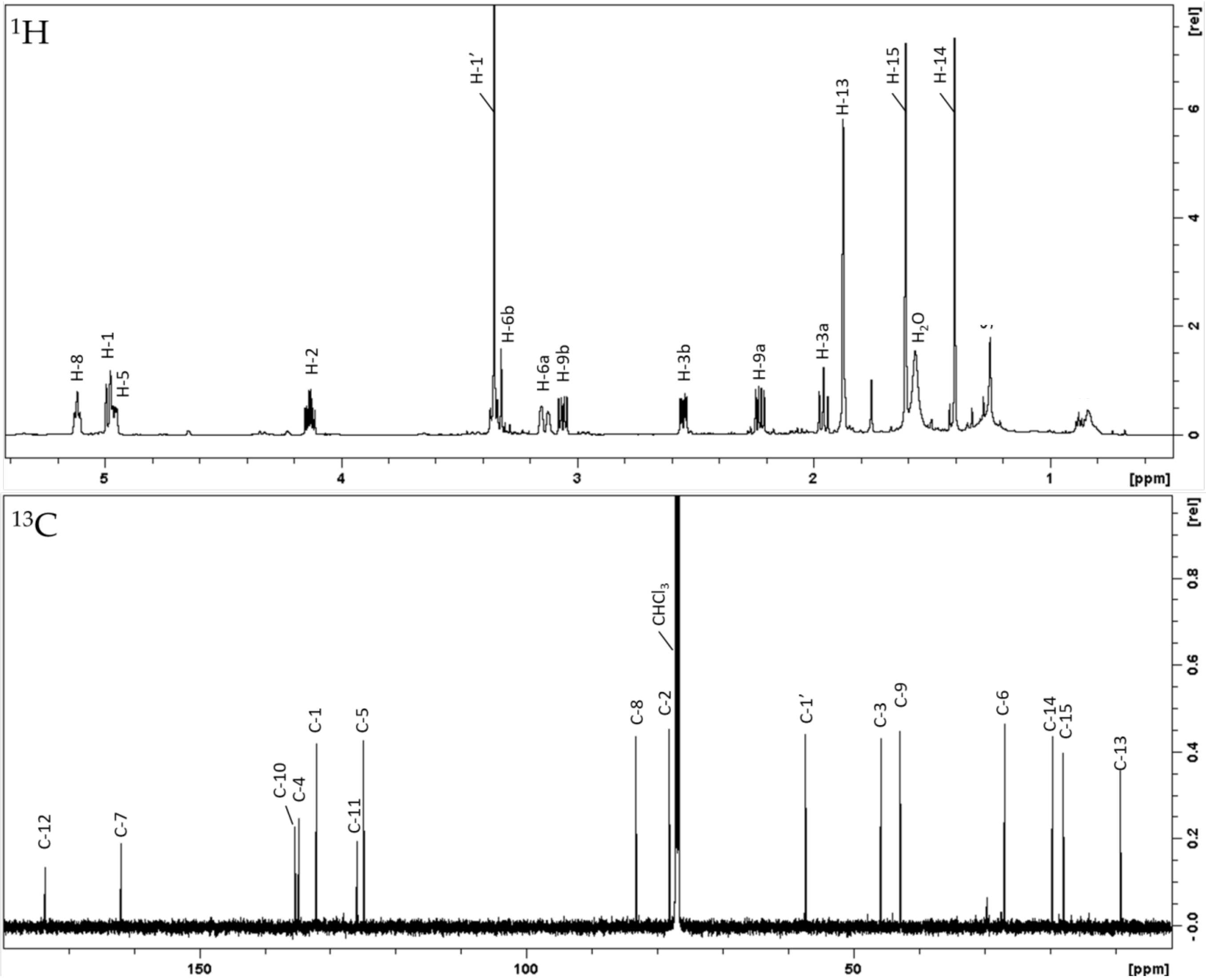

Figure S30. ^{1}H (600 MHz) and ^{13}C NMR (151 MHz) spectrum of compound **30** in $CDCl_3$.

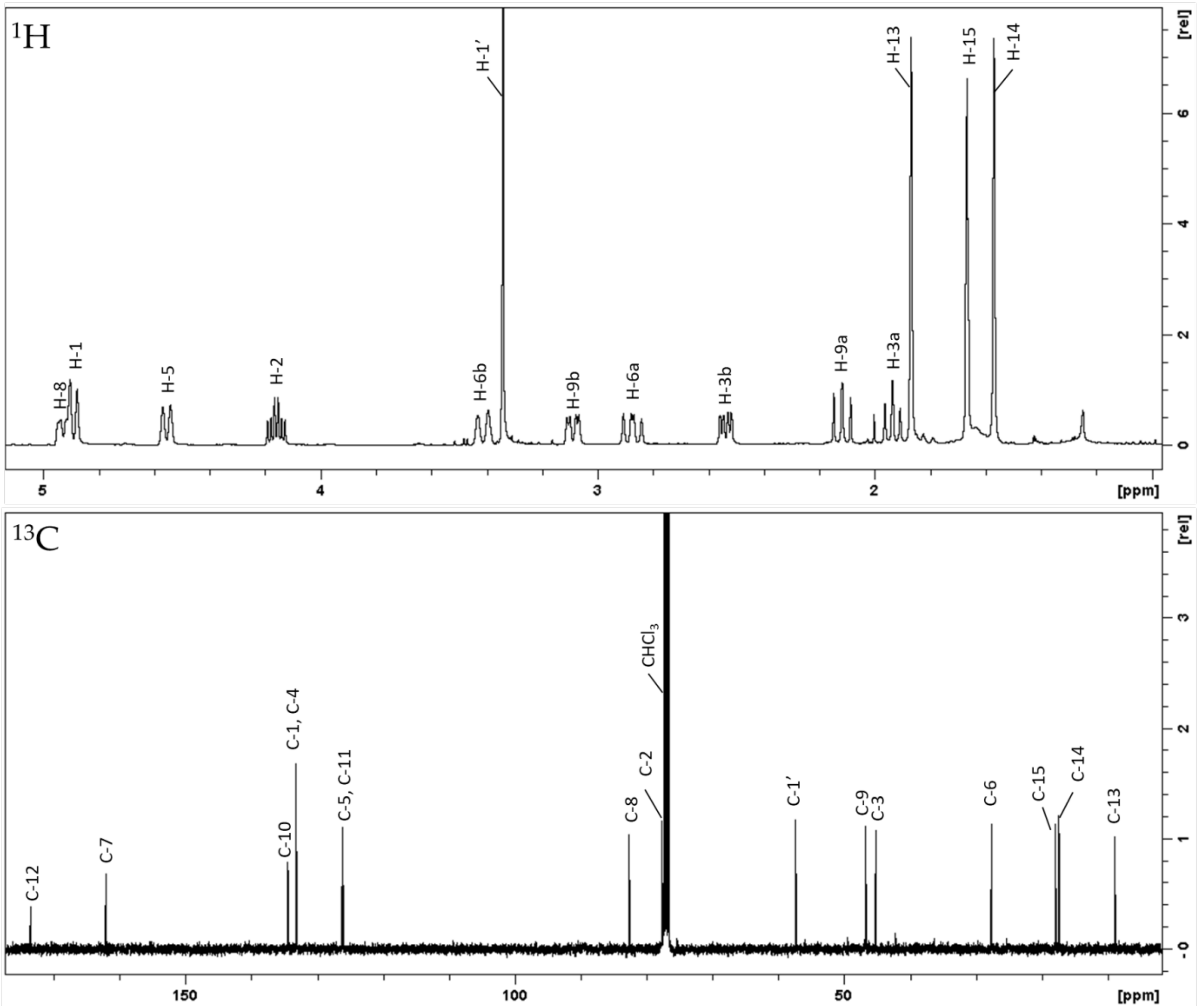

Figure S31. ^{1}H (400 MHz) and ^{13}C NMR (101 MHz) spectrum of compound **31** in $CDCl_3$.

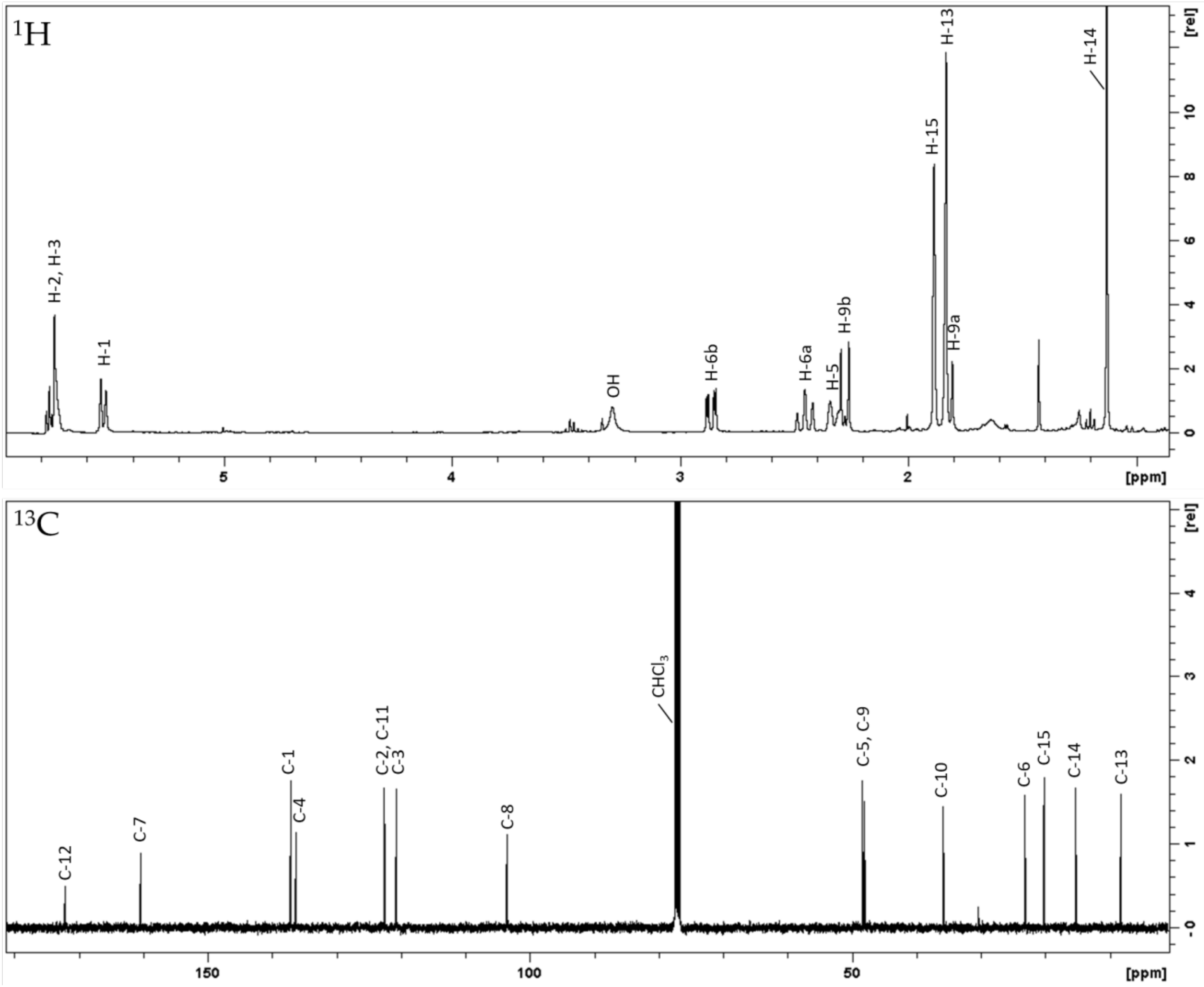

Figure S32. ^{1}H (400 MHz) and ^{13}C NMR (101 MHz) spectrum of compound **32** in $CDCl_3$.

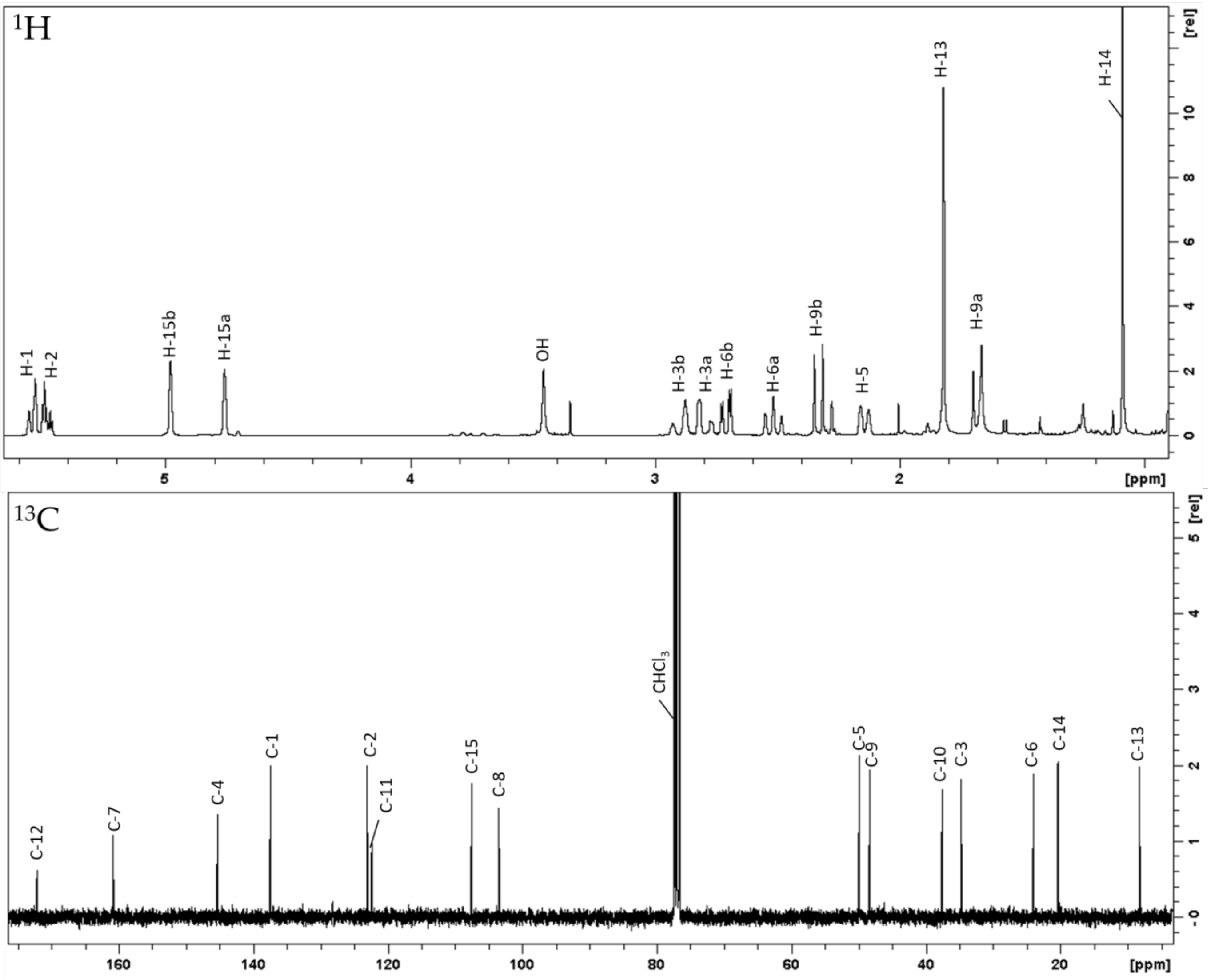

Figure S33. ^{1}H (400 MHz) and ^{13}C NMR (101 MHz) spectrum of compound **33** in $CDCl_3$.

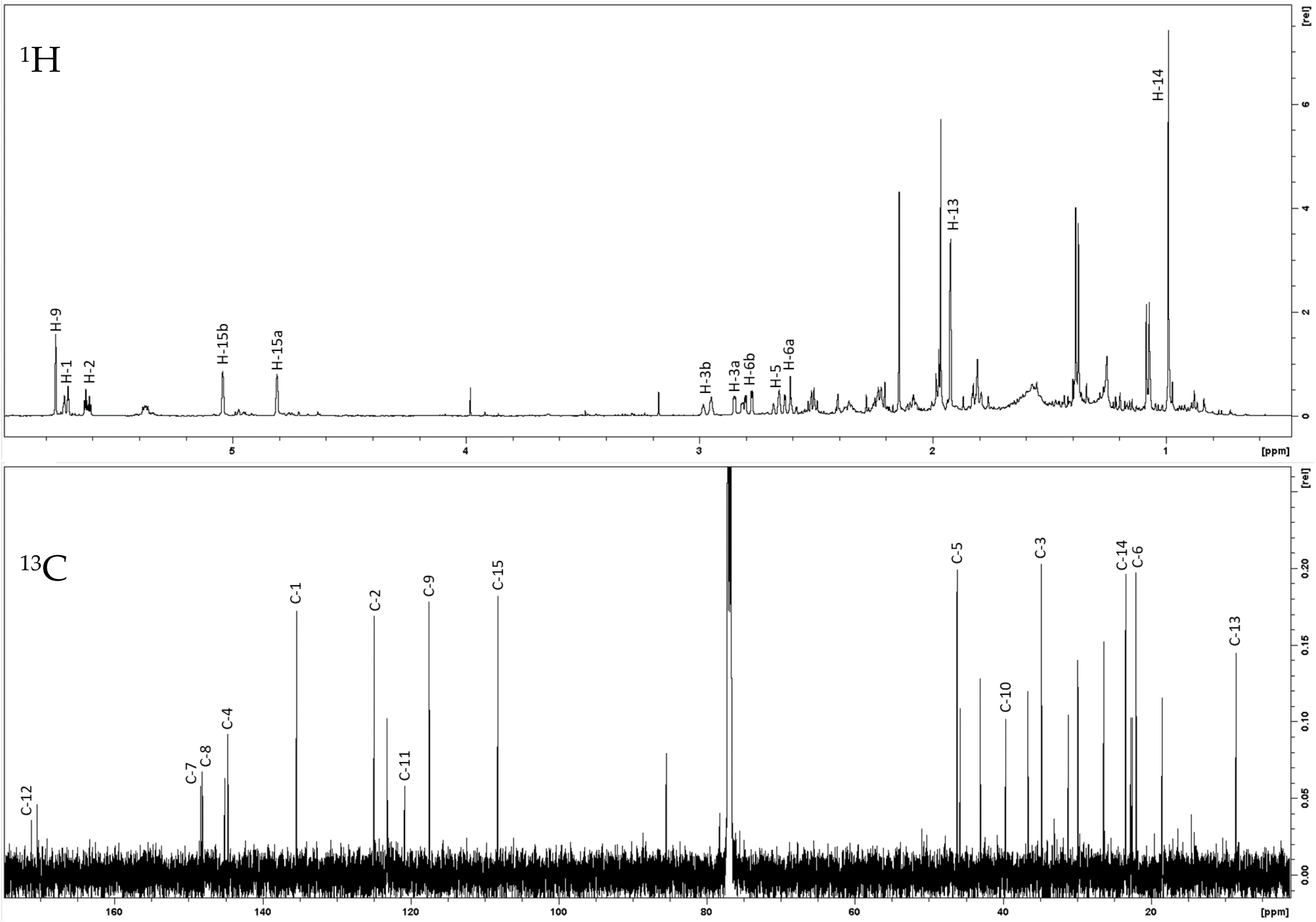

Figure S34. ^{1}H (600 MHz) and ^{13}C NMR (151 MHz) spectrum of compound **34** in $CDCl_3$.

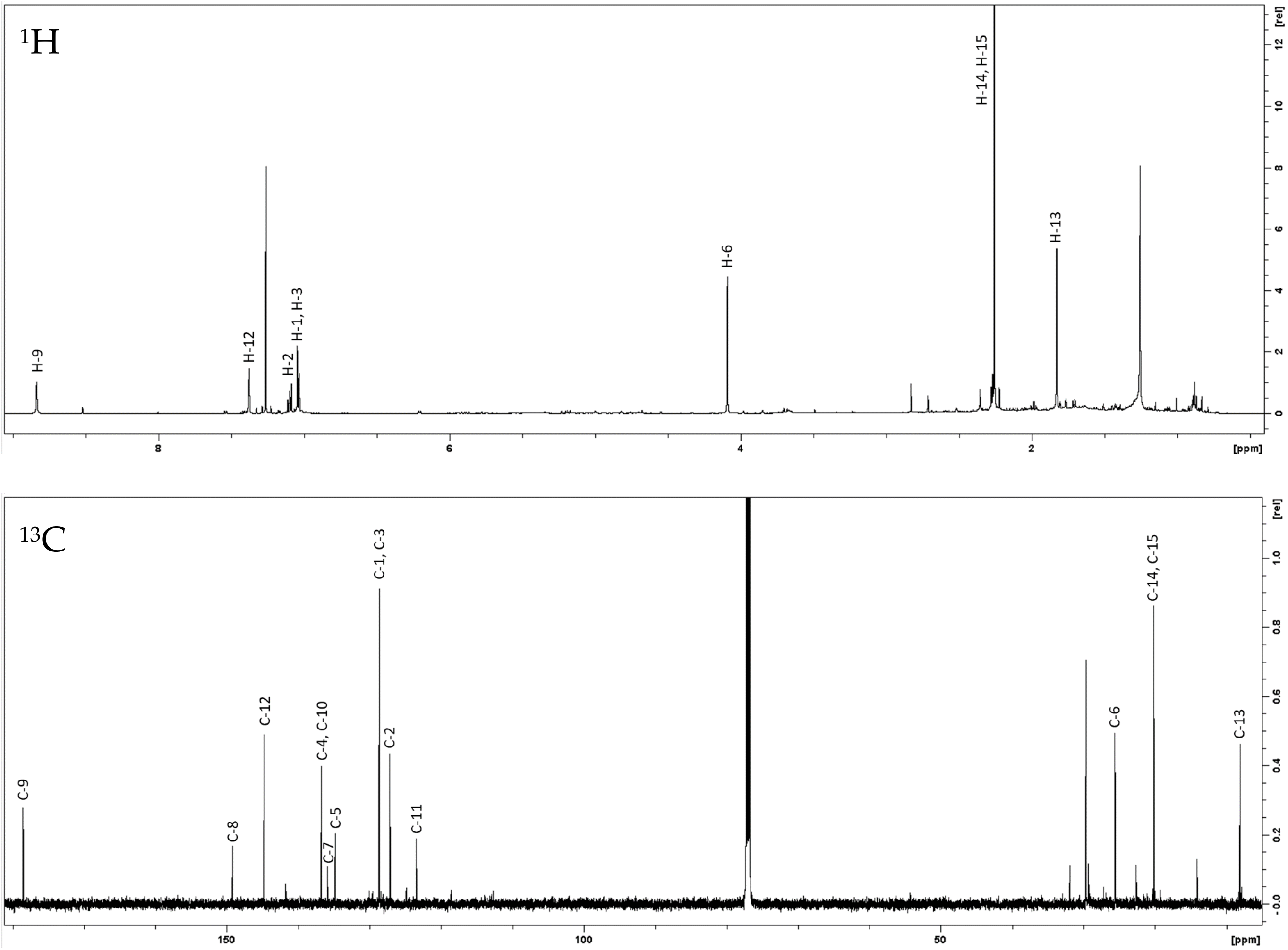

Figure S35. ^1H (600 MHz) and ^{13}C NMR (151 MHz) spectrum of compound **35** in $CDCl_3$.

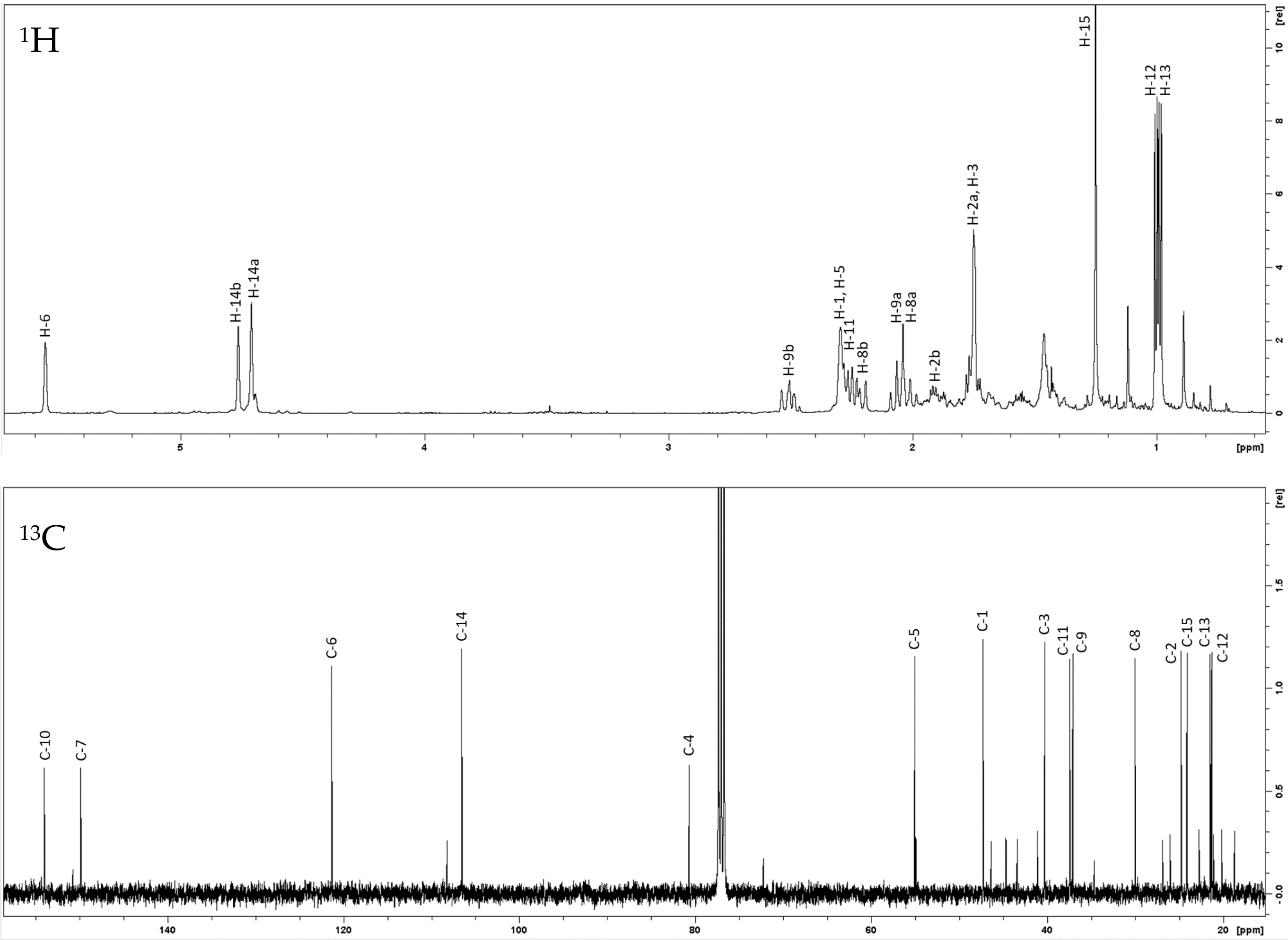

Figure S36. ^{1}H (400 MHz) and ^{13}C NMR (101 MHz) spectrum of compound **36** in $CDCl_3$.

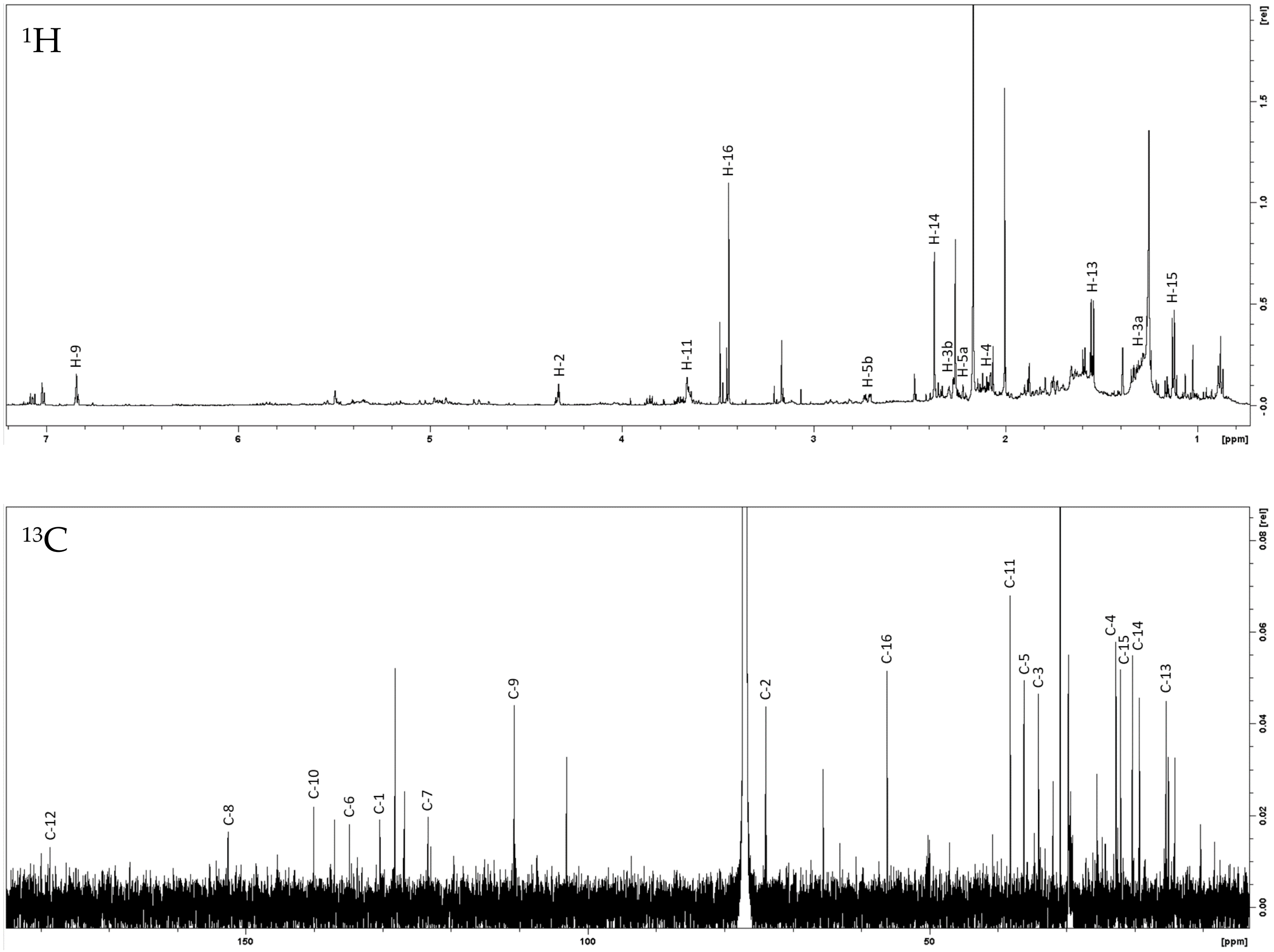

Figure S37. ^{1}H (600 MHz) and ^{13}C NMR (151 MHz) spectrum of compound **37** in $CDCl_3$.

Disclaimer/Publisher's Note: The statements, opinions and data contained in all publications are solely those of the individual author(s) and contributor(s) and not of MDPI and/or the editor(s). MDPI and/or the editor(s) disclaim responsibility for any injury to people or property resulting from any ideas, methods, instructions or products referred to in the content.
